# Protecting the Protectors: Moral Injury, Coping Styles, and Mental Health of UK Police Officers and Staff Investigating Child Sexual Abuse Material

**DOI:** 10.1155/da/1854312

**Published:** 2024-11-23

**Authors:** Paul Conway, Theresa Redmond, Samantha Lundrigan, Deanna Davy, Simon Bailey, Peter Lee

**Affiliations:** ^1^School of Psychology, University of Southampton, Southampton, UK; ^2^School of Criminology and Criminal Justice, University of Portsmouth, Portsmouth, UK; ^3^International Policing and Public Protection Research Institute (IPPPRI), Anglia Ruskin University, Chelmsford, UK

**Keywords:** child sexual assault, coping styles, mental health, moral injury, police

## Abstract

Police officers and staff who investigate child sexual abuse material (CSAM) may be at elevated risk for mental health problems, which may be mitigated or exacerbated by institutional and interpersonal factors. The current work examined mental health in a large sample of UK CSAM investigators (*N* = 661). Results suggest substantially elevated rates of depression and anxiety but not posttraumatic stress disorder (PTSD). Feeling successful and supported powerfully buffered against negative outcomes, whereas moral injury—particularly feelings of institutional betrayal—predicted worse outcomes. Although exposure to CSAM and contact with victims predicted worse outcomes, these effects were much smaller. Regarding coping styles, self-blame, rumination, catastrophizing, withdrawal, ignoring, and negative religious coping predicted worse outcomes, whereas positive refocusing, seeking distraction, and social support were effective. These results held controlling for demographics. These results suggest that UK CSAM police officers and staff experience elevated depression and anxiety, but institutional and interpersonal support can buffer outcomes.

## 1. Introduction

Police investigators and staff dealing with child sexual abuse material (CSAM) have a difficult job. They are often required to view graphic images, videos, and text of young children in harmful sexual situations, interact with victims and their families, or interact with perpetrators [1]. Hence, CSAM investigators may find the environment of their workplace morally contaminating, leading to *moral injury*—damage to the moral self-concept from conducting or witnessing, especially powerlessly witnessing, acts that transgress moral values [2]. Consequently, recent work has called for research on moral injury among police [3], especially considering organizational factors that may exacerbate or buffer moral injury [4].

CSAM investigators are exposed to substantial occupational mental health risks, including posttraumatic stress disorder (PTSD), anxiety, depression, complex trauma, and suicide [5–8]. Yet, it remains unclear which experiences reflect moral injury per se. Moreover, little research on moral injury—or CSAM for that matter—examines cognitive, emotional, behavioral, and religious coping [9–11]. Therefore, there have been calls to clarify how psychological mechanisms impact the influence of moral injury [12]. Finally, most research examining CSAM investigators employs relatively small samples and focuses primarily on stress, trauma, or PTSD [13, 14]. We sought to address these shortcomings by recruiting a larger sample of CSAM investigators and assessing a broader array of measures.

Therefore, we conducted the largest and most comprehensive study (to our knowledge) on mental health among CSAM investigators to date. We recruited 661 UK police officers and staff and examined how moral injury, organizational risk and protective factors, coping styles, and demographics predicted a variety of mental health measures: depression, anxiety, PTSD, and wellbeing. We compared the clinical prevalence of these measures in the current sample compared to other police and civilian samples. We also examined access, perceptions, and barriers to support resources. Finally, we tested preregistered expectations regarding how facets of moral injury, organizational risk and protective factors, and coping styles would predict mental health outcomes and tested for mediation.

### 1.1. Mental Health in CSAM Investigators

Unsurprisingly, people report strong emotional, cognitive, social, and behavioral consequences of working with CSAM, including disgust and anger, nightmares, and flashbacks [1, 15]. Exposure to others' traumatic experiences can produce vicarious trauma and secondary traumatic stress [16]. Unsurprisingly, CSAM investigators often report mental health challenges [6, 17], which can interfere with work duties [18]. Support from peers and supervisors is effective at increasing police wellbeing [19–21], and a sense of job success among CSAM investigators is buffering [22]. Conversely, feeling undermined or unsupported by peers, supervisors, and organizations can undermine wellbeing [6, 23]. Moreover, exposure to CSAM or contact with victims and perpetrators may be challenging [24]. Hence, we developed a novel measure of potential risk and protective factors for CSAM investigators. We expected that risk factors would predict worse mental health outcomes, whereas protective factors would predict better outcomes. 1

### 1.2. Moral Injury

One aspect of mental health that has gained increasing recognition is moral injury. Moral injury entails damage to the self-concept from situations where harm is experienced or observed, which violates an individual's core moral values, and where that person knows the morally right response but is powerless to prevent what is happening [2, 25]. Potentially morally injurious events may not always lead to moral injury. However, when people conceive of events as violating important moral values and link these events to the self-concept, they can experience dissonance between perceptions of the self as morally good and the events in question, leading to a degradation of the moral self.

Such degradation can be toxic. People care deeply about the *moral self-concept*—one's perceived moral worth [26]. Morality forms the core of self- and person-perception [27–29] and helps people decide whether they deserve respect, compassion, and assistance from others [30]. Therefore, damage to the moral self-concept—moral injury—can signal that one is not worthy of participating in society as an equal. Unsurprisingly, therefore, moral injury is associated with a wide variety of negative mental health outcomes, including PTSD, depression, anger, stress, hopelessness, pessimism, and suicidality ([31]; Maguen and Litz [32, 33]).

### 1.3. Moral Injury in CSAM Investigators

Moral injury research began by examining war veterans [2], with most work still focusing on this population, especially in America and Canada [34]. However, research has begun to document moral injury in other populations. Papazoglou and Chopko [3] called for research on moral injury in police specifically. Recent work demonstrated that moral injury in Finnish police is associated with PTSD, compassion fatigue, and reduced compassion satisfaction [7, 35, 36], and trauma-exposed Dutch police with higher PTSD symptoms showed comorbidity with moral injury [37]. Therapy for moral injury in the police has moderate success [38].

Fewer studies have examined police working with CSAM. Doyle et al. [6] found that moral injury and trauma in CSAM investigators were associated with excessive workloads, mental health-related stigma within policing culture, and a lack of bespoke therapeutic support among CSAM investigators. Tapson et al. [39] found that moral injury was associated with negative coping styles like substance use, avoidance, emotional numbing, and anxiety. Together, these studies sketch a picture of poor mental health among police and especially CSAM investigators. Yet, these studies largely employed small, qualitative samples and derived from only a handful of datasets. Moreover, each measured relatively few variables—hence, our goal of expanding sample size and measurement diversity.

Indeed, research on moral injury itself has been somewhat conceptually limited. Most studies examine associations of moral injury with PTSD and related variables [34]. Accordingly, Frankfurt and Frasier [12] called for deepening conceptual understanding by examining risk and protective factors, measuring constructs beyond PTSD, and considering mechanisms like coping styles. Therefore, we examined how moral injury predicted a variety of mental health outcomes, including mediation via coping styles. We also examined workplace risk and protective factors. Simmons-Beauchamp and Sharpe [4] noted that moral injury may spring from ineffective police leadership, so feelings of betrayal by supervisors and colleagues may play an important role. Conversely, effective leadership may buffer the impact of potentially morally injurious events on mental health.

We examined three facets of moral injury, following Bryan et al. [31]: *transgression-self*—performing morally questionable acts; *transgression-other*—witnessing immoral acts; and *betrayal*—a sense that colleagues, supervisors, and institutions demonstrate moral failings. We anticipated replicating Bryan et al.s' finding that all three measures would uniquely predict outcomes. Specifically, we predicted that transgressions-other and betrayal would predict PTSD more than transgressions-self. Unlike Bryan et al. [31], but in line with Nash et al. [40], we expected transgression-other would positively predict depression.

### 1.4. Cognitive, Emotional, Behavioral, and Religious Coping Styles

Researchers have documented ways police and other professionals cope with CSAM, describing organizational, social, and psychological support, and strategies such as humor [1, 24, 41, 42]. However, research often relies on qualitative interviews, so the effectiveness of such strategies remains unclear. What little quantitative evidence exists for positive coping strategies is weak [19, 43]—except for empathy [44] and lighthearted humor [45], which robustly predict positive outcomes. Conversely, there is clearer support for the damaging effects of negative coping strategies like denial, alcohol and tobacco use [5], and withdrawing from family and friends [46]. However, few studies ground research in psychological models of coping. Parkes, Graham-Kevan, and Bryce [47] examined coping styles like detachment, avoidance, and process-driven coping but did not examine the effectiveness of these coping styles.

Therefore, we drew upon theories of emotional, cognitive, and behavioral coping strategies [9, 10]. These include positive strategies like positive reappraisal, seeking distraction, and social support, which predicted reduced depression and anxiety. These also include negative strategies like self-blame, rumination, catastrophizing, withdrawal, and ignoring, which predicted increased depression and anxiety. We expected similar patterns for depression and anxiety in the current study. We extended such predictions to PTSD and anticipated the opposite pattern for wellbeing. Moreover, we tested whether these coping styles mediated the effect of moral injury on each outcome.

Finally, CSAM may be especially challenging for people with strong religious values due to the dissonance between their faith and morally compromising events [48]. Therefore, we included a measure of positive and negative religious coping [11]. In traumatic situations, people may draw strength from faith (positive religious coping) or perceive divine punishment (negative religious coping). Positive coping may predict wellbeing [11], but military research suggests that only negative coping predicts mental health outcomes [49, 50]. Therefore, we predicted that negative religious coping would prove more impactful than positive religious coping for negative outcomes: depression, anxiety, and PTSD, but positive religious coping would predict wellbeing. Again, we tested mediation models.

### 1.5. The Current Work

We recruited 661 currently practicing police officers and staff dealing with CSAM from every constabulary across the United Kingdom. We assessed risk and protective factors, moral injury, religious, emotional, cognitive, and behavioral coping styles, and outcomes: depression, anxiety, PTSD, and wellbeing, plus demographics.

## 2. Method

### 2.1. Transparency and Openness

We preregistered the study: https://osf.io/x36rd/?view_only=55d032a515264b6dbe381d44f53a361d. We obtained ethics approval from the University of Portsmouth Faculty of Creative and Cultural Industries Ethics Committee, Reference CCI-FEthC 2022-003. We report all materials and measures, how we determined sample size and data exclusions. We analyzed data via SPSS 28.0 [51]. All data, analyses, and materials are available: https://osf.io/5ru7h/?view_only=5f5f066819ec436ab547533977feaf00.

Note that, as preregistered, we originally recruited 896 participants from England and Wales only, analyzing 575 who passed exclusion checks. Due to police requests to broaden participation, we updated preregistration and reopened the sample to Scotland and Northern Ireland. Although we added participants to increase representation rather than for statistical reasons, this might inflate Type I error [52]. To counteract such inflation, we preregistered a conservative criterion of *α* = 0.025. Analyses of the original and final samples were similar Supporting Information 1: File S1. We also preregistered parallel analyses on a comparison sample of online UK residents via Prolific. Patterns were again largely similar Supporting Information 2: File S2.

Our preregistered power analysis suggested *N* > 236, but we committed to recruiting as many as possible. A sensitivity analysis in G^*∗*^ Power [53] showed that 661 provides 95% power to detect a small effect of Cohen's *f*^2^ = 0.04 at *α* = 0.025 in the most complex linear multiple regression analysis in this study with 9 predictors and 10 control variables [54].

### 2.2. Participants

We recruited 1056 police officers and staff via multiple recruitment emails Supporting Information 3: File S3 sent by commanding officers across forces in England, Wales, Scotland, and Northern Ireland, supported by the National Police Chief's Council (NPCC) lead for Child Protection and Abuse Investigations. We removed 15 who refused reconsent, 156 who completed <50%, 38 who completed the study in <200 s, and 186 who failed an attention check [55], leaving a final sample of 661 (see Section 3 for additional details).

### 2.3. Procedure and Materials

Participants completed all measures online via Qualtrics [56]; Supporting Information 3: File S3. In addition to the measures reported here, we examined perceptions of resources and barriers (see [23] or Supporting Information 4: File S4). See Supporting Information 5: Appendix A: Data Transparency for additional details regarding the overlap between these manuscripts.

#### 2.3.1. Demographics

Participants reported their age, gender, ethnicity, whether they are a *police officer* or *police staff*, rank (if police) or job title (if civilian), their region of work and role, their experience in both their current role and entire career from 1 (*only a few days*) to 9 (*over 20 years*). They also reported marital status, whether they are a parent or caregiver to children under 18 and people over 18 (*yes/no/it's complicated*), and spirituality/religiosity on a scale from 1 (*not at all spiritual/religious*) to 7 (*extremely spiritual/religious*), plus which faith they most identify with, and whether they have ever been in therapy (open-ended).

#### 2.3.2. Risk and Support Factors

Participants reported risk and support factors on scales from 1 (*almost never*) to 7 (*almost always*). As preregistered, we conducted a principal components analysis (PCA) with oblimin rotation with 500 iterations for convergence and rotation, retaining interpretable [57] factors with eigenvalues >1 [58]. We retained items loading >0.5 on a factor and <0.3 on another (two items were discarded), revealing three factors (eigenvalues 3.74, 2.90, 2.17) explaining 62.94% of the variance. Although we preregistered an anticipated solution, the results suggested a different solution.

Four items reflected contact with victims and perpetrators (*α* = 0.91), e.g., *Contact* (*in person*, *phone*, *internet*) *with victims*, two reflected exposure to CSAM (*α* = 0.98), e.g., Vie*wing*, *grading*, *or handling photos of child sexual abuse and exploitation* (*CSAE*), and six reflected job success and support (*α* = 0.77), e.g., *Feeling supported by my supervisor or team*, *Feeling my work is valuable and meaningful*. We computed the mean of each subscale.

#### 2.3.3. The Moral Injury Events Scale

Participants completed a slightly adapted version of the moral injury events scale (MIES, [40]) regarding their experiences investigating CSAM on scales from 1 (*strongly disagree*) to 7 (*strongly agree*). We conducted a similar PCA, producing three factors (eigenvalues 4.21, 1.38, 1.19) explaining 75.16% of the variance, consistent with preregistration and Bryan et al. [31]. Two items loaded on *Transgressions-Other* factor (e.g., *I saw things that were morally wrong*, *α* = 0.68), four loaded on *Transgressions-Self* factor (e.g., *I acted in ways that violated my own moral code or values*, *α* = 0.90), and three loaded on *Betrayal* (e.g., *I feel betrayed by colleagues who I once trusted*, *α* = 0.78). Moreover, as overall scale reliability was higher than *α* = 0.7 (*α* = 0.82), we employed the full scale for most analyses as preregistered.

#### 2.3.4. Short Cognitive and Emotion Regulation Questionnaire (CERQ)

Participants completed the 18-item short CERQ [9], consisting of two items for each of nine constructs on scales from 1 (*almost never*) to 7 (*almost always*). Items assessed *self-blame* (*α* = 0.71), *other-blame* (*α* = 0.67), *rumination* (*α* = 0.72), *catastrophizing* (*α* = 0.76), *positive refocusing* (*α* = 0.83), *planning* (*α* = 0.62), *positive reappraisal* (*α* = 0.69), *putting into perspective* (*α* = 0.66), and *acceptance* (*α* = 0.76). For positive reappraisal, we selected two items from the original CERQ not included in the short version, *I think that the situation also has its positive sides*, and *I look for the positive sides to the matter*, referring to positively about the situation rather than self, which may be appropriate to CSAM settings.

#### 2.3.5. Behavioral Emotion Regulation Questionnaire (BERQ)

Participants completed the two best-loading items for each factor from Table 1 in Kraaij and Garnefski [10] on the same scale as the CERQ: *seeking distraction* (*α* = 0.77), *withdrawal* (*α* = 0.90), *actively approaching* (*α* = 0.58), *seeking social support* (*α* = 0.82), and *ignoring* (*α* = 0.75).

#### 2.3.6. Brief Religious Coping Scale (RCOPE)

Participants considered “whatever the divine meant to them” and reported *positive coping* (*α* = 0.96, e.g., *Focused on religion to stop worrying about my problems*) and *negative coping* (*α* = 0.86, e.g., *Questioned God's love for me*) on scales from 1 = *Almost never* to 7 = *Almost always* [11] A similar PCA found the expected two factors (eigenvalues 7.92, 2.24) accounting for 72.65% of variance. All items loaded as expected, except *I decided the devil made this happen*, which loaded higher on the positive than negative scale, so we included it there.

#### 2.3.7. Patient Health Questionnaire-9 (PHQ-9)

Participants completed the PHQ-9 depression measure, which asks about nine symptoms over the past month (e.g., *little interest or pleasure in doing things*) on scales where 0 = *not at all*, 1 = *several days*, 2 = *more than half the days*, 3 = *nearly every day* [59], plus one item asking about how difficult symptoms make daily life (1 = *Not difficult at all–*4 = *very difficult*). We computed the mean (*α* = 0.90) and sum to group people into clinical categories.

#### 2.3.8. Generalized Anxiety Disorder-7 (GAD-7)

Participants rated seven anxiety symptoms the past month (e.g., *trouble relaxing*) on the same 4-point scale as the PHQ-9 [60]. We computed the mean (*α* = 0.91) and sum to group people into four clinical categories.

#### 2.3.9. PTSD: The International Trauma Questionnaire

Participants reported 12 PTSD and complex PTSD (C-PTSD) symptoms on scales from 0 (*not at all*) to 4 (*extremely*). Six PTSD (e.g., *Feeling jumpy or easily startled*) items reflect *Reexperiencing α* = 0.67, *Avoidance α* = 0.86, and *Sense of Threat α* = 0.68, and six disturbance in self-organization items (e.g., *I feel numb or emotionally shut down*) reflect *Affect Dysregulation α* = 0.61,*Negative Self-Concept α* = 0.91, and *Disturbances in Relationships α* = 0.88. Three items for each cluster measured functional impairment (e.g., *How much have these symptoms affected your relationships or social life?*). The main analysis averaged across all items (i.e., C-PTSD; *α* = 0.94); follow-up analyses separately examined the PTSD (*α* = 0.90) and disturbance in self-organization clusters (*α* = 0.93). We also computed criteria for PTSD and C-PTSD diagnosis.

#### 2.3.10. Wellbeing: Schwartz Outcome Scale-10 (SOS-10)

To examine positive outcomes beyond the absence of mental health problems [61], participants completed a 10-item measure of wellbeing, the SOS-10 (e.g., *I have peace of mind*), on scales from 1 (*never*) to 7 (*all or nearly all of the time*, [62]). We computed the mean (*α* = 0.94).

## 3. Results

### 3.1. Overview

First, we report sample characteristics, then the clinical prevalence of depression, anxiety, and PTSD. Next, we examined correlational and regression analyses of predictors of mental health outcomes. Finally, we present mediation analyses examining how coping styles mediate variance from moral injury to mental health outcomes.

### 3.2. Sample Characteristics

We recruited 467 police officers and 194 police staff, *M*_age_ = 40.61, *SD* = 9.15, 281 male (42.5%), 377 female (57.0%), and three unreported. Regarding marital status, 372 reported *married* (56.3%), 142 reported *single* (21.5%), 93 reported *living as married* (14.1%), 33 *divorced* (5.0%), 18 *separated* (2.7%), 1 *widowed* (0.2%) and two (0.3%) unreported. We recorded responses as 465 (70.3%) *partnered* and 196 (29.7%) *unpartnered*. The majority (*n* = 642, 97.1%) identified as *White*, *British*, or *Caucasian*, with 6 reporting *Mixed*, 2 reporting *Black or Caribbean*, 3 as *Asian or Indian*, 2 as Arabic, and 6 did not report ancestry.

Regarding parental status, 371 (56.1%) reported they were a parent or guardian for children under 18, 280 (42.4%) reported they were not, and 10 (1.5%) reported *it is complicated*, clarifying, e.g., “currently pregnant” (recorded as living with children). Regarding caregiving status for someone over 18, 605 (91.5%) reported they were not, 50 (7.6%) reported they were, and five (0.7%) reported *it is complicated* (recoded as caregiving). Regarding therapy, 426 participants (64.4%) reported never receiving therapy, whereas 228 (34.5%) reported they were currently or previously in therapy; 7 (1.1%) did not respond (coded no).

Few participants reported strong spiritual or religious beliefs (*M* = 2.01 on a 1–7 scale, *SD* = 1.38). Regarding faith, 297 (44.9%) identified as atheist or no religion, 249 (37.6%) as Christian, 29 (4.4%) as spiritual, 22 (3.3%) as Catholic, 22 (3.3%) as agnostic, 19 (2.9%) as Church of England (3%), 3 (0.5%) as Humanist, 3 (0.5%) as Muslim, 2 (0.3%) as Pagan, 1 (0.2%) as Sikh, 1 (0.2%) as Jewish, 1 (0.2%) as Methodist, 1 (0.2%) as Buddhist, 1 (0.2%) as Shinto, and 10 (1.7%) did not report a religious affiliation.

Participants reported an average career length of 10–14 years (*M* = 7.21, *SD* = 1.54 on a 1–9 scale) and an average in their current role of over 2 years (*M* = 4.77, *SD* = 1.25, same scale). Participants served all regions of the United Kingdom (Table 1). All were currently active members of UK police forces or police staff who regularly deal with CSAM, except for 18 who reported recent service (who we retained). Participants served in a variety of roles (Table 2). Participants described themselves as *Detective Constable*, *Detective Inspector*, *Detective Sergeant*, *case workers*, *case review officers*, *offender managers*, *computer forensic specialists*, or other specialist positions.

### 3.3. Clinical Prevalence

#### 3.3.1. Depression

We summed PHQ-9 items to group people into five clinical categories [59, 63]: >5 = *minimal*, 5–9 = *mild*, 10–14 = *moderate*, 15–19 = *moderately severe*, and 20–17 = *severe*, with ≥10 = probable major depression. We found 183 (27.7%) may qualify for major depression, with 75 (11.4%) reporting moderately severe or severe symptoms (Table 3).

#### 3.3.2. Generalized Anxiety

We summed GAD-7 items to group people into four clinical categories [60]: >5 = *minimal*, 5–9 = *mild*, 10–4 = *moderate*, and 15–21 = *severe*, where ≥10 “represents a reasonable cut point for identifying cases of GAD” (p. 1096). We found that 160 (24.2%) may qualify for generalized anxiety disorder (Table 4).

#### 3.3.3. PTSD and C-PTSD

We computed clinical criteria for PTSD and C-PTSD diagnoses. PTSD requires ≥2 on at least one symptom for each PTSD subscale, plus ≥2 on functional impairment. C-PTSD requires the same, plus ≥2 or higher on at least one symptom for each disturbance in the self-organization subscale plus ≥2 on functional impairment. We also computed the mean for each subscale and across all items. We found that 22 (3.3%) met the criteria for clinical PTSD and 36 (5.4%) for C-PTSD, totaling 8.7% (Table 5).

#### 3.3.4. Wellbeing

Although the SOS does not have clinical categories, we nonetheless compared mean results (*M* = 4.70, *SD* = 1.11) to other samples. 2 This was somewhat lower than American undergraduates, *M*_1_ = 5.73; *M*_2_ = 5.53 [64], or mental health professionals, *M* = 5.28 [65], but comparable to counseling center clients, *M*_1_ = 4.9, *M*_2_ = 4.47 [64], and higher than people with severe psychiatric disorders, *M* = 3.9 [62]. We interpret such comparisons with caution, but results suggest CSAM investigators may score somewhat lower than typical samples and more in line with troubled people.

### 3.4. Correlational and Regression Analyses

We reported bivariate correlations between all measures (Table 6a,b,c) but focused on interpreting regressions to assess the unique predictive power of each variable controlling for demographics. We conducted a series of linear multiple regressions from (standardized) theoretical predictors on moral injury, anxiety, depression, PTSD, and wellbeing [66]. Each analysis examined theoretical predictors (at step 2) controlling for demographics at step 1 (age, gender, officer versus staff, career length, role length, relationship status, parent status, caregiver status, therapy experience, and religiosity). The theoretical predictors were (a) risk and protective factors, (b) the MIES, (c) CERQ, (d) BERQ, and (e) RCOPE. We also conducted follow-up analyses on PTSD subsections.

#### 3.4.1. Demographic Predictors

Most demographic variables failed to predict significant variance in any outcomes, with two exceptions. First, therapy experience: people who reported attending therapy (about a third of the sample), whether previously or currently, tended to report higher moral injury, depression, anxiety, PTSD, and reduced wellbeing than people who never attended therapy. Second, religiosity: people who reported greater religiosity also reported higher moral injury, depression, PTSD, and reduced wellbeing, plus a pattern of higher anxiety that did not always reach significance.

As preregistered, we computed *t*-tests to examine how dichotomous demographic variables predicted outcomes. Results demonstrated no significant difference between people in a relationship versus not on any outcome measure at our preregistered level of significance (*p* < 0.025), with the lowest *p*=0.028. Parents reported viewing less CSAM, *M* = 3.93, *SD* = 2.12, than nonparents, *M* = 4.48, *SD* = 2.10, *t* (659) = −3.28, *p* < 0.001, higher self-transgressions, *M* = 2.03, *SD* = 1.17, than nonparents, *M* = 1.82, *SD* = 1.02, *t* (659) = 2.43, *p*=0.015, and higher positive reappraisal, *M* = 3.80, *SD* = 1.29, than nonparents, *M* = 3.56, *SD* = 1.33, *t* (657) = 2.35, *p*=0.021. No other effects achieved significance. Caregivers of people over 18 versus not differ from noncaregivers on outcome measures. Moreover, none of these findings remained significant in regressions controlling for other demographics.

#### 3.4.2. Risk and Protective Factors

The PCA revealed three factors: contact with victims and perpetrators, exposure to CSAM, and feeling successful and supported. We regressed each dependent variable on all three factors, simultaneously controlling for demographic variables (Table 7). Results indicated that contact with victims and perpetrators uniquely predicted increased depression, anxiety, and PTSD, whereas exposure to CSAM uniquely predicted only moral injury. Moreover, results indicated that feeling successful and supported powerfully predicted all dependent measures: reduced moral injury, depression, anxiety, and PTSD, and increased wellbeing. The effect sizes for this protective factor were more than double the effect size of either risk factor. This points to the powerful importance of feeling successful, supported, and engaged for managing the stresses and challenges of dealing with CSAM.

#### 3.4.3. Moral Injury

We regressed each outcome on transgression-self, transgression-other, and betrayal, controlling for demographics (Table 8). We anticipated largely replicating Bryan et al. [31]. Results instead showed that betrayal was the strongest predictor of increased depression, anxiety, PTSD, as well as reduced wellbeing. Conversely, transgression-self and transgression-other did not predict any outcome except that transgression-self predicted increased PTSD and reduced wellbeing.

Follow-up analyses examined the prediction of each PTSD subscale. Betrayal predicted all six subscales (all *β*'s> 0.17, all *p*'s < 0.001), whereas neither transgression-other (all *β*'s ≤ 0.07, all *p*'s ≥ 0.077) nor transgression-self (all *β*'s ≤ 0.09, all *p*'*s* ≥ 0.044) significantly predicted any subscale except avoidance of reminders, which was predicted by transgression-self, *β* = 0.12, *p*=0.008, transgression-other, *β* = 0.12, *p*=0.003, and betrayal, *β* = 0.17, *p* < 0.001.

#### 3.4.4. CERQ

We treated cognitive and emotion regulation strategies as simultaneous predictors of outcomes controlling for demographic variables (Table 9). We anticipated replicating Garnefski and Kraaij [9] that self-blame, rumination, and catastrophizing would predict increased depression and anxiety, whereas positive reappraisal would predict reduced depression and anxiety. We expected a similar pattern for moral injury and PTSD and the opposite for wellbeing. Results largely corroborated expectations: Self-blame predicted increased depression, moral injury, PTSD, and reduced wellbeing, with a similar but nonsignificant trend for anxiety. Likewise, rumination and catastrophizing predicted all outcomes as predicted. Positive reappraisal predicted reduced depression, as expected, though it failed to predict any other outcome.

We had no predictions for other coping styles. Acceptance failed to predict any outcomes, but positive refocusing predicted reduced depression, anxiety, PTSD, and increased wellbeing (though not moral injury). Planning predicted increased wellbeing, and other-blame predicted increased moral injury, but no other outcome. “Putting into perspective” predicted increased depression and reduced wellbeing, suggesting that strategies like “*I tell myself there are worse things in life*” can backfire.

#### 3.4.5. BERQ

We conducted similar analyses on behavioral emotion regulation strategies (Table 10). We anticipated replicating Kraaij and Garnefski [10] that withdrawal and ignoring would predict increased depression and anxiety, whereas seeking distraction and social support would predict reduced depression and anxiety. We expected a similar pattern for moral injury and PTSD and the opposite pattern for wellbeing. Results largely corroborated expectations. Withdrawal and ignoring predicted increased moral injury, depression, anxiety, PTSD, and reduced wellbeing. Seeking distraction predicted reduced depression, anxiety, and PTSD and increased wellbeing, and social support predicted reduced depression and PTSD and increased wellbeing, though not moral injury or anxiety. Approach increased wellbeing only.

#### 3.4.6. Religious Coping

We predicted that, consistent with other work, positive religious coping would be weakly negatively or unassociated with depression, anxiety, and PTSD but positively associated with wellbeing, whereas negative religious coping would be positively associated with PTSD, anxiety, and depression, but weakly negatively or unassociated with wellbeing [49, 50, 67]. Results partially supported hypotheses (Table 11). As expected, negative religious coping predicted increased depression, anxiety, and PTSD, and positive religious coping failed to predict moral injury, depression, anxiety, and PTSD. However, positive religious coping failed to predict wellbeing; instead, negative religious coping predicted reduced wellbeing. Moreover, these findings controlled for religiosity, which remained a significant predictor of depression and PTSD.

### 3.5. Mediation Analyses

Finally, as preregistered, we conducted mediation analyses to examine how the CERQ, BERQ, and RCOPE (separately) mediated significant variance from moral injury to depression, anxiety, PTSD, and wellbeing (separately), controlling for demographics via PROCESS Macro Model 4 with 10,000 bootstraps to identify indirect effects via any measures that are significant (i.e., the 95% confidence interval [CI] excludes zero, [68]). We anticipated that coping styles significant in regressions would likewise carry significant indirect variance from risk and protective factors and moral injury subscales to outcome measures (i.e., self-blame, rumination, catastrophizing, and positive reappraisal, as well as withdrawal, ignoring, seeking distraction, social support). We also tested whether these patterns held for each facet of moral injury.

#### 3.5.1. Mediation via CERQ

**Figure 1:**
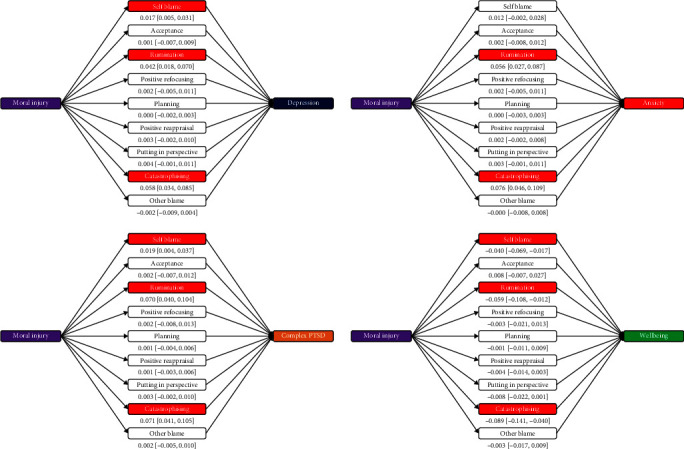
Mediation analysis testing indirect effect of moral injury on depression, generalized anxiety, C-PTSD, and wellbeing via cognitive and emotion coping styles, controlling for demographics. *Note:* Indirect effect point estimates and 95% CIs depicted; colored mediators are significant (95% CI excludes zero): red indicates negative indirect effects, and green indicates positive indirect effects. CI, confidence interval; C-PTSD, complex posttraumatic stress disorder.

First, we examined whether the CERQ mediated significant indirect variance from moral injury to each dependent measure (Figure 1). Rumination and catastrophizing carried significant indirect variance from moral injury to depression, anxiety, PTSD, and wellbeing. In addition, self-blame carried significant indirect variance from moral injury to depression, PTSD, and wellbeing, but not anxiety. These patterns held for each moral injury in each facet, except that self-blame was not always significant, and for wellbeing putting in perspective became significant. Rumination and catastrophizing remained significant mediators in all analyses.

#### 3.5.2. Mediation via BERQ

**Figure 2:**
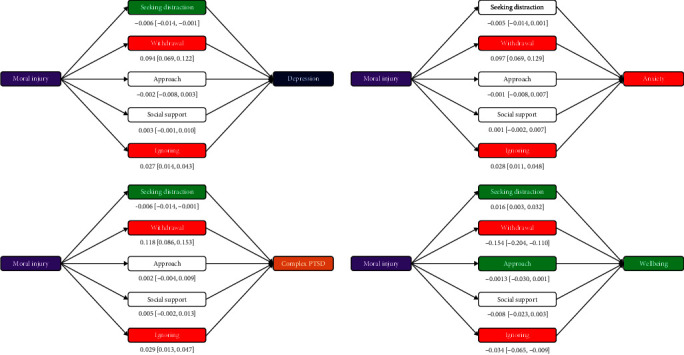
Mediation analysis testing indirect effect of moral injury on depression, generalized anxiety, C-PTSD, and wellbeing via behavioral coping styles, controlling for demographics. *Note:* Indirect effect point estimates and 95% CIs depicted; colored mediators are significant (95% CI excludes zero): red indicates negative indirect effects, and green indicates positive indirect effects. CI, confidence interval; C-PTSD, complex posttraumatic stress disorder.

Next, we conducted a similar analysis on the BERQ (Figure 2). Seeking distraction carried significant indirect variance from moral injury to reduced depression and PTSD and increased wellbeing but not anxiety. Withdrawal and ignoring carried significant indirect variance from moral injury to increased depression, anxiety, PTSD, and reduced wellbeing. The approach also carried significant indirect variance, such that moral injury predicted a reduced approach, which in turn increased wellbeing. However, this effect was small, and it emerged on only one measure, so we interpret it with caution. These patterns held when breaking down moral injury into each facet, except that approach was not a significant mediator of wellbeing for transgression-other and distraction was not a significant mediator of wellbeing for betrayal.

#### 3.5.3. Mediation via Religious Coping

**Figure 3:**
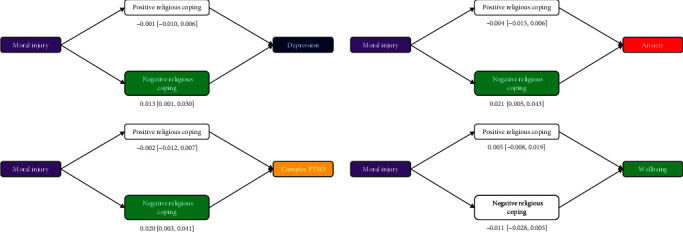
Mediation analysis testing indirect effect of moral injury on depression, generalized anxiety, C-PTSD, and wellbeing via religious coping styles, controlling for demographics. *Note:* Indirect effect point estimates and 95% CIs depicted; colored mediators are significant (95% CI excludes zero): red indicates negative indirect effects, and green indicates positive indirect effects. CI, confidence interval; C-PTSD, complex posttraumatic stress disorder.

Finally, we conducted a similar analysis on religious coping (Figure 3). There were no significant effects via positive religious coping, but negative religious coping carried significant indirect variance from moral injury to depression, anxiety, and PTSD (but not wellbeing). These patterns held when breaking down moral injury into each facet, except that negative religious coping was no longer a significant mediator of depression, anxiety, or PTSD for transgression-other, and negative religious coping also failed to mediate the effect of betrayal on depression.

## 4. Discussion

This project provides a large and comprehensive survey of the wellbeing of UK police officers and staff dealing with CSAM. Results suggest substantially elevated rates of depression and anxiety and somewhat low wellbeing. However, PTSD does not appear elevated. Analyses of predictors indicated that experiencing job success and support powerfully buffered against stressors, predicting reduced depression, anxiety, PTSD, and increased wellbeing. Conversely, moral injury—specifically, feelings of betrayal by colleagues and institutions—powerfully predicted worse mental health outcomes. Although risks like exposure to CSAM predicted increased moral injury and interactions with victims and perpetrators predicted depression, anxiety, and PTSD, the size of these effects was dwarfed by job success and support.

Analyses of coping styles indicated that self-blame, rumination, catastrophizing, withdrawal, ignoring, and negative religious coping predicted worse mental health outcomes. Conversely, positive refocusing, seeking distraction, and social support predicted better outcomes, and there was modest support for positive reappraisal, planning, and approach. Moreover, self-blame, rumination, catastrophizing, withdrawal, ignoring, and negative religious coping mediated significant indirect variance from moral injury to mental health. Importantly, these results emerged above and beyond demographic variables, including age, gender, career length, relationship status, parenting status, religiosity, and therapy experience. Most demographic variables predicted little to no variance in any outcome except religiosity and therapy experience.

Overall, these results suggest that UK police officers and staff investigating CSAM experience elevated depression and anxiety and reduced wellbeing—but the most powerful thing colleagues and institutions can do to improve outcomes is to help officers and staff feel successful and supported rather than undermined. Colleagues and leaders should stay vigilant for signs of distress, including rumination, catastrophizing, and withdrawal, and aim to foster mindsets like positive refocusing and opportunities for social support.

### 4.1. Comparing Clinical Symptoms and Coping Across Samples

The ratings of clinical symptoms help clarify the mental health of CSAM investigators compared to other police officers. Comparisons are often challenging as different studies use different measures, samples, or cutoffs. Nonetheless, some comparisons are possible, such as Stevelink et al. [63], who measured depression, anxiety, and PTSD in ~40,000 UK police, including a subset of ~5000 people who reported a traumatic event within the past 6 months. Carleton et al. [69] measured depression using the PHQ-9 and anxiety using the GAD-7 in a sample of ~6000 Canadian safety personnel, including national and provincial police branches. Brewin et al. [70] measured PTSD and C-PTSD using the International Trauma Questionnaire in ~9000 UK police who reported exposure to trauma.

#### 4.1.1. Depression

The current sample demonstrated much higher rates of depression (27.7%) in CSAM investigators than Stevelink et al. [63] findings of 9.8% in UK police overall and 17.8% among police with recent trauma. Rates were also substantially higher than the 19.6% Carleton et al. [69] reported in municipal/provincial Canadian police, albeit lower than the 31.7% they reported in national police (who are frequently deployed to rural areas and often work alone, hence lack access to support). Hence, rates of depression among CSAM investigators may be substantially higher than among typical police—even those exposed to trauma—with rare exceptions. Moreover, the current figures are alarmingly higher than figures for the broader UK population. Smith et al. [71] examined 172,751 middle-aged UK residents and found a prevalence of 1.5% probably major depression (on the related PHQ7) among people with no mental health history (*N* = 98,539), 7.1% among people with recurrent moderate major depression (*N* = 15,013), and 12.2% among people with a history of bipolar disorder (*N* = 1615). Hence, CSAM investigators exhibit depressive symptoms at substantially higher rates than UK police and civilians with and without mental health struggles. 3

#### 4.1.2. Anxiety

Likewise, CSAM investigators demonstrated generalized anxiety rates (24.2%) much higher than 8.5% in UK police and 16.4% among police with recent trauma [63], though results should be interpreted with caution as they used a different measure of anxiety). The current sample also demonstrated higher anxiety than the 14.6% for municipal/provincial Canadian police [69], though comparable to the 23.3% they reported for national police. Global estimates of GAD range from 3.8% to 25% [72]. GAD typically varies between 2% and 10% in Western populations [72]. A recent study of 30,446 UK residents found only 2.2% qualified for GAD within the past year [73]. Even higher rates emerge for people with medical problems, such as 10.94% [74], pale in comparison to 24.2% in the current sample. Hence, anxiety rates appear substantially elevated in the current sample.

#### 4.1.3. PTSD

Unlike depression and anxiety, PTSD rates in the current sample compare favorably with other police branches. Among CSAM investigators, we found a PTSD rate of 8.7% (including 3.3% PTSD and 5.4% C-PTSD)—notably lower than 20.6% (8.0% PTSD, 12.6% C-PTSD) in trauma-exposed UK police [70] and 19.5% for Canadian municipal/provincial police and 30.0% for national police [69]. Granted, the current rates are comparable to ~3%–5% PTSD and ~6%–8% C-PTSD in 2444 UK police [75], and higher than 3.9% Stevelink et al. [63] reported for overall UK police—using a different measure—but lower than the 27% they reported for trauma-exposed police.

That said, PTSD rates for CSAM investigators in the current sample are higher than global estimates: a meta-analysis found an average of 4.7% PTSD among police [76], estimates of 3%–6% PTSD among UK veterans of war [77]. However, the current work numbers are notably lower than civilian samples, including 18.3% in a UK representative sample [78] and 14.6% in our online UK comparison sample Supporting Information 2: File S2. Therefore, although rates of PTSD are perhaps higher than ideal among CSAM investigators, they show rates often comparable to or lower than other branches of the force and society, albeit higher than some samples. Therefore, PTSD management may not be a priority for increasing wellbeing among this population compared to depression and anxiety.

#### 4.1.4. Coping Strategies

Previous research on coping strategies for police and CSAM investigators is limited. Several papers have documented that negative coping strategies are associated with worse mental health among CSAM investigators [39], such as denial, withdrawing from family and friends [46], and use of alcohol and tobacco [5]. Knowles and Bull [43] found that negative coping strategies like avoidance and disengagement predicted psychological and physiological stress symptoms, but positive coping strategies like approach and engagement had no effect for general police officers. Brady [19] found a marginally significant effect for what they called ‘positive coping strategies' like getting sleep or taking breaks, but these strategies do not align with the more comprehensive and theory-based cognitive, emotional, and behavioral coping strategies examined here and the evidence is weak.

The current work confirms that negative strategies like avoidance and disengagement predicted reduced mental health outcomes but also found improved support for the argument that some approaches and engagement strategies, like seeking social support and positive refocusing, seemed effective. Moreover, some avoidance strategies like seeking distraction appeared surprisingly effective at improving mental health outcomes—which may be important, considering that distraction is the most common coping mechanism reported by CSAM investigators [41, 42]. Distraction may help CSAM investigators temporarily avoid processing this traumatic information until later when they have opportunities for social support or dispassionate reflection. However, some work has found the opposite, that distraction predicts greater trauma and burnout [79]. It is possible that such work combines reports of distraction and ignoring, which had opposite effects in our data.

The BERQ findings reveal a distinction between the healthy tendency to distract oneself, as reflected by the item *I set my worries aside by doing something else*, versus the unhealthy tendency to ignore problems, as reflected by the item, *I* *repress it and pretend it never happened*. It may be that people describing distraction view it as a temporary strategy that allows them to pause processing upsetting material until later when perhaps they can be more reflective or gain social support, whereas people describing ignoring view it as a permanent strategy of continuously avoiding the topic despite the impact on their lives. Further research should clarify when and why distraction may be helpful versus harmful.

### 4.2. Implications

These results have implications for several areas of inquiry.

#### 4.2.1. Mental Health of CSAM Investigators

These results add to a growing body of work examining the mental health and wellbeing of police and other civil servants. They align with past findings suggesting that organizational culture and lack of social support are major sources of stress, mental health problems, and motivation for leaving the force [80–83], whereas support from peers and supervisors is effective at increasing police wellbeing [19–21]. Moreover, the current findings (see Supporting Information 4: File S4) align with work showing that law enforcement personnel are often unlikely to seek treatment for mental health issues due to factors like stigma, potential career impacts, and concerns over their image [6, 84, 85].

Research directly examining people exposed to CSAM is limited, with most studies examining stress, trauma, or burnout. Current results largely align with past work by documenting elevated rates of mental health difficulties [8, 15–17]. For example, in one study, over half of UK child protection detectives reported at least one trauma symptom [13]. We extend such findings to moral injury, anxiety, depression, PTSD, and wellbeing. The current results also align with studies demonstrating that organizational support and job success buffer mental health [5, 8, 19, 22, 46]. Although Bourke and Craun found this effect among only American—not UK—participants, we found the effect in the United Kingdom. Again, such findings extended beyond trauma and burnout.

We found no relationship between the length of participants' careers or current role and mental health. Such findings do not align with Perez et al. [8], who found that the longer people worked with CSAM, the higher they scored on burnout and cynicism, and therefore recommended limiting the amount of time investigators spent dealing with CSAM. Likewise, Losung et al. [44] found that longer tenure working with CSAM predicted burnout. Our findings provide no evidence that limiting the length of CSAM careers is helpful so long as investigators receive adequate support.

Likewise, there was little evidence that exposure to CSAM itself is the primary problem faced by investigators—exposure to CSAM did not predict depression, anxiety, PTSD, or wellbeing, though it did predict moral injury. Such findings align with qualitative research suggesting that CSAM investigators rarely spontaneously mention exposure to CSAM as a primary stressor in their job; instead referring to organizational factors like workload demands [15, 24, 86]. It may be that exposure to CSAM did not predict much because this issue has already received considerable attention. Hence, results reinforce the importance of investment in digital forensic tools that employ image classifying technology to identify CSAM at scale—meaning investigators spend less time reviewing and categorizing files, thereby protecting their mental wellbeing and allowing them to focus on identifying victims and advancing their investigations [87].

#### 4.2.2. Moral Injury

These results have several implications for the study of moral injury. First, consistent with Bryan et al. [31], we found evidence for three separate factors underlying moral injury: transgression-self, transgression-other, and betrayal by colleagues and institutions. This finding is important because Nash et al. [40] found factor analytic evidence for two components of the MIES: perceived transgression by self or others and betrayal. Bryan et al. [31] instead found evidence for a three-factor solution. However, recently, Richardson et al. [88] did not corroborate the three-factor solution, instead finding evidence for two different components: moral injury “self” and “other” (see also [35]). The current work corroborated the three-factor solution for the police sample. However, intriguingly, the online comparison sample instead found evidence for the two-factor “self” and “other” solution Supporting Information 2: File S2. Therefore, whether one finds a two- or three-factor solution may depend in part on the sample in question and the kind of moral infractions they consider. For example, it may make sense for the general public to conceive of transgression-others and betrayal as related concepts as the people they think of as transgressing (e.g., friends, partners) may also be those betraying them. Conversely, police officers and staff may think of one set of targets as primarily transgression—CSAM perpetrators—whereas a different set of targets as betraying—their colleagues and intuitions. Researchers should consider this possibility when assessing moral injury in the future.

Second, consistent with a large body of work [33, 49, 50, 89], we found that moral injury predicted a range of worse mental health outcomes. Yet, results in the current work differed from past work in important ways. Most research on moral injury has examined military veterans [49]; relatively few papers have examined moral injury in police officers or staff (see [3]). Studies of military personnel using the same three-facet breakdown of moral injury as the current work have demonstrated that transgression-other—witnessing others commit moral infractions—was an important predictor of mental health outcomes, in addition to betrayal—feeling betrayed by colleagues and institutions [31]. Other work highlights the negative impact of transgression-self on mental health, in addition to betrayal [32, 89]. Likewise, Papazoglou et al. [35] assessed moral injury and PTSD in a sample of Finnish police. They divided moral injury into “self” and “other” components (i.e., amalgamating transgression-other and betrayal). They found evidence for higher associations between self-focused than other-focused moral injury with PTSD. Thus, transgression-other and -self appear as important predictors in military and police samples, with betrayal playing a lesser role.

Conversely, in the current work, betrayal was by far the most important predictor of mental health, with transgression-other failing to reach significance for any mental health measure. Although transgression-self—one's own moral failings—predicted increased PTSD and reduced wellbeing, consistent with Currier et al. [32], this effect was much smaller than the effect of betrayal on PTSD. Hence, whereas Currier et al. [32] and Papazoglou et al. [35] find that transgression-self best predicts PTSD in military and general police samples, we find only a small effect of this factor and instead a large effect of betrayal. It seems that for CSAM investigators, feelings of moral betrayal by colleagues were by far the most important factor driving outcomes. This pattern is especially striking, considering that participants deal directly with morally compromising information and individuals, risking the potential for both transgression-self and others to matter.

It is possible this different pattern reflects different populations: it may be that military and general police are more frequently in a position to commit violence or ethical infractions (e.g., during patrol), leading to a higher impact of self-transgressions. Conversely, much CSAM investigation may take place in officers or institutions with less opportunity for officers and staff to directly commit violence or other infractions, so transgression-self impacts their outcomes less than military personnel or other branches of the police. A key implication of this pattern is that there is a large role for colleagues and institutions to play to manage how police officers and staff feel regarding moral injury. Hence, this pattern is mirrored by the large buffering effect of job success and support on wellbeing—it seems that when colleagues and organizations support individuals, things tend to go well, but when they undermine individuals, things go poorly. This impact seems to matter more than any other factor we assessed.

We also examined how well Bryan et al.' results would replicate regarding the impact of moral injury on the facets of PTSD. Results differed substantially. They found that transgression-other predicted the reexperiencing, numbing, hyperarousal, and avoidance subscales, betrayal predicted reexperiencing and numbing (affect dysregulation), and transgression-self predicted affect dysregulation. However, in the current work, only betrayal predicted all six PTSD facets—transgression-self and transgression-other failed to predict any facet. The only exception to this pattern was that all three subscales (betrayal, transgression-other, transgression-self) predicted avoidance of reminders of the morally injurious event. Papazoglou et al. [36] found that moral injury predicted increases in each of three facets of PTSD (reexperiencing, hyperarousal, and avoidance). However, they did not divide moral injury into components in this paper. As the sample may be the same as their 2019 paper, it may be that transgression-self predicted most PTSD facets. If so, then the results differ substantially from the current findings.

Together, these results suggest that CSAM investigators may experience moral injury in a different way than soldiers returning from the battlefield or general police duties. Such findings raise questions about the generalizability of past moral injury findings. Like questions over the factor structure, it may be that different moral concerns related to different duties help explain the disparity. Future research might profitably examine this question by exploring the core moral concerns of people with (for example) different police duties or military duties.

Finally, the mediation results suggest possible psychological mechanisms underlying the impact of moral injury on mental health. The CERQ and BERQ mediations suggest that moral injury impacts mental health via rumination, catastrophizing, self-blame, withdrawal, and ignoring. Conversely, seeking distraction appears to buffer some of the effects of moral injury on mental health. Hence, moral injury is associated with particularly toxic coping styles focused on the moral shortcomings of the self, which helps explain why it may be so toxic. Moreover, the COPE mediation suggests that moral injury is associated with questioning whether one is suffering due to divine punishment—particularly true when people contemplate their own moral transgressions as opposed to someone else's. Therefore, concern over religious punishment for misdeeds may be a key psychological mechanism involved in moral injury, partially explaining the negative effect of this variable on mental health.

#### 4.2.3. Coping Styles

This work also has broader implications for the study of cognitive, emotional, and behavioral coping styles. We largely replicated Garnefski and Kraaij [9, 10]. Specifically, we replicated the findings that self-blame, rumination, and catastrophizing predicted worse mental health outcomes, whereas positive reappraisal predicted reduced depression (though positive reappraisal failed to predict any other measure). Likewise, we replicated the findings that withdrawal and ignoring predicted worse mental health outcomes, whereas seeking distraction and social support predicted better outcomes. These robust patterns across studies demonstrate the importance of these coping styles and increase generalizability. We also found some evidence for the utility of positive refocusing, planning, and approach, and potentially negative impacts of other-blame and putting into perspective.

Moreover, the current work goes beyond past research to examine how each set of coping styles mediates the impact of moral injury on mental health outcomes. These results suggest that moral injury primarily influences mental health outcomes via self-blame, rumination, catastrophizing, ignoring, and withdrawal. Conversely, seeking distraction appears to mitigate the influence of moral injury on mental health.

These findings may suggest intervention strategies useful for assisting police officers and staff deal with CSAM, as well as strategies to avoid. The most promising approaches appear to be social support and seeking distraction. Consistent with this possibility, some participants wrote about their desire for more social events with colleagues where they could connect on a personal level and find solace in shared experiences [23]). In addition, positive reappraisal may be useful to some extent. Conversely, the results of the “putting into perspective” analysis suggest that attempts to make oneself feel better through downward social comparisons (at least I do not have things as bad as that person) may backfire. In addition, results reveal a distinction between the healthy tendency to distract oneself versus the unhealthy tendency to ignore the problem. Distraction may serve as a temporary strategy that allows people to pause processing upsetting material until later when perhaps they can be more reflective or gain social support. Conversely, ignoring may work as a permanent strategy of continuously avoiding the topic. It may be worth clarifying how such similar strategies produce such different results.

Finally, self-blame, rumination, catastrophizing, and withdrawal share conceptual elements with guilt and especially shame [90]. Although guilt and shame reflect somewhat overlapping psychological constructs, as both reflect moral concerns about transgressions and suggest a tarnished but earnest concern for others [91, 92], they can have different effects. Guilt promotes active reparations to restore the moral self-image, whereas shame promotes withdrawal to avoid further damage to the moral self [93, 94]. Thus, when moral injury leads to feelings of shame, people may engage in more self-blame, rumination, and withdrawal, leading to mental health problems. It may be that one effective intervention technique, therefore, is to help people cognitively reframe their shame as guilt instead, paving the way for the restoration of the moral self-image. Future work might explore this possibility.

#### 4.2.4. Religiosity and Religious Coping

One notable element in the current work is the finding that religiosity in this sample predicted higher rates of depression, anxiety, and PTSD. This finding flies in the face of most work on this topic, which typically finds that religiosity buffers the impact of life stressors on mental health outcomes ([95, 96]; for a review, see [97]). However, this result does align with some findings—Bonelli et al. noted several studies where religiosity is associated with worse outcomes. For example, religiosity predicted worse outcomes for family problems related to children and abuse [98]. These domains align with the content faced by CSAM investigators: abuse of children. Conceptually, therefore, it makes sense that religiosity predicted worse outcomes in this sample. Ellison [99] argued that certain stressors directly conflict with religious values. CSAM may raise questions of faith for religious people, leading people to wonder how a loving deity could allow the harm to innocents they witness. They may also experience dissonance moving between their work environment and faith community and struggle to reconcile these very different worlds [39]. A similar dynamic is found in the challenges faced by Reaper drone crews, who mentally transition each day between their screen-mediated military operations, where they witness the horrors of war and peaceful domestic life with family, friends, and neighbors [100]. Consistent with this argument, religiosity did not predict any outcome in the online comparison sample except moral injury, despite similar rates of religious endorsement. Indeed, moral injury may provide a special challenge to religious values [48].

On a related note, we also found that negative religious coping—e.g., questioning whether one is being punished by one's deity—predicted worse mental health outcomes above and beyond religiosity. Conversely, positive religious coping—i.e., turning to faith for solace—did not significantly predict outcomes. This finding was expected, as it matches most findings in this area. For example, Currier et al. [49] found that negative but not positive religious coping predicted risk for suicide among veterans, and Pargament et al. [11] found that negative but not positive religious coping predicted depression and reduced quality of life (see also [19]). The current results mesh with these findings but also extend them by showing that negative religious coping mediated the impact of moral injury on mental health outcomes. This pattern suggests that people who experience moral injury may demonstrate worse mental health in part because they belief that God is punishing them.

#### 4.2.5. Gender and Other Demographic Predictors of Mental Health

One striking result in the current work is that demographic factors contributed little in the way of variance to mental health outcomes, despite the fact our sample was large enough to sensitively detect small effects. Past work has often demonstrated demographic differences in mental health. For example, younger people often report worse mental health than older people [101], and people in relationships often report better mental health outcomes than single people [102]. In addition, women often report worse outcomes than men [103], including in police samples [80] and CSAM investigators [5, 104], though a few studies have found men at greater risk [41, 44]. Finally, in an American sample of CSAM investigators, nonwhite investigators reported better mental health than White investigators [22]. Yet, none of these patterns emerged in the current sample—not even in the correlational analysis without multicollinearity concerns. 4 These findings challenge received wisdom over the importance of such demographic factors for predicting outcomes, especially considering that theoretically relevant predictors were powerfully effective.

Moreover, we considered it theoretically plausible that parents of young children would report worse outcomes than people who were not parents, perhaps due to dissonance between exposure to CSAM in the workplace versus caring for children at home. It seemed plausible that such tensions might exacerbate the effect of CSAM investigation on mental health outcomes—yet we find no evidence of this effect. Likewise, we considered it theoretically plausible that length of service or time spent in CSAM investigation roles would predict worse outcomes, perhaps as repeated exposure to CSAM over years would wear down defenses and increasing cause difficulty. Yet, there appears to be no evidence for this possibility—senior people appear as well-adjusted as junior people, so long as they report comparable levels of job success and support and engage in comparable coping strategies. This point may be important when considering factors like promotions or reassignment. The current work suggests no upper limit on the recommended time a person remains in CSAM investigation roles so long as they have sufficient support.

The only demographic factors to predict outcomes were religiosity (considered below) and experience with therapy. Unfortunately, our measure of therapy experience was imprecise; we simply asked people to mention whether they currently were or had ever engaged in therapy. Therefore, we are unable to disentangle whether the therapy people report is related to their CSAM investigation role or not and whether the therapy is current or in the past. Moreover, the data is cross-sectional rather than longitudinal, so we cannot draw inferences as to whether therapy improves mental health in these data. It may be that people who report worse mental health then seek therapy or that people who have ever visited therapy report worse mental health or some combination thereof. Both findings make sense: people experiencing distress should seek professional assistance, and people who experienced distress in the past (therefore seeking therapy) may continue to struggle in the present. Either way, the current results should not be taken as evidence that therapy is ineffective in this population.

#### 4.2.6. Response to Calls for Research

This project responds to several calls for research. Papazoglou and Chopko [3] called for research on moral injury among police, particularly work on how moral injury relates to PTSD and coping styles. The current work provides a large police sample examining moral injury, PTSD, and coping styles. Frankfurt and Frazier [12] argued for (a) researching risk and protective factors, (b) examining factors beyond PTSD, such as spirituality, and (c) testing more complex models, such as mediation. The current work does exactly this, examining risk and protective factors, mental health beyond PTSD, spirituality, and mediation models.

## 5. Limitations

Like all research, this work suffers from limitations. First, this survey suffers from self-selection and hence may not be representative of all officers and staff investigating CSAM. Although the questionnaire was distributed by the UK National Police Chiefs' Council, multiple reminders were sent, and participation was encouraged by gift cards, it nonetheless may be that people with specific tendencies—for example, those feeling especially distressed—were more likely to take part. Hence, generalization should be done with caution.

Second, we asked about symptoms of depression and anxiety over the past month rather than the past 2 weeks, as is common. This aligned the PHQ and GAD with the International Trauma Questionnaire, simplifying participation. Yet, this change could potentially inflate or reduce scores relative to other samples. High scores specify feeling this way for, e.g., “more than half of the days” during the specified period of time, so hence extending the timeframe may reduce scores by increasing the number of days participants do not feel symptoms. Conversely, participants may recall additional days of symptoms. Therefore, caution should be exercised when comparing with other samples.

Third, these data are correlational and cannot reveal causality. Recent longitudinal data [32] showed that moral injury at Time 1 predicted increased PTSD symptoms 6 months later, suggesting that moral injury may partially cause mental health outcomes. However, the same data revealed that PTSD at Time 1 also predicted subsequent moral injury, suggesting possible reciprocal influence. The current data cannot speak to these possibilities; nor can they reveal trends over time. Moreover, the survey occurred toward the tail end of the COVID-19 pandemic, raising questions regarding generalizability that future work can clarify.

Fourth, we recognize the need for further qualitative research to provide a richer and more nuanced understanding of the subjective experience of the cohort involved [23]. Accordingly, we are currently developing qualitative research with personnel who view CSAM as part of their role with a view to gaining insight into how they make sense of, and understand, their experience and the context within which they work in relation to their own mental health and wellbeing.

## 6. Conclusion

The current work provides a large and comprehensive examination of the mental health of UK police officers and staff dealing with CSAE. Results suggest that dealing with such content can elevate anxiety and depression, but the way colleagues and institutions treat CSAM investigators can make a large impact. These results may serve as a call to action among police departments to foster improved wellbeing and cultivate a culture that prioritizes mental health.

## Figures and Tables

**Table 1:** UK region participants reported working in.

Region	*N*	Percent
Southeast	93	14.1
Southwest	60	9.1
London	47	7.1
East Midlands	49	7.4
West Midlands	53	8.0
Eastern	52	7.9
Northeast	73	11.0
Northwest	76	11.5
Wales	16	2.4
Scotland	43	6.5
Northern Ireland	28	4.2
Yorkshire	40	6.1
Other (e.g., National)	29	4.4
Unreported	2	0.3
Total	661	100.0

**Table 2:** Roles participants reported.

Role	*N*	Percent
Child protection unit	236	35.7
Investigations	167	25.3
Other (e.g., call handler, covert, forensics, school liaison officer)	129	19.5
POLIT	126	19.1
Intelligence	39	5.9
Digital forensics	30	4.5
MOSOVO	29	4.4
Operational support	25	3.8
Administrative support	13	2.0
Criminal justice	15	2.3
Neighborhood policing	6	0.9
National policing	5	0.8
Response policing	4	0.6
Training	4	0.6
Total	828	125.4

*Note:* Responses sum to >100% because participants selected all that apply.

Abbreviations: MOSOVO, management of sexual or violent offenders; POLIT, police online investigation team.

**Table 3:** Participants scoring in each clinical range on the PHQ-9.

Severity	*N*	Percent
Minimal	245	37.1
Mild	233	35.2
Moderate	108	16.3
Moderately severe	50	7.6
Severe	25	3.8

Abbreviation: PHQ-9, patient health questionnaire-9.

**Table 4:** Participants scoring in each clinical range on the GAD-7.

Severity	*N*	Percent
Minimal	279	42.2
Mild	222	33.6
Moderate	105	15.9
Severe	55	8.3

Abbreviation: GAD-7, generalized anxiety disorder-7.

**Table 5:** Participants scoring in the clinical range on PTSD and C-PTSD.

Diagnosis	*N*	Percent
Not clinical	603	91.2
PTSD	22	3.3
C-PTSD	36	5.4

Abbreviations: C-PTSD, complex posttraumatic stress disorder; PTSD, posttraumatic stress disorder.

**Table 6a:** Correlations between all measures in the study.

Variable	1	2	3	4	5	6	7	8	9	10	11	12	13	14	15	16	17	18	19	20	21
1. Depression	—	—	—	—	—	—	—	—	—	—	—	—	—	—	—	—	—	—	—	—	—
2. Anxiety	**0.82**⁣^*∗∗*^	—	—	—	—	—	—	—	—	—	—	—	—	—	—	—	—	—	—	—	—
3. C-PTSD	**0.82**⁣^*∗∗*^	**0.77**⁣^*∗∗*^	—	—	—	—	—	—	—	—	—	—	—	—	—	—	—	—	—	—	—
4. PTSD	**0.72**⁣^*∗∗*^	**0.71**⁣^*∗∗*^	**0.92**⁣^*∗∗*^	—	—	—	—	—	—	—	—	—	—	—	—	—	—	—	—	—	—
5. Disturbance of self	**0.78**⁣^*∗∗*^	**0.71**⁣^*∗∗*^	**0.94**⁣^*∗∗*^	**0.72**⁣^*∗∗*^	—	—	—	—	—	—	—	—	—	—	—	—	—	—	—	—	—
6. Wellbeing	**−0.71**⁣^*∗∗*^	**−0.67**⁣^*∗∗*^	**−0.71**⁣^*∗∗*^	**−0.55**⁣^*∗∗*^	**−0.75**⁣^*∗∗*^	**—**	—	—	—	—	—	—	—	—	—	—	—	—	—	—	—
7. Exposure to CSAM	−0.02	−0.03	0.04	0.04	0.03	−0.02	—	—	—	—	—	—	—	—	—	—	—	—	—	—	—
8. Contact with victims and perpetrators	**0.19**⁣^*∗∗*^	**0.17**⁣^*∗∗*^	**0.15**⁣^*∗∗*^	**0.17**⁣^*∗∗*^	**0.12**⁣^*∗∗*^	−0.06	**−0.14**⁣^*∗∗*^	—	—	—	—	—	—	—	—	—	—	—	—	—	—
9. Job success and support	**−0.45**⁣^*∗∗*^	**−0.40**⁣^*∗∗*^	**−0.51**⁣^*∗∗*^	**−0.43**⁣^*∗∗*^	**−0.51**⁣^*∗∗*^	**0.53**⁣^*∗∗*^	0.08	**−0.15**⁣^*∗∗*^	—	—	—	—	—	—	—	—	—	—	—	—	—
10. Transgress self	**0.13**⁣^*∗∗*^	**0.16**⁣^*∗∗*^	**0.20**⁣^*∗∗*^	**0.21**⁣^*∗∗*^	**0.16**⁣^*∗∗*^	**−0.15**⁣^*∗∗*^	**0.23**⁣^*∗∗*^	0.01	**−0.13**⁣^*∗∗*^	—	—	—	—	—	—	—	—	—	—	—	—
11. Transgress other	**0.20**⁣^*∗∗*^	**0.17**⁣^*∗∗*^	**0.28**⁣^*∗∗*^	**0.28**⁣^*∗∗*^	**0.25**⁣^*∗∗*^	**−0.25**⁣^*∗∗*^	−0.04	**0.12**⁣^*∗∗*^	**−0.28**⁣^*∗∗*^	**0.29**⁣^*∗∗*^	—	—	—	—	—	—	—	—	—	—	—
12. Betrayal	**0.37**⁣^*∗∗*^	**0.34**⁣^*∗∗*^	**0.41**⁣^*∗∗*^	**0.37**⁣^*∗∗*^	**0.39**⁣^*∗∗*^	**−0.36**⁣^*∗∗*^	0.06	**0.10**⁣^*∗∗*^	**−0.36**⁣^*∗∗*^	**0.30**⁣^*∗∗*^	**0.48**⁣^*∗∗*^	—	—	—	—	—	—	—	—	—	—
13. Self-blame	**0.29**⁣^*∗∗*^	**0.25**⁣^*∗∗*^	**0.30**⁣^*∗∗*^	**0.26**⁣^*∗∗*^	**0.30**⁣^*∗∗*^	**−0.29**⁣^*∗∗*^	**−0.11**⁣^*∗∗*^	**0.15**⁣^*∗∗*^	**−0.21**⁣^*∗∗*^	0.00	**0.28**⁣^*∗∗*^	**0.25**⁣^*∗∗*^	—	—	—	—	—	—	—	—	—
14. Acceptance	**0.11**⁣^*∗∗*^	**0.13**⁣^*∗∗*^	**0.15**⁣^*∗∗*^	**0.13**⁣^*∗∗*^	**0.14**⁣^*∗∗*^	−0.06	**0.12**⁣^*∗∗*^	0.00	−0.07	**0.16**⁣^*∗∗*^	**0.09**⁣^*∗*^	**0.13**⁣^*∗∗*^	**0.12**⁣^*∗∗*^	—	—	—	—	—	—	—	—
15. Rumination	**0.46**⁣^*∗∗*^	**0.47**⁣^*∗∗*^	**0.53**⁣^*∗∗*^	**0.56**⁣^*∗∗*^	**0.43**⁣^*∗∗*^	**−0.39**⁣^*∗∗*^	0.08	**0.20**⁣^*∗∗*^	**−0.27**⁣^*∗∗*^	**0.32**⁣^*∗∗*^	**0.29**⁣^*∗∗*^	**0.35**⁣^*∗∗*^	**0.30**⁣^*∗∗*^	**0.26**⁣^*∗∗*^	—	—	—	—	—	—	—
16. Positive refocusing	**−0.13**⁣^*∗∗*^	−0.07	**−0.13**⁣^*∗∗*^	**−0.09**⁣^*∗*^	**−0.15**⁣^*∗∗*^	**0.16**⁣^*∗∗*^	0.02	−0.03	**0.25**⁣^*∗∗*^	0.06	0.00	−0.06	0.05	**0.16**⁣^*∗∗*^	**0.11**⁣^*∗∗*^	—	—	—	—	—	—
17. Planning	0.01	0.03	−0.01	0.04	−0.05	0.08	0.01	**0.16**⁣^*∗∗*^	**0.19**⁣^*∗∗*^	0.07	−0.04	−0.01	0.00	**0.15**⁣^*∗∗*^	**0.17**⁣^*∗∗*^	**0.15**⁣^*∗∗*^	—	—	—	—	—
18. Positive reappraisal	**−0.16**⁣^*∗∗*^	**−0.09**⁣^*∗*^	**−0.09**⁣^*∗*^	−0.07	**−0.10**⁣^*∗*^	**0.12**⁣^*∗∗*^	0.00	0.05	**0.21**⁣^*∗∗*^	−0.08	−0.01	−0.01	0.07	**0.16**⁣^*∗∗*^	0.00	**0.32**⁣^*∗∗*^	**0.27**⁣^*∗∗*^	—	—	—	—
19. Putting into perspective	0.06	0.06	0.04	0.02	0.05	−0.03	−0.05	**0.13**⁣^*∗∗*^	0.01	0.07	**0.11**⁣^*∗∗*^	0.07	**0.25**⁣^*∗∗*^	**0.24**⁣^*∗∗*^	**0.11**⁣^*∗∗*^	**0.29**⁣^*∗∗*^	**0.10**⁣^*∗∗*^	**0.41**⁣^*∗∗*^	—	—	—
20. Catastrophizing	**0.47**⁣^*∗∗*^	**0.49**⁣^*∗∗*^	**0.52**⁣^*∗∗*^	**0.53**⁣^*∗∗*^	**0.44**⁣^*∗∗*^	**−0.41**⁣^*∗∗*^	0.08	**0.14**⁣^*∗∗*^	**−0.28**⁣^*∗∗*^	**0.29**⁣^*∗∗*^	**0.24**⁣^*∗∗*^	**0.34**⁣^*∗∗*^	**0.28**⁣^*∗∗*^	**0.24**⁣^*∗∗*^	**0.70**⁣^*∗∗*^	0.07	**0.12**⁣^*∗∗*^	−0.08	0.00	—	—
21. Other-blame	0.00	0.03	0.06	0.05	0.05	−0.01	**0.12**⁣^*∗∗*^	−0.01	0.01	**0.31**⁣^*∗∗*^	0.01	0.08	**−0.19**⁣^*∗∗*^	**0.24**⁣^*∗∗*^	**0.11**⁣^*∗∗*^	0.08	**0.17**⁣^*∗∗*^	**0.09**⁣^*∗*^	0.01	**0.09**⁣^*∗*^	—
22. Seeking distraction	0.02	0.05	0.04	**0.10**⁣^*∗∗*^	−0.01	0.06	**0.15**⁣^*∗∗*^	−0.02	**0.10**⁣^*∗∗*^	**0.10**⁣^*∗*^	**0.10**⁣^*∗∗*^	0.01	0.06	**0.16**⁣^*∗∗*^	**0.13**⁣^*∗∗*^	**0.42**⁣^*∗∗*^	**0.11**⁣^*∗∗*^	**0.15**⁣^*∗∗*^	**0.21**⁣^*∗∗*^	**0.13**⁣^*∗∗*^	0.03
23. Withdrawal	**0.60**⁣^*∗∗*^	**0.56**⁣^*∗∗*^	**0.64**⁣^*∗∗*^	**0.56**⁣^*∗∗*^	**0.62**⁣^*∗∗*^	**−0.55**⁣^*∗∗*^	0.05	**0.16**⁣^*∗∗*^	**−0.39**⁣^*∗∗*^	**0.22**⁣^*∗∗*^	**0.25**⁣^*∗∗*^	**0.32**⁣^*∗∗*^	**0.32**⁣^*∗∗*^	**0.15**⁣^*∗∗*^	**0.50**⁣^*∗∗*^	−0.01	0.01	−0.08	**0.13**⁣^*∗∗*^	**0.48**⁣^*∗∗*^	0.01
24. Approach	**−0.17**⁣^*∗*^	**−0.15**⁣^*∗∗*^	**−0.22**⁣^*∗∗*^	**−0.17**⁣^*∗∗*^	**−0.24**⁣^*∗∗*^	**0.28**⁣^*∗∗*^	0.00	**−0.09**⁣^*∗*^	**0.31**⁣^*∗∗*^	−0.02	**−0.11**⁣^*∗∗*^	**−0.16**⁣^*∗∗*^	**−0.13**⁣^*∗∗*^	**0.12**⁣^*∗∗*^	**−0.10**⁣^*∗*^	**0.23**⁣^*∗∗*^	**0.32**⁣^*∗∗*^	**0.12**⁣^*∗∗*^	0.03	**−0.15**⁣^*∗∗*^	0.08
25. Social support	**−0.21**⁣^*∗∗*^	**−0.13**⁣^*∗∗*^	**−0.24**⁣^*∗∗*^	**−0.15**⁣^*∗∗*^	**−0.29**⁣^*∗∗*^	**0.29**⁣^*∗∗*^	−0.07	**0.09**⁣^*∗*^	**0.38**⁣^*∗∗*^	0.00	−0.04	−0.03	0.03	−0.07	0.03	**0.30**⁣^*∗∗*^	**0.17**⁣^*∗∗*^	**0.16**⁣^*∗∗*^	**0.09**⁣^*∗*^	−0.08	0.03
26. Ignoring	**0.43**⁣^*∗∗*^	**0.37**⁣^*∗∗*^	**0.46**⁣^*∗∗*^	**0.40**⁣^*∗∗*^	**0.45**⁣^*∗∗*^	**−0.38**⁣^*∗∗*^	**0.17**⁣^*∗∗*^	**0.10**⁣^*∗∗*^	**−0.31**⁣^*∗∗*^	**0.17**⁣^*∗∗*^	**0.19**⁣^*∗∗*^	**0.26**⁣^*∗∗*^	**0.20**⁣^*∗∗*^	**0.21**⁣^*∗∗*^	**0.29**⁣^*∗∗*^	0.05	−0.01	−0.06	**0.15**⁣^*∗∗*^	**0.35**⁣^*∗∗*^	0.08
27. Positive religious coping	**0.14**⁣^*∗∗*^	**0.10**⁣^*∗∗*^	**0.14**⁣^*∗∗*^	**0.16**⁣^*∗∗*^	**0.10**⁣^*∗∗*^	**−0.09**⁣^*∗*^	**−0.10**⁣^*∗*^	0.04	−0.07	**0.11**⁣^*∗∗*^	**0.13**⁣^*∗∗*^	**0.14**⁣^*∗∗*^	**0.12**⁣^*∗∗*^	0.06	**0.12**⁣^*∗∗*^	0.05	0.04	0.01	0.00	0.08	0.05

*Note:* Bold indicates significance at ⁣^*∗*^*p* < 0.025, ⁣^*∗∗*^*p* < 0.012.

Abbreviations: C-PTSD, complex posttraumatic stress disorder; CSAM, child sexual abuse material; PTSD, posttraumatic stress disorder.

**Table 6b:** Correlations between all measures in the study (continued).

Variable	1	2	3	4	5	6	7	8	9	10	11	12	13	14	15	16	17	18	19	20	21
28. Negative religious coping	**0.21**⁣^*∗∗*^	**0.21**⁣^*∗∗*^	**0.23**⁣^*∗∗*^	**0.24**⁣^*∗∗*^	**0.19**⁣^*∗∗*^	**−0.14**⁣^*∗∗*^	−0.07	0.05	**−0.10**⁣^*∗*^	**0.09**⁣^*∗*^	**0.18**⁣^*∗∗*^	**0.25**⁣^*∗∗*^	**0.20**⁣^*∗∗*^	0.03	**0.16**⁣^*∗∗*^	0.05	0.01	0.03	0.00	**0.13**⁣^*∗∗*^	0.08
29. Support access	−0.07	−0.08	−0.08	−0.05	**−0.11**⁣^*∗∗*^	**0.10**⁣^*∗*^	−0.03	−0.01	**0.18**⁣^*∗∗*^	−0.07	−0.01	0.01	0.03	0.03	−0.03	0.02	**0.10**⁣^*∗*^	**0.09**⁣^*∗*^	−0.01	**−0.09**⁣^*∗*^	−0.01
30. Support used	**0.07**	**0.05**	**0.09**⁣^*∗*^	**0.10**⁣^*∗*^	0.07	**−0.09**⁣^*∗*^	0.08	−0.02	0.06	**0.09**⁣^*∗*^	0.05	**0.10**⁣^*∗∗*^	**0.19**⁣^*∗∗*^	0.03	**0.17**⁣^*∗∗*^	0.04	0.06	0.07	−0.05	**0.14**⁣^*∗∗*^	−0.02
31. Support helpful	**−0.17**⁣^*∗∗*^	**−0.14**⁣^*∗∗*^	**−0.20**⁣^*∗∗*^	**−0.16**⁣^*∗∗*^	**−0.22**⁣^*∗∗*^	**0.19**⁣^*∗∗*^	**−0.11**⁣^*∗*^	0.02	**0.29**⁣^*∗∗*^	−0.06	−0.07	−0.09	0.04	−0.08	−0.09	**0.13**⁣^*∗∗*^	0.07	**0.16**⁣^*∗∗*^	0.07	−0.06	0.01
32. Barriers to support	**0.53**⁣^*∗∗*^	**0.50**⁣^*∗∗*^	**0.56**⁣^*∗∗*^	**0.49**⁣^*∗∗*^	**0.54**⁣^*∗∗*^	**−0.48**⁣^*∗∗*^	0.05	**0.14**⁣^*∗∗*^	**−0.49**⁣^*∗∗*^	**0.16**⁣^*∗∗*^	**0.25**⁣^*∗∗*^	**0.35**⁣^*∗∗*^	**0.23**⁣^*∗∗*^	**0.14**⁣^*∗∗*^	**0.33**⁣^*∗∗*^	−0.05	0.02	−0.07	**0.11**⁣^*∗∗*^	**0.31**⁣^*∗∗*^	0.06
33. Age	−0.01	−0.05	−0.01	−0.02	0.01	−0.01	**−0.10**⁣^*∗*^	0.04	0.01	−0.03	0.02	**0.10**⁣^*∗∗*^	−0.01	0.01	−0.02	−0.05	−0.01	**0.10**⁣^*∗∗*^	−0.07	0.06	0.00
34. Gender (1 = *m*, 2 = *f*)	0.00	0.07	−0.04	−0.04	−0.04	0.03	**−0.09**⁣^*∗*^	**0.23**⁣^*∗∗*^	0.04	0.03	−0.02	−0.02	−0.02	−0.04	0.04	0.**12**⁣^*∗∗*^	0.02	−0.02	0.02	0.02	0.00
35. Officer vs. staff (1 = *officer*, 2 = *staff*)	−0.06	−0.06	−0.01	−0.02	0.01	−0.04	**0.16**⁣^*∗∗*^	**−0.55**⁣^*∗∗*^	0.05	0.01	−0.07	−0.02	**−0.12**⁣^*∗∗*^	0.00	−0.07	0.02	**−0.17**⁣^*∗∗*^	**−0.09**⁣^*∗*^	**−0.12**⁣^*∗∗*^	−0.04	0.00
36. Career length	0.03	0.02	0.02	0.02	0.01	−0.01	−0.07	**0.15**⁣^*∗∗*^	−0.05	0.00	0.06	**0.12**⁣^*∗∗*^	0.08	0.02	0.03	−0.02	0.05	**0.11**⁣^*∗∗*^	0.01	0.08	0.00
37. Role length	0.00	−0.01	0.06	0.07	0.04	−004	0.04	−0.05	**−0.10**⁣^*∗*^	0.08	0.02	**0.11**⁣^*∗∗*^	−0.01	0.00	0.04	0.02	−0.04	0.04	−0.06	0.06	**0.11**⁣^*∗∗*^
38. Relationship status (1 = *partnered*, 0 = *not partnered*)	−0.05	−0.07	−0.03	−0.02	−0.04	0.04	−0.06	0.02	0.02	0.01	0.06	0.01	0.00	0.05	0.02	0.08	0.06	**0.09**⁣^*∗*^	0.08	0.04	0.03
39. Parent of <18 (1 = *yes*, 0 = *no*)	0.03	0.02	0.06	0.08	0.04	−0.02	**−0.13**⁣^*∗∗*^	0.08	−0.08	−0.03	**0.09**⁣^*∗*^	0.03	0.05	0.01	0.06	0.04	0.00	**0.09**⁣^*∗*^	0.05	0.08	0.06
40. Caregiver >18 (1 = *yes*, 0 = *no*)	0.06	0.02	0.03	0.05	0.00	−0.06	−0.05	0.06	0.00	0.01	0.00	0.04	0.00	−0.01	0.04	−0.02	0.02	0.06	0.00	0.06	0.00
41. Therapy experience (1 = *yes*, 0 = *no*)	**0.13**⁣^*∗∗*^	**0.10**⁣^*∗*^	**0.19**⁣^*∗∗*^	**0.19**⁣^*∗∗*^	**0.16**⁣^*∗*^	**−0.15**⁣^*∗∗*^	**0.09**⁣^*∗*^	−0.08	0.01	0.08	0.06	**0.13**⁣^*∗∗*^	0.08	0.00	**0.20**⁣^*∗∗*^	0.02	0.06	0.06	−0.06	**0.13**⁣^*∗∗*^	−0.01
42. Religiosity	**0.13**⁣^*∗∗*^	**0.09**⁣^*∗*^	**0.10**⁣^*∗*^	**0.11**⁣^*∗∗*^	0.07	**−0.09**⁣^*∗*^	**−0.15**⁣^*∗∗*^	0.08	−0.04	0.03	0.07	0.07	0.07	0.01	**0.12**⁣^*∗∗*^	0.01	0.05	−0.02	−0.03	0.07	−0.02

*Note:* Bold indicates significance at ⁣^*∗*^*p* < 0.025, ⁣^*∗∗*^*p* < 0.012.

**Table 6c:** Correlations between all measures in the study (continued).

Variable	22	23	24	25	26	27	28	29	30	31	32	33	34	35	36	37	38	39	40	41
22. Seeking distraction	—	—	—	—	—	—	—	—	—	—	—	—	—	—	—	—	—	—	—	—
23. Withdrawal	**0.19**⁣^*∗∗*^	—	—	—	—	—	—	—	—	—	—	—	—	—	—	—	—	—	—	—
24. Approach	**0.12**⁣^*∗∗*^	**−0.21**⁣^*∗∗*^	—	—	—	—	—	—	—	—	—	—	—	—	—	—	—	—	—	—
25. Social support	**0.19**⁣^*∗∗*^	**−0.17**⁣^*∗∗*^	**0.34**⁣^*∗∗*^	—	—	—	—	—	—	—	—	—	—	—	—	—	—	—	—	—
26. Ignoring	**0.29**⁣^*∗∗*^	**0.54**⁣^*∗∗*^	**−0.24**⁣^*∗∗*^	**−0.27**⁣^*∗∗*^	—	—	—	—	—	—	—	—	—	—	—	—	—	—	—	—
27. Positive religious coping	0.02	**0.12**⁣^*∗∗*^	0.07	0.01	0.08	—	—	—	—	—	—	—	—	—	—	—	—	—	—	—
28. Negative religious coping	0.04	**0.16**⁣^*∗∗*^	−0.02	0.04	**0.11**⁣^*∗∗*^	**0.53**⁣^*∗∗*^	—	—	—	—	—	—	—	—	—	—	—	—	—	—
29. Support access	−0.03	−0.03	**0.14**⁣^*∗∗*^	**0.09**⁣^*∗*^	**−0.12**⁣^*∗∗*^	0.04	0.02	—	—	—	—	—	—	—	—	—	—	—	—	—
30. Support used	0.03	**0.08**⁣^*∗*^	0.02	**0.16**⁣^*∗∗*^	−0.04	**0.09**⁣^*∗*^	0.07	**0.13**⁣^*∗∗*^	—	—	—	—	—	—	—	—	—	—	—	—
31. Support helpful	0.09	**−0.14**⁣^*∗∗*^	**0.17**⁣^*∗∗*^	**0.23**⁣^*∗∗*^	**−0.20**⁣^*∗∗*^	0.01	0.04	**0.23**⁣^*∗∗*^	**0.25**⁣^*∗∗*^	—	—	—	—	—	—	—	—	—	—	—
32. Barriers to support	0.08	**0.45**⁣^*∗∗*^	**−0.23**⁣^*∗∗*^	**−0.19**⁣^*∗∗*^	**0.44**⁣^*∗∗*^	**0.13**⁣^*∗∗*^	**0.18**⁣^*∗∗*^	**−0.13**⁣^*∗∗*^	−0.05	**−0.26**⁣^*∗∗*^	—	—	—	—	—	—	—	—	—	—
33. Age	−0.08	0.01	−0.02	−0.04	**−0.08**⁣^*∗*^	0.07	0.05	**0.11**⁣^*∗∗*^	**0.10**⁣^*∗*^	**0.13**⁣^*∗∗*^	**−0.11**⁣^*∗∗*^	—	—	—	—	—	—	—	—	—
34. Gender (1 = *m*, 2 = *f*)	0.04	−0.04	0.06	**0.24**⁣^*∗∗*^	−0.05	−0.02	−0.03	−0.06	0.03	0.09	0.00	**−0.10**⁣^*∗*^	—	—	—	—	—	—	—	—
35. Officer vs. staff (1 = *officer*, 2 = *staff*)	0.06	−0.02	0.06	**−0.10**⁣^*∗*^	0.01	−0.04	−0.03	−0.06	0.01	−0.01	**−0.11**⁣^*∗∗*^	**−0.16**⁣^*∗∗*^	**−0.09**⁣^*∗*^	—	—	—	—	—	—	—
36. Career length	−0.04	0.06	−0.03	0.00	−0.03	0.03	0.06	**0.11**⁣^*∗∗*^	**0.11**⁣^*∗∗*^	**0.09**⁣^*∗*^	−0.01	**0.72**⁣^*∗∗*^	−0.06	**−0.38**⁣^*∗∗*^	—	—	—	—	—	—
37. Role length	−0.01	0.05	0.00	−0.03	0.01	0.02	0.03	−0.04	**0.14**⁣^*∗∗*^	−0.03	−0.03	**0.38**⁣^*∗∗*^	−0.04	**0.11**⁣^*∗∗*^	**0.38**⁣^*∗∗*^	—	—	—	—	—
38. Relationship status (1 = *partnered*, 0 = *not partnered*)	0.01	−0.02	0.06	−0.01	0.03	0.04	−0.02	0.04	0.00	0.02	−0.02	**0.34**⁣^*∗∗*^	**−0.13**⁣^*∗∗*^	**−0.16**⁣^*∗∗*^	**0.31**⁣^*∗∗*^	**0.16**⁣^*∗∗*^	—	—	—	—
39. Parent of <18 (1 = *yes*, 0 = *no*)	−0.01	−0.02	0.05	0.02	0.01	0.05	0.05	0.00	0.01	0.03	0.01	**0.25**⁣^*∗∗*^	−0.06	**−0.19**⁣^*∗∗*^	**0.39**⁣^*∗∗*^	**0.19**⁣^*∗∗*^	**0.41**⁣^*∗∗*^	—	—	—
40. Caregiver >18 (1 = *yes*, 0 = *no*)	−0.04	0.02	0.06	0.02	−0.07	0.05	0.07	0.07	0.07	**0.12**⁣^*∗*^	−0.02	**0.18**⁣^*∗∗*^	0.07	0.00	**0.17**⁣^*∗∗*^	0.07	0.05	0.06	—	—
41. Therapy experience (1 = *yes*, 0 = *no*)	0.05	**0.14**⁣^*∗∗*^	−0.02	**0.10**⁣^*∗∗*^	0.03	0.03	**0.09**⁣^*∗*^	**0.08**⁣^*∗*^	**0.3**⁣^*∗∗*^	**0.16**⁣^*∗∗*^	0.04	**0.10**⁣^*∗*^	−0.05	0.04	**0.11**⁣^*∗∗*^	**0.11**⁣^*∗∗*^	0.04	−0.02	0.03	—
42. Religiosity	−0.01	**0.11**⁣^*∗∗*^	0.05	0.05	0.04	**0.62**⁣^*∗∗*^	**0.30**⁣^*∗∗*^	−0.02	**0.08**⁣^*∗*^	0.03	**0.08**⁣^*∗*^	**0.09**⁣^*∗*^	0.03	**−0.10**⁣^*∗∗*^	**0.09**⁣^*∗*^	0.02	0.05	0.07	0.07	0.02

*Note:* Bold indicates significance at ⁣^*∗*^*p* < 0.025, ⁣^*∗∗*^*p* < 0.012.

**Table 7:** Exposure to CSAM, contact with victims and perpetrators, and job success and support predict moral injury, depression, generalized anxiety, C-PTSD, and wellbeing beyond demographics.

Predictors	Moral injury			Depression			Generalized anxiety			C-PTSD			Wellbeing		
	*β*	*t*	*p*	*β*	*t*	*p*	*β*	*t*	*p*	*β*	*t*	*p*	*β*	*t*	*p*
Step 1															
Age	−0.05	−0.78	0.436	−0.06	−1.03	0.302	−0.10	−1.71	0.087	−0.05	−0.82	0.410	0.01	0.23	0.821
Gender (1 = *m*, 2 = *f*)	0.00	−0.04	0.970	−0.01	−0.37	0.710	0.06	1.60	0.110	−0.04	−1.01	0.313	0.03	0.76	0.449
Officer vs. staff (1 = *officer*, 2 = *staff*)	−0.05	−1.06	0.292	−0.06	−1.30	0.195	−0.04	−0.83	0.406	−0.02	−0.46	0.646	−0.03	−0.73	0.467
Career length	0.03	0.48	0.631	0.02	0.38	0.705	0.06	0.88	0.379	−0.02	−0.37	0.710	0.00	0.05	0.956
Role length	0.07	1.58	0.115	0.01	0.13	0.899	0.00	−0.01	0.994	0.06	1.38	0.168	−0.02	−0.48	0.633
Relationship status (1 = *partnered*, 0 = *not partnered*)	0.02	0.47	0.635	−0.08	−1.82	0.069	−0.08	−1.78	0.076	−0.09	−2.02	0.044	0.08	1.71	0.087
Parent of <18 (1 = *yes*, 0 = *no*)	0.01	0.26	0.798	0.05	1.03	0.302	0.05	1.01	0.312	0.10	2.18	0.030	−0.06	−1.21	0.227
Caregiver >18 (1 = *yes*, 0 = *no*)	0.00	0.10	0.919	0.05	1.19	0.233	0.02	0.40	0.688	0.02	0.60	0.548	−0.05	−1.31	0.189
Therapy experience (1 = *yes*, 0 = *no*)	**0.10**	**2.64**	**0.008**	**0.14**	**3.46**	**<.001**	**0.11**	**2.84**	**0.005**	**0.19**	**4.83**	**<0.001**	**−0.14**	**−3.53**	**<0.001**
Religiosity	0.07	1.78	0.075	**0.13**	**3.21**	**<0.001**	0.09	2.24	0.025	**0.09**	**2.26**	**0.024**	−0.07	−1.88	0.060
	*R* ^2^ = 0.028			*R* ^2^ = 0.047			*R* ^2^ = 0.039			*R* ^2^ = 0.059			*R* ^2^ = 0.036		
Step 2															
Exposure to child sexual abuse material	**0.15**	**3.95**	**<0.001**	0.04	1.08	0.281	0.02	0.43	0.670	0.08	2.24	0.025	−0.06	−1.85	0.065
Contact with victims and perpetrators	0.08	1.73	0.083	**0.17**	**3.95**	**<0.001**	**0.13**	**3.00**	**0.003**	**0.17**	**4.26**	**<0.001**	−0.05	−1.17	0.244
Job success and support	**−0.34**	**−9.11**	**<0.001**	**−0.44**	**−12.59**	**<0.001**	**−0.38**	**−10.51**	**<0.001**	**−0.50**	**−14.81**	**<0.001**	**0.54**	**16.11**	**<0.001**
	*R* ^2^ = 0.163			*R* ^2^ = 0.269			*R* ^2^ = 0.204			*R* ^2^ = 0.337			*R* ^2^ = 0.329		

*Note:* Bold indicates significance at *p* < 0.025.

Abbreviations: C-PTSD, complex posttraumatic stress disorder; CSAM, child sexual abuse material.

**Table 8:** Moral injury subscales: transgression-self, transgression-other, and betrayal, predict depression, generalized anxiety, C-PTSD, and wellbeing beyond demographics.

Predictors	Depression			Generalized anxiety			C-PTSD			Wellbeing		
	*β*	*t*	*p*	*β*	*t*	*p*	*β*	*t*	*p*	*β*	*t*	*p*
Step 1												
Age	−0.06	−1.06	0.290	−0.11	−1.78	0.075	−0.05	−0.88	0.378	0.03	0.44	0.659
Gender (1 = *m*, 2 = *f*)	−0.02	−0.40	0.692	0.06	1.56	0.119	−0.04	−1.07	0.283	0.03	0.86	0.390
Officer vs. Staff (1 = *officer*, 2 = *staff*)	−0.06	−1.33	0.185	−0.04	−0.87	0.385	−0.02	−0.53	0.597	−0.03	−0.65	0.513
Career length	0.03	0.41	0.685	0.06	0.95	0.342	−0.02	−0.31	0.754	−0.01	−0.14	0.885
Role length	0.00	0.11	0.910	0.00	−0.02	0.982	0.06	1.34	0.179	−0.02	−0.45	0.652
Relationship status (1 = *partnered*, 0 = *not partnered*)	−0.08	−1.79	0.074	−0.08	−1.71	0.088	−0.09	−1.95	0.051	0.07	1.54	0.123
Parent of <18 (1 = *yes*, 0 = *no*)	0.04	0.99	0.323	0.04	0.93	0.354	0.09	2.07	0.039	−0.05	−1.00	0.317
Caregiver >18 (1 = *yes*, 0 = *no*)	0.05	1.20	0.229	0.02	0.42	0.676	0.02	0.63	0.532	−0.05	−1.36	0.175
Therapy experience (1 = *yes*, 0 = *no*)	**0.14**	**3.48**	**0.001**	**0.11**	**2.87**	**0.004**	**0.19**	**4.88**	**<0.001**	**−0.14**	**−3.61**	**<0.001**
Religiosity	**0.13**	**3.22**	**0.001**	**0.09**	**2.27**	**0.023**	**0.09**	**2.30**	**0.022**	−0.08	−1.96	0.050
	*R* ^2^ = 0.048			*R* ^2^ = 0.040			*R* ^2^ = 0.060			*R* ^2^ = 0.037		
Step 2												
Transgression-self	0.01	0.30	0.765	−0.01	−0.14	0.888	**0.10**	**2.32**	**0.021**	**−0.10**	**−2.37**	**0.018**
Transgression-other	0.02	0.46	0.646	0.04	0.98	0.325	0.06	1.70	0.090	−0.03	−0.82	0.413
Betrayal	**0.35**	**8.13**	**<0.001**	**0.32**	**7.51**	**<0.001**	**0.33**	**7.98**	**<0.001**	**−0.28**	**−6.54**	**<0.001**
	*R* ^2^ = 0.172			*R* ^2^ = 0.148			*R* ^2^ = 0.225			*R* ^2^ = 0.160		

*Note:* Bold indicates significance at *p* < 0.025.

Abbreviation: C-PTSD, complex posttraumatic stress disorder.

**Table 9:** Cognitive and emotional regulation subscales: self-blame, acceptance, rumination, positive refocusing, planning, positive reappraisal, putting into perspective, catastrophizing, and other-blame predict moral injury, depression, generalized anxiety, C-PTSD, and wellbeing beyond demographics.

Predictors	Moral injury			Depression			Generalized anxiety			C-PTSD			Wellbeing		
	*β*	*t*	*p*	*β*	*t*	*p*	*β*	*t*	*p*	*β*	*t*	*p*	*β*	*t*	*p*
Step 1															
Age	−0.04	−0.65	0.515	−0.05	−0.82	0.414	−0.09	−1.51	0.130	−0.04	−0.62	0.537	−0.01	−0.13	0.898
Gender (1 = *m*, 2 = *f*)	0.00	−0.11	0.909	−0.02	−0.44	0.663	0.06	1.50	0.135	−0.04	−1.13	0.261	0.03	0.86	0.389
Officer vs. staff (1 = *officer*, 2 = *staff*)	−0.04	−0.91	0.365	−0.06	−1.27	0.206	−0.04	−0.82	0.414	−0.02	−0.47	0.641	−0.04	−0.78	0.437
Career length	0.03	0.48	0.633	0.02	0.27	0.785	0.05	0.78	0.433	−0.03	−0.48	0.633	0.02	0.25	0.800
Role length	0.07	1.63	0.104	0.01	0.15	0.882	0.00	0.04	0.965	0.06	1.37	0.170	−0.02	−0.44	0.663
Relationship status (1 = *partnered*, 0 = *not partnered*)	0.02	0.40	0.692	−0.08	−1.90	0.058	−0.08	−1.90	0.059	−0.09	−2.08	0.038	0.08	1.83	0.068
Parent of <18 (1 = *yes*, 0 = *no*)	0.01	0.28	0.782	0.05	1.03	0.305	0.04	0.96	0.339	0.10	2.17	0.031	−0.06	−1.26	0.209
Caregiver >18 (1 = *yes*, 0 = *no*)	0.01	0.25	0.801	0.05	1.36	0.175	0.02	0.56	0.574	0.03	0.74	0.461	−0.06	−1.60	0.110
Therapy experience (1 = *yes*, 0 = *no*)	**0.10**	**2.50**	**0.013**	**0.13**	**3.43**	**0.001**	**0.11**	**2.82**	**0.005**	**0.19**	**4.83**	**<0.001**	**−0.14**	**−3.46**	**0.001**
Religiosity	0.07	1.65	0.098	**0.12**	**3.13**	**0.002**	0.09	2.18	0.029	0.09	2.16	0.031	−0.07	−1.83	0.068
	*R* ^2^ = 0.026			*R* ^2^ = 0.047			*R* ^2^ = 0.038			*R* ^2^ = 0.059			*R* ^2^ = 0.038		
Step 2															
Self-blame	**0.16**	**4.09**	**<0.001**	**0.13**	**3.40**	**<0.001**	0.08	2.12	0.034	**0.13**	**3.54**	**<0.001**	**−0.17**	**−4.33**	**<0.001**
Acceptance	0.02	0.51	0.614	0.01	0.30	0.764	0.02	0.43	0.667	0.02	0.61	0.543	0.04	1.02	0.308
Rumination	**0.26**	**4.95**	**<0.001**	**0.20**	**4.24**	**<0.001**	**0.22**	**4.41**	**<0.001**	**0.27**	**5.95**	**<0.001**	**−0.17**	**−3.42**	**<0.001**
Positive refocusing	−0.06	−1.59	0.113	**−0.17**	**−4.72**	**<0.001**	**−0.13**	**−3.61**	**<0.001**	**−0.18**	**−5.26**	**<0.001**	**0.19**	**5.12**	**<0.001**
Planning	−0.07	−1.85	0.065	−0.03	−0.82	0.414	−0.02	−0.54	0.587	−0.07	−2.01	0.045	**0.11**	**2.93**	**0.004**
Positive reappraisal	−0.04	−0.92	0.357	**−0.10**	**−2.46**	**0.014**	−0.05	−1.33	0.183	−0.03	−0.86	0.389	0.07	1.63	0.104
Putting into perspective	0.03	0.80	0.425	**0.09**	**2.39**	**0.017**	0.07	1.73	0.084	0.06	1.50	0.133	**−0.09**	**−2.26**	**0.024**
Catastrophizing	**0.13**	**2.51**	**0.012**	**0.28**	**5.96**	**<0.001**	**0.31**	**6.40**	**<0.001**	**0.29**	**6.34**	**<0.001**	**-0.25**	**−5.13**	**<0.001**
Other-blame	**0.16**	**4.12**	**<0.001**	−0.01	−0.22	0.823	0.01	0.23	0.815	0.04	1.28	0.200	−0.04	−1.10	0.273
	*R* ^2^ = 0.235			*R* ^2^ = 0.331			*R* ^2^ = 0.311			*R* ^2^ = 0.399			*R* ^2^ = 0.293		

*Note:* Bold indicates significance at *p* < 0.025.

Abbreviation: C-PTSD, complex posttraumatic stress disorder.

**Table 10:** Behavioral coping subscales seeking distraction, withdrawal, approach, planning, social support, and ignoring predict moral injury, depression, generalized anxiety, C-PTSD, and wellbeing beyond demographics.

Predictors	Moral injury			Depression			Generalized anxiety			C-PTSD			Wellbeing		
	*β*	*t*	*p*	*β*	*t*	*p*	*β*	*t*	*p*	*β*	*t*	*p*	*β*	*t*	*p*
Step 1															
Age	−0.04	−0.68	0.496	−0.05	−0.84	0.400	−0.09	−1.53	0.125	−0.04	−0.65	o.515	−0.01	−0.09	0.931
Gender (1 = *m*, 2 = *f*)	0.00	−0.02	0.985	−0.02	−0.41	0.684	0.06	1.53	0.127	−0.04	−1.07	o.285	0.03	0.74	0.460
Officer vs. staff (1 = *officer*, 2 = *staff*)	−0.04	−0.91	0.364	−0.06	−1.29	0.197	−0.04	−0.84	0.404	−0.02	−0.49	0.625	−0.04	−0.77	0.440
Career length	0.03	0.50	0.616	0.02	0.29	0.773	0.05	0.80	0.426	−0.03	−0.45	0.649	0.01	0.22	0.826
Role length	0.07	1.67	0.096	0.00	0.10	0.917	0.00	0.02	0.983	0.06	1.35	0.177	−0.02	−0.49	0.627
Relationship status (1 = *partnered*, 0 = *not partnered*)	0.01	0.32	0.746	−0.09	−1.95	0.052	−0.09	−1.94	0.053	−0.10	−2.15	0.032	0.09	1.91	0.056
Parent of <18 (1 = *yes*, 0 = *no*)	0.01	0.19	0.847	0.04	0.99	0.323	0.04	0.93	0.355	0.09	2.10	0.036	−0.05	−1.15	0.252
Caregiver >18 (1 = *yes*, 0 = *no*)	0.01	0.25	0.802	0.05	1.35	0.179	0.02	0.56	0.579	0.03	0.73	0.468	−0.06	−1.59	0.112
Therapy experience (1 = *yes*, 0 = *no*)	**0.10**	**2.50**	**0.013**	**0.13**	**3.40**	**0.001**	**0.11**	**2.80**	**0.005**	**0.19**	**4.80**	**<0.001**	**−0.14**	**−3.46**	**0.001**
Religiosity	0.07	1.74	0.082	**0.13**	**3.22**	**0.001**	0.09	2.25	0.025	**0.09**	**2.27**	**0.023**	−0.08	−1.94	0.053
	*R* ^2^ = 0.026			*R* ^2^ = 0.048			*R* ^2^ = 0.039			*R* ^2^ = 0.059			*R* ^2^ = 0.038		
Step 2															
Seeking distraction	0.00	0.11	0.912	**−0.11**	**−3.40**	**0.001**	−0.08	−2.07	0.038	**−0.08**	**−2.38**	**0.018**	**0.15**	**4.29**	**<0.001**
Withdrawal	**0.26**	**5.66**	**<0.001**	**0.50**	**13.59**	**<0.001**	**0.44**	**10.94**	**<0.001**	**0.53**	**14.81**	**<0.001**	**−0.47**	**−12.28**	**<0.001**
Approach	−0.07	−1.63	0.104	0.02	0.48	0.629	0.00	−0.05	0.957	−0.04	−1.09	0.275	**0.10**	**2.95**	**0.003**
Social support	0.05	1.24	0.217	**−0.09**	**−2.51**	**0.012**	−0.03	−0.79	0.429	**−0.10**	**−2.96**	**0.003**	**0.13**	**3.50**	**0.001**
Ignoring	**0.13**	**2.74**	**0.006**	**0.18**	**4.59**	**<0.001**	**0.15**	**3.62**	**<0.001**	**0.16**	**4.20**	**<0.001**	**−0.13**	**−3.22**	**0.001**
	*R* ^2^ = 0.149			*R* ^2^ = 0.427			*R* ^2^ = 0.318			*R* ^2^ = 0.479			*R* ^2^ = 0.411		

*Note:* Bold indicates significance at *p* < 0.025.

Abbreviation: C-PTSD, complex posttraumatic stress disorder.

**Table 11:** Positive and negative religious coping predicts moral injury, depression, generalized anxiety, C-PTSD, and wellbeing beyond demographics.

Predictors	Moral injury			Depression			Generalized anxiety			C-PTSD			Wellbeing		
	*β*	*t*	*p*	*β*	*t*	*p*	*β*	*t*	*p*	*β*	*t*	*p*	*β*	*t*	*p*
Step 1															
Age	−0.08	−1.26	0.210	−0.05	−0.90	0.367	−0.09	−1.55	0.122	−0.04	−0.66	0.511	−0.01	−0.15	0.884
Gender (1 = *m*, 2 = *f*)	0.00	0.07	0.942	−0.02	−0.46	0.645	0.06	1.56	0.120	−0.04	−1.08	0.280	0.03	0.86	0.388
Officer vs staff (1 = *officer*, 2 = *staff*)	−0.04	−0.92	0.356	−0.06	−1.34	0.181	−0.04	−0.83	0.404	−0.02	−0.49	0.624	−0.03	−0.71	0.480
Career length	0.05	0.82	0.413	0.02	0.32	0.747	0.05	0.78	0.436	−0.03	−0.47	0.637	0.02	0.36	0.718
Role length	0.07	1.54	0.123	0.00	0.11	0.913	0.00	−0.01	0.993	0.06	1.34	0.182	−0.02	−0.40	0.692
Relationship status (1 = *partnered*, 0 = *not partnered*)	0.03	0.62	0.538	−0.08	−1.85	0.065	−0.08	−1.85	0.065	−0.09	−2.13	0.033	0.08	1.82	0.069
Parent of <18 (1 = *yes*, 0 = *no*)	0.02	0.39	0.694	0.04	0.98	0.328	0.05	1.03	0.305	0.10	2.20	0.028	−0.06	−1.34	0.179
Caregiver >18 (1 = *yes*, 0 = *no*)	0.00	0.10	0.917	0.05	1.17	0.241	0.02	0.39	0.693	0.02	0.60	0.550	−0.05	−1.30	0.195
Therapy experience (1 = *yes*, 0 = *no*)	**0.10**	**2.64**	**0.009**	**0.13**	**3.40**	**0.001**	**0.11**	**2.82**	**0.005**	**0.19**	**4.81**	**<0.001**	−0.14	−3.50	**<0.001**
Religiosity	0.07	1.81	0.071	**0.13**	**3.20**	**0.001**	0.09	2.22	0.027	**0.09**	**2.25**	**0.024**	−0.07	−1.81	0.070
	*R* ^2^ = 0.029			*R* ^2^ = 0.046			*R* ^2^ = 0.038			*R* ^2^ = 0.059			*R* ^2^ = 0.036		
Step 2															
Positive religious coping	0.09	1.65	0.099	0.01	0.24	0.809	−0.02	−0.34	0.731	0.02	0.30	0.761	0.01	0.14	0.892
Negative religious coping	**0.18**	**3.90**	**<0.001**	**0.16**	**3.48**	**<0.001**	0.20	4.25	**<0.001**	**0.19**	**4.28**	**<0.001**	**−0.11**	**−2.27**	**0.024**
	*R* ^2^ = 0.075			*R* ^2^ = 0.071			*R* ^2^ = 0.070			*R* ^2^ = 0.096			*R* ^2^ = 0.046		

*Note:* Bold indicates significance at *p* < 0.025.

Abbreviation: C-PTSD, complex posttraumatic stress disorder.

## Data Availability

All data, analysis code, and research materials are available at: https://osf.io/5ru7h/?view_only=5f5f066819ec436ab547533977feaf00.

## Endnotes

^1^We also examined reports of access, usage, utility, and barriers to various support services currently available to police, which we report elsewhere (see [23] and Supporting Information 4: File S4).

^2^We converted scores from the typical presentation of results as a sum from 0 to 60 to a mean from 1 to 7.

^3^That said, we asked about symptoms over the past month, whereas Smith et al. [71] asked about symptoms in the past 2 weeks, which makes comparisons challenging.

^4^However, younger people reported worse mental health in the online comparison sample.

## Supporting Information

Supporting Information 1 File S1: Original Police Sample provides the methods, results, and brief discussion of the 575 police officers and staff who completed the original sample in line with preregistration before we expanded the sample to include Scotland and Northern Ireland, including tables presenting correlations and regressions.

Supporting Information 2 File S2: Online Sample provides the methods, results, and brief discussion of the UK online civilian comparison sample, including tables presenting correlations and regressions.

Supporting Information 3 File S3: Study Materials provides the exact wording of all measures, instructions, and materials in the study, as well as a copy of the recruitment email sent to participants.

Supporting Information 4 File S4: Resources and Barriers provides the methods and results of additional measures included in the study but not in the main text, assessing perceptions of resource availability, use, helpfulness, and barriers.

Supporting Information 5 Appendix A. Data Transparency clarifies which analyses are unique to this paper and which overlap with Redmond et al. [23], who examined the same dataset.
