# Supplementary material for: Protecting the Protectors: Moral Injury, Coping Styles, and Mental Health of UK Police Officers and Staff Investigating Child Sexual Abuse Material
Source: Depress Anxiety. 2024 Nov 23;2024:1854312. doi: 10.1155/da/1854312 (PMC11922302; doi:10.1155/da/1854312)
Supplement: Supporting Information 1 — File S1: Original Police Sample provides the methods, results, and brief discussion of the 575 police officers and staff who completed the original sample in line with preregistration before we expanded the sample to include Scotland and Northern Ireland, including tables presenting correlations and regressions. [file 1854312.f1.docx]

**Online Supplemental Material: Original Police Sample (No Scotland, No Ireland)**

**Protecting the Protectors: Moral Injury, Coping Styles, and Mental Health of UK Police Officers and Staff Investigating Child Sexual Assault and Exploitation**

**Method**

**Participants**

We originally recruited 896 participants. We removed nine participants who refused the reconsent, 120 who completed less than 50% of the study, and 37 people who completed the study in less than 200 seconds with few responses, leaving a sample of 730. Finally, we selected all 575 participants who passed an attention check buried in the GAD measure: *Over the last month, how often have you been bothered by any of the following problems? If you are paying attention, please select 'nearly every day' for this question* (Oppenheimer et al., 2011).

Hence, we obtained a final sample of 575 participants: 396 police officers and 179 police staff, *M*_age_ = 40.70, *SD* = 9.34, 235 male, 337 female, 1 nonbinary, and two did not report gender. Regarding marital status, 320 participants reported they were married (55.7%), 124 reported they were single (21.6%), 84 living as married (14.6%), 31 divorced (5.4%), 14 separated (2.4%), and two (0.3%) did not report marital status. The vast majority (*n* = 556) identified as *White or Caucasian* (96.7%), with a few participants identifying as *Black or Caribbean* (*n* = 8, 1.4%), *Asian or Indian* (*n* = 4, 0.7%), or Arabic (*n* = 1, 0.2%), five participants (0.9%) did not report ancestry.

Regarding parental status, 319 participants reported they were a parent or guardian for children under 18 (55.5%), 254 reported they were not a parent or guardian for someone under 18 (44.2%), and 2 (0.3%) cases were coded as missing values.^[[1]](#footnote-1)^ We also asked if participants were caregivers for someone over 18 (e.g., elderly parent): 523 reported they were not a caregiver (91.0%), 50 reported they were a caregiver (8.7%), and 2 (0.3%) cases were coded as missing values.^[[2]](#footnote-2)^ Regarding therapy, 373 participants (64.9%) reported never receiving therapy for any reason, whereas 196 (34.1%) reported either receiving therapy at some point either currently or in the past, and 6 (1%) did not respond to this question.

Most participants reported working more than a year as a police officer or staff, with an average service length of 10-14 years (*M* = 7.22, *SD* = 1.56) on a scale from 1 (*only a few days*) to 9 (*over 20 years*). Most participants reported working at least a year in their current role, with an average service length of over two years (*M* = 4.77, *SD* = 1.25, on the same 9-point scale). Participants served all regions of England and Wales (see Table 1). All participants reported that they were currently active members of UK police forces or police staff of any rank in England and Wales who routinely and regularly deal with child sexual abuse and exploitation (CSAE), except for thirteen people who clarified factors such as ‘recent retirement’ or being recently removed from CSAE investigations due to PTSD. We nonetheless retained these participants due to their recent service.

Most participants reported roles in the *Child Protection Unit*, *Investigations*, or *Police Online Investigation Team*, though a minority reported a wide variety of other roles, such as *Intelligence*, *Management of Sexual or Violent Offenders*, *Operational and Administrative Support*, *Neighborhood Policin*g, and *Criminal Justice* (see Table 2). Participants often described themselves as *detective constable*, *detective inspector*, *detective sergeant*, or some as specialized roles such as *advanced investigations officer*, or heads of specific departments. Others described themselves as *case workers*, *case review officers*, *offender managers*, *computer forensic specialists*, *typists*, or a variety of other positions.

On average, few participants reported strong spiritual or religious beliefs (*M* = 2.01, on a 7-point scale, *SD* = 1.36). Regarding their faith, 276 identified as atheist or agnostic (48%), 236 identified as Christian, Anglican, or Church of England (41%), 27 identified as spiritual (4.7%) 17 as Catholic (3%), 3 as Muslim (0.5%) 2 as Pagan (0.3%), 1 as Sikh (0.2%), 1 as Jewish (0.2%), 1 as Buddhist (0.2%), 1 as Shinto (0.2%), and 10 (1.7%) did not report a religious affiliation.

**Table 1**

*UK Region Participants Reported Worked In*

| Region | *N* | Percent |
| --- | --- | --- |
| Southeast | 92 | 16.0 |
| Southwest | 53 | 9.2 |
| London | 47 | 8.2 |
| East Midlands | 49 | 8.5 |
| West Midlands | 53 | 9.2 |
| Eastern | 51 | 8.9 |
| Northeast | 72 | 12.5 |
| Northwest | 73 | 12.7 |
| Wales | 15 | 2.6 |
| Scotland | X | X |
| Northern Ireland | X | X |
| Other (e.g., Yorkshire, National) | 68 | 11.8 |
| Unreported | 2 | .3 |
| Total | 575 | 100.0 |

**Table 2**

*Roles Participants Reported*

| Role | *N* | Percent |
| --- | --- | --- |
| Child Protection Unit | 205 | 35.7 |
| Investigations | 141 | 24.5 |
| Other (e.g., call handler, covert, forensics, school liaison officer) | 134 | 23.3 |
| POLIT (Police Online Investigation Team) | 116 | 20.2 |
| Intelligence | 32 | 5.6 |
| MOSOVO (Management of Sexual or Violent Offenders) | 22 | 3.8 |
| Operational Support | 21 | 3.7 |
| Administrative Support | 13 | 2.3 |
| Criminal Justice | 13 | 2.3 |
| Neighborhood Policing | 5 | 1.0 |
| National Policing | 5 | 0.9 |
| Response Policing | 4 | 0.7 |
| Training | 3 | 0.5 |
| Total | 714 | 124.5 |

*Note:* responses sum to more than 100% and total more than participant number because participants could select *all that apply*

**Procedure and Materials**

Participants completed all measures online via Qualtrics.

***Demographics***

Participants reported their age, gender, ethnicity, whether they are *police officer* or *police staff*, rank (if police) or job title (if civilian), the UK region they work in, the role they have, their length of experience in both their current role and their police career from 1 (*only a few days*) to 9 (*over 20 years*). They also reported marital status, whether they are a parent or other caregiver to both children under 18 and to people over 18 (*yes/no/its complicated*) with a chance to clarify. Finally, participants reported how spiritual/religious they are on a scale from 1 (*not at all spiritual/religious*) to 7 (*extremely spiritual/religious*), as well as the faith (or lack of faith) they most identify with, and whether they have ever been in therapy (open ended).

***Contact, Exposure, and Support Factors***

We asked participants how often during their police duties they encountered the following situations on scales from 1 (*almost never*) to 7 (*almost always*). As preregistered, we conducted a principal components analysis with oblimin rotation allowing for 500 iterations before convergence and 500 for rotation, retaining all factors with an eigenvalue greater than 1 (Kaiser, 1961) that were readily interpretable (Lee & Ashton, 1995). For each factor, we will retained all items loading .5 or greater on their respective factor, and no greater than .3 on any other factor. As expected, this analysis revealed three factors with an (3.66, 2.99, 2.21), together explaining a total of 63.25% of the variance. Although we originally intended this measure to assess risk factors (handling CASE material and contact with victims and perpetrators), job success factors (Assisting with arrests or convictions for child sexual abuse and exploitation), and support factors (Feeling supported by my supervisor or team), the PCA analysis suggested a somewhat different three-factor solution.

Four items appeared to track contact with both victims and perpetrators (α = .91): *Contact (in person, phone, internet) with families of victims*, *Contact (in person, phone, internet) with victims*, *Supporting victims and/or their families understand and recover from child sexual abuse and exploitation (CSAE)*, and *Contact (in person, phone, internet) with perpetrators or suspects*. Two items tracked exposure to viewing CASE material (α = .98): Vie*wing, grading, or handling photos of child sexual abuse and exploitation (CSAE)*, and *Viewing, grading, or handling videos of child sexual abuse and exploitation (CSAE)*. Finally, six items tracked success and support (α = .76): *Feeling supported by my supervisor or team*, *Feeling like my voice is heard and I have input into decisions that affect me, Feeling my work is valuable and meaningful, Feeling encouraged to seek mental health support from my supervisor or team*, *Taking time to rest and pursue activities outside of work*, and *Feeling supported by my partner, friends, or family* (see Charman & Bennett, 2021). Hence, we computed the mean for each subscale. Two items loaded on multiple factors and hence were discarded from analysis: *succeeding in protecting a child from child sexual abuse and exploitation (CSAE)*, *Assisting with arrests or convictions for child sexual abuse and exploitation (CSAE)*. We will also ask the open-ended question Is *there anything else that you routinely do in your role that you want us to know about?*

***The Moral Injury Events Scale (MIES)***

Participants completed a version of the moral injury events scale (Nash et al., 2013) slightly adapted for police rather than military use (e.g., referring to colleagues rather than fellow service members). They considered the following prompt: *Often when dealing with sexually explicit material involving children people see or do things that leave them questioning morality. For example, CSAE investigators are sometimes required to empathise or build bonds with suspects who are being investigated, which, for some, is morally difficult to do and cope with. In other cases the right course of action can be complex or unclear. People’s experiences of morality can influence their wellbeing, so we are asking about your feelings about morality to better understand how we can help people who are struggling with these issues. When thinking about your involvement with investigations into child sexual abuse and exploitation (CSAE), how much do you agree with the following statements?* The participants rated agreement with nine statements on scales from 1 (*strongly disagree*) to 7 (*strongly agree*).

Again, we conducted a principal components analysis with oblimin rotation allowing for 500 iterations before convergence and 500 for rotation, retaining all factors with an eigenvalue greater than 1 (Kaiser, 1961) that are readily interpretable (Lee & Ashton, 1995). For each factor, we retained all items loading .5 or greater on their respective factor, and no greater than .3 on any other factor. As preregistered, and consistent with Bryan and colleagues (2016), we found evidence for three factors with eigenvalues greater than 1 (4.20, 1.36, 2.00), together explaining a total of 75.14% of the variance. Moreover, items loaded as expected: two items loading on *Transgressions-Other* factor (e.g., *I saw things that were morally wrong,* α = .67), four items loading on *Transgressions-Self* factor (e.g., *I acted in ways that violated my own moral code or values,* α = .90), and three items loading on *Betrayal* factor (e.g., *I feel betrayed by colleagues who I once trusted,* α = .79). Moreover, reliability for the total scale (α = .82) was higher than α > .7, so, as preregistered, we employed the full scale for some analyses.

***Short Cognitive and Emotion Regulation Questionnaire (CERQ)***

Participants completed the 18-item short CERQ (Garnefski & Kraaij, 2006). They read a prompt: *People sometimes feel stressed or upset about experiences they have. When thinking about your involvement with investigations into child sexual abuse and exploitation (CSAE), how often do you have each thought or experience?* They then rated how often they experienced nine constructs with two items each on scales from 1 (*almost never*) to 7 (*almost always*). These items assess *self-blame* (α = .70), *other-blame* (α = .77), *rumination* (α = .72), *catastrophizing* (α = .84), *positive* *refocusing* (α = .63), *planning* (α = .70), *positive reappraisal* (α = .68), *putting into perspective* (α = .77), and *acceptance* (α = .66)*.* For positive reappraisal, we selected two items from the original CERQ that were not included in the short version, *I think that the situation also has its positive sides* and *I look for the positive sides to the matter*, as these refer to general positively about the situation rather than self-focused positively about personally feeling stronger, and hence may be more appropriate to the current situation where people view others suffering rather than personally caused most of the suffering.

***Behavioural Emotion Regulation Questionnaire (BERQ)***

Participants completed a short 10-item version of the BERQ (adapted from Kraaij & Garnefski, 2019) by responding to the prompt, *People sometimes take actions when they feel stressed or upset about experiences they have. When thinking about your involvement with investigations into child sexual abuse and exploitation* *(CSAE), how often do perform each behaviour?* Two items assess each of five constructs: *seeking distraction* (α = .77), *withdrawal* (α = .90), *actively approaching* (α = .57), *seeking social support* (α = .81), and *ignoring* (α = .75), on scales from 1 (*almost never*) to 7 (*almost always*). We created this short version by selecting the two top-loading items for each factor from Table 1 in Kraaij and Garnefski (2019).

***Brief Religious Coping Scale (RCOPE)***

The brief religious coping scale is a 14-item measure of religious coping, with two seven-item subscales reflecting positive and negative coping (Pargament et al., 1998). They will consider the following prompt: *Most people have some concept of God, religion, or spirituality, even if they do not personally believe in such things. We are interested in your thoughts however you understand such things. Some people report not being religious or spiritual, which is fine, but we still want to hear from you. Dealing with sexually explicit material involving children may lead some people to question or reinforce their beliefs about God, religion, or spirituality. We want to understand if, and how, such beliefs can influence wellbeing when dealing with child sexual abuse and exploitation. When thinking about your involvement with investigations into child sexual abuse and exploitation (CSAE), how often do you experience the following?*

Participants indicated how often they rely on 14 coping strategies on scales from 1 = *Almost never* to 7 = *Almost always* (note: we changed the scale to match the CERQ & BERQ). Again, we conducted a principal components analysis with oblimin rotation allowing for 500 iterations before convergence and 500 for rotation, retaining all factors with an eigenvalue greater than 1 (Kaiser, 1961) that are readily interpretable (Lee & Ashton, 1995). For each factor, we will retain all items loading .5 or greater on their respective factor, and no greater than .3 on any other factor. As expected, and consistent with Pargament and colleagues, we found evidence for two factors with eigenvalues greater than 1 (7.79, 2.27), accounting for 71.83% of variance in the scale, and all positive items loaded on *positive coping* (α = .95), which entails seeking comfort in religion (e.g., *Focused on religion to stop worrying about my problems*), and all negative items loading on *negative coping* (α = .86), which reflects anxiety about religious topics in relation to problems (e.g., *Questioned God’s love for me*)—with one exception. The negative item *decided the devil made this happen* instead loaded higher on the positive scale, so we included this item in the positive instead of negative scale.

***Patient Health Questionnaire-9 (PHQ-9)***

Participants completed a measure of depression, the PHQ-9, which asks participants how many times they experienced nine symptoms, such as, *little interest or pleasure in doing things*, over the past month, on scales where 0 = *not at all*, 1 = *several days*, 2 = *more than half the days*, 3 = *nearly every day* (Kroenke, Spitzer, & Williams, 2001). The scale also includes one difficulty item asking how difficult it has been to do work, take care of things at home, and get along with other people, on a scale from 1 = *Not difficult at all* – 4 = *very difficult*. We computed the mean across all items (α = .90). We also computed the sum across PHQ items to group people into five clinical categories.

***Generalized Anxiety Disorder-7 (GAD-7)***

Participants rated the frequency with which they experience seven symptoms of anxiety in the past month, such as *trouble relaxing*, on the same 4-point scale as the PHQ-9 (Spitzer, et al., 2006). We computed the mean across all items (α = .91). We also computed the sum across GAD-7 items to group people into four clinical categories.

***PTSD: The International Trauma Questionnaire (ITQ)***

We employed an 18-item self-report diagnostic measure of post-traumatic stress disorder (PTSD) and complex PTSD (CPTSD), as defined in the 11th version of the International Classification of Diseases (ICD-11). Participants considered a prompt: *Below are a number of problems that people sometimes report in response to traumatic or stressful life events, including investigating childhood sexual abuse and exploitation. When thinking about your involvement with investigations into child sexual abuse and exploitation (CSAE), how much have you been bothered by each problem over the past month?*

Participants reported the intensity of twelve symptoms, reflecting two superordinate clusters each with three subordinate clusters. Six PTSD items reflect *Reexperiencing*, *Avoidance*, and *Sense of Threat* (e.g., *Feeling jumpy or easily startled),* and six Disturbance in Self-organization items reflect *Affect Dysregulation*, *Negative Self-Concept*, and *Disturbances in Relationships* (e.g., *I feel numb or emotionally shut down*). In addition, three items for each superordinate cluster measured functional impairment (e.g., *How much have these symptoms affected your relationships or social life?*). Participants responded to all questions on scales from 0 (*not at all*) to 4 (*extremely*).

We use this measure to conduct both a main analysis and follow-up analysis, as well as diagnosis analysis. The main analysis assessed how predictors relate to overall average scores across all symptom and impairment items in the total scale (i.e., Complex PTSD; α = .94). A follow-up analysis examined how predictors separately relate to the average of all symptom and impairment items for the PTSD cluster (α = .90) and the Disturbance in Self-organization cluster (α = .93). We also computed the number of participants meeting the clinical criteria for a PTSD and C-PTSD diagnosis.

***Wellbeing: Schwartz Outcome Scale (SOS-10)***

Participants completed the SOS-10, a 10-item measure of wellbeing (e.g., *I have peace of mind*), on scales from 1 (*never*) to 7 *(all or nearly all of the time*, Blais et al., 1999). We included this measure as part of a growing movement to go beyond outcomes like anxiety and depression in assessment (Ogles et al., 2002). We computed the mean (α = .94).

***Resource Availability, Use, and Helpfulness***

Participants reported whether each of seven resources were available to them (0 = *no*, 1 = *yes*) and how often they use each resource on scales from 1 (*Never or Almost never*) to 7 (*Always or Almost always*): *Occupational Health Therapist,* *External/Self-referred counselling*, *Peer support program*, *Mental health or wellbeing days off*, *Clinical Supervision*, *Oscar Kilo* [Police Counselling Service], *Wellbeing of Investigators Toolkit* (see Table 6). We computed the mean (α = .66). Participants also reported how helpful they view each resource on scales from 1 (*Very unhelpful*) to 7 (*Very helpful*). We excluded participant responses when they selected 8 (*not applicable*). We computed the mean (α = .81).

***Barriers to Seeking Support***

We asked participants: *Thinking about possible barriers that might stop you from asking for mental health or wellbeing support at work, how much is each of the following a barrier for you?* They responded to seven items on scales from 1 (*Not at all*) to 7 (*A large amount*), such as *I feel pressured to seem strong in front of colleagues* and *Worried that getting support might undermine my job prospects* (see Table 7). We conducted a principal components analysis with oblimin rotation allowing for 500 iterations before convergence and 500 for rotation, retaining all factors with an eigenvalue greater than 1 (Kaiser, 1961) that are readily interpretable (Lee & Ashton, 1995). Although we anticipated a 2-factor solution, all items loaded on a single factor with an eigenvalue greater than 1 (4.55) accounting for 65% of variance in the scale. Therefore, we took the mean across all items (α = .91). We also asked an open-ended item: *Is there anything else you would like to add about possible barriers to seeking support (optional)?*

***Desired Resources***

Finally, we asked participants to check off resources they thought would be helpful and an open-ended item.

**Results**

**Clinical Prevalence**

First, we computed the clinical prevalence of depression, anxiety, and PTSD according to diagnostic criteria.

***Depression***

We computed the sum across PHQ-9 items to group people into five clinical categories (Kroenke, Spitzer, & Williams, 2001). Scores below 5 are categorized as minimal, 5-9 as mild, 10-4 as moderate, 15-19 as moderately severe, and 20-17 as severe (see Table 3). These authors argue that scores above 15, the moderately severe and severe categories, “usually signify the presence of major depression” (p. 611).

We found that 68 of 575, or 11.8% of our participants, reported symptoms within the past month that may qualify for major depression according to this measure. For comparison, one study of 172,751 middle-aged UK residents found that the prevalence of current depressive symptoms (on the related PHQ7 measure) was 1.5% among people with no mood disorder or major depressive episode (*N* = 98,539), 1.7% among people who had experienced a single major depressive episode (*N* = 7,927), 7.1% among people with recurrent moderate major depression (*N* = 15,013), 9.9% among people with recurrent severe major depression (*N* = 8,906) and 12.2% among people with bipolar disorder (*N* = 1,615; Smith et al., 2013). This suggests that the current population reports current depressive symptoms at similar rates to populations with bipolar disorder or recurrent severe major depression, and substantially above populations reporting single depressive episodes or even recurring moderate severity major depression.^[[3]](#footnote-3)^

**Table 3**

*Participants Scoring in Each Clinical Range on the Patient Health Questionnaire-9*

| Severity | *N* | Percent |
| --- | --- | --- |
| Minimal | 214 | 37.2 |
| Mild | 197 | 34.3 |
| Moderate | 96 | 16.7 |
| Moderately Severe | 45 | 7.8 |
| Severe | 23 | 4.0 |

***Generalized Anxiety***

We also computed the sum across GAD-7 items to group people into four clinical categories (Spitzer, et al., 2006). Scores below 5 are categorized as minimal, 5-9 as mild, 10-4 as moderate, and 15-21 as severe, (see Table 4). These authors argued that scores above 10, the moderate and severe categories, “represents a reasonable cut point for identifying cases of GAD” (p. 1096).

We found that 137 of 575, or 23.8% of our participants, reported symptoms within the past month that may qualify for generalized anxiety according to this measure. Comparisons are challenging, considering the wide range in global estimates of GAD from 3.8-25% (Remes et al., 2016), especially considering that rates tend to be higher in Western cultures like the UK, and among people with medical problems, such as 10.94% reported by Tulley & Cosh (2013). Rates may also be increasing over time, and highest among young people (Slee et al., 2021), wherese the average age in the current sample was close to 40. Moreover, rates vary depending on measure and timeframe, with one study estimating that 6.1% of participants reporting symptoms over past month but only 3.1% over three months (Haller, et al., 2014). Nonetheless, a plurality of estimates suggest that typical rates of GAD may typically vary between 2-10% in Western populations (Remes et al., 2016). For example, in a recent study of 30,446 UK residents, only 2.2% respondents qualified for GAD within the past year (Remes et al., 2018). Therefore, the estimate of 23.8% GAD in the current sample, including 8.5% in the severe category, suggests that anxiety rates may be substantially elevated in the current sample compared to the general population.

**Table 4**

*Participants Scoring in Each Clinical Range on the Generalized Anxiety Disorder-7*

| Severity | *N* | Percent |
| --- | --- | --- |
| Minimal | 237 | 41.2 |
| Mild | 201 | 35.0 |
| Moderate | 88 | 15.3 |
| Severe | 49 | 8.5 |

***PTSD and Complex PTSD***

We computed the number of participants meeting the clinical criteria for a PTSD and C-PTSD diagnosis. PTSD requires scoring a 2 or higher on at least one of the two symptoms of each of the three PTSD subscales, plus an average of 2 or higher on functional impairment for those scales. A diagnosis of Complex PTSD requires the same, plus scoring a 2 or higher on at least one of the two symptoms of each of the three Disturbance in Self-organization items and an average of 2 or higher on functional impairment for those scales.

We found that 18 participants (3.1%) met criteria for clinical levels of PTSD, and 29 (5.0%) met criteria for complex-PTSD, for a total of 8.1%. This result compares favorably to community samples: for example, Cyr and colleagues found a combined rate of 7.8% in a Canadian community sample, and Cloitre and colleagues (2018) found a rate of 18.3% in a representative UK community sample. Moreover, rates tend to be far higher in clinical samples. For example, Cloitre and colleagues (2018) found that 75.7% of a clinical sample met one or the other diagnostic criteria, and Murphy and colleagues (2020) found that 70.7% of a UK veteran sample seeking clinical help met diagnostic criteria. Therefore, the current sample looks closer to community samples than to clinical samples regarding PTSD.

**Table 5**

*Participants Scoring in Clinical Range on PTSD and Complex PTSD*

| Diagnosis | *N* | Percent |
| --- | --- | --- |
| Not Clinical | 528 | 91.9 |
| PTSD | 18 | 3.1 |
| Complex PTSD | 29 | 5.0 |

**Wellbeing**

Although the SOS does not have clinical cut-off values, we nonetheless considered how mean SOS responses in the current sample, *M* = 4.69, *SD* = 1.11 compare to other samples. We examined SOS values in previous research.^[[4]](#footnote-4)^ Typical populations tend to score somewhat higher than the current sample: Young and colleagues (2003) found that two samples of American undergraduates averaged *M*_1_ = 5.73; *M*_2_ = 5.53 and Richards and colleagues (2003) found that a sample of mental health professionals averaged *M* = 5.28. Conversely, clinical and troubled samples score approximately similar to or lower than the current sample: Young and colleagues (2003) found that counselling centre clients averaged M_1_ = 4.9, and a second sample averaged M_2_ = 4.47 on intake. Blais (1999) samples patients with severe psychiatric disorders and found a mean of 3.9. Although caution should be exerted when comparing across different samples, overall these results suggest the current sample may score somewhat lower in well-being than typical samples, and more in line with samples of troubled people.

**Regression Analysis**

Next, we conducted a series of linear multiple regressions from a series of (standardized) theoretical predictors to each of four outcome measures: Anxiety, Depression, PTSD, and Wellbeing (Tabachnick & Fidell, 2001). Each analysis will examine the unique predictive relationship of each set of theoretical predictors (at step 2) controlling for demographics (age, gender, officer versus staff, career length, role length, relationship status, parent status, caregiver status, therapy experience, and religiosity) at step 1. The sets of regression predictors will be as follows: the three risk and protective factors, the two facets of the RCOPE, the three facets of the MIES, the nine facets of the CERQ, the five facets of the BERQ. We will conduct follow-up analyses examining whether findings hold when breaking down PTSD into two broad factors and six individual factors.

***Demographic Predictors***

We entered demographic predictors at step 1 of each analysis. The majority of these variables failed to predict significant variance in any outcomes, with two primary exceptions (see Tables 9-13). First, therapy experience: people who reported attending therapy at any point in their lives, whether previously or currently (about a third of the sample) tended to report higher rates of moral injury, depression, anxiety, and PTSD, and reduced wellbeing than people who reported never having attended therapy (about two thirds of the sample). Second, religiosity: people who reported greater religious experience likewise reported higher rates of moral injury, depression, and PTSD, and reduced wellbeing, with inconsistently higher rates of anxiety. It may be that religious people particularly struggle with CSAE material. One final exception: parents occasionally reported lower rates of PSTD, but this effect was weak and inconsistent. Importantly, all findings below refer to the unique effects of each predictor above and beyond demographic factors.

***Risk and Support Factors***

The principal components analysis revealed three factors that only partially aligned with preregistered predictions, but nonetheless appeared to reflect three theoretically sensible factors somewhat similar to expectations. These corresponded to two risk factors—contact with victims and perpetrators, and exposure to CSAE material—and one protective factor: feeling successful and supported. We predicted a priori that regressions on each DV, using all three factors as simultaneous predictors controlling for demographic variables, will show that both risk factors will predict increased moral injury, PTSD, anxiety, and depression, and reduced wellbeing, whereas both success and support protective factors will predict reduced moral injury, PTSD, anxiety, and depression, and increased wellbeing.

Results partially confirmed this prediction (see Table 9). Exposure to CSAE material uniquely predicted moral injury but no other factor, whereas contact with victims and perpetrators uniquely predicted depression, anxiety, and PTSD but no other factors. Meanwhile, feeling successful and supported predicted all factors: reduced moral injury, depression, anxiety, and PTSD, and increased wellbeing. Moreover, the effect size for feeling successful and supported was substantially larger than either risk factor, more than double the size. Hence, results point to the powerful importance of feeling supported and engaged in the workplace and in the home for managing the stresses of dealing with CSAE material. For example, people who reported higher levels of *Feeling supported by my supervisor or team*, *Feeling like my voice is heard and I have input into decisions that affect me,* and *Feeling my work is valuable and meaningful* generally reported substantially better outcomes than people who reported lower levels of these perceptions.

***Moral Injury***

Consistent with Bryan and colleagues (2016), we found evidence for three separate factors underlying moral injury, which we treated as simultaneous predictors of each key outcome measure controlling for demographic variables (see Table 10). We anticipated replicating the findings of Bryan and colleagues that regressions on each DV, using all three factors as simultaneous predictors controlling for demographic variables, will show that all three measures will uniquely predict increased PTSD, anxiety, and depression, and reduced wellbeing. However, results instead showed that betrayal was by far the most important factor: betrayal predicted increased PTSD, anxiety, and depression, and reduced wellbeing controlling for the other two factors and demographics. Conversely, the unique effects of transgression-self and transgressions-other were not significant.

Hence, we found mixed support for the pattern observed by Bryan and colleagues. We predicted that transgressions-other and betrayal would predict PTSD more than transgressions-self—we confirmed this pattern for betrayal but not transgressions-other. Nor did we find support for Bryan and colleagues’ finding that transgression-other negatively predicted depression (which we preregistered that we did not expect to find). However, we also did not find support for our prediction that transgression-other would positively predict depression, in line with Nash and colleagues (2013). In sum, it seems that in this population, feelings of moral betrayal by colleagues were by far the most important factor driving outcomes. This pattern is all the more striking considering that participants deal directly with morally compromising information and individuals, risking potential for both transgression-self and other to matter.

***Cognitive and Emotion Regulation***

We calculated reports of each of the cognitive and emotion regulation strategies described by Garnefski and Kraaij (2006), treating each as simultaneous predictors of each key outcome measure controlling for demographic variables (see Table 11). We anticipated replicating their findings that regressions on each DV: that self-blame, rumination, and catastrophizing will predict increased depression and anxiety, whereas positive reappraisal will predict reduced depression and anxiety. We expected a similar pattern of prediction for moral injury and PTSD, and the opposite pattern for wellbeing.

Results largely corroborated these predicted patterns: Self-blame and rumination each uniquely predicted depression and anxiety, as well as moral injury, PTSD, and reduced well-being. Likewise, positive reappraisal predicted reduced depression and increased wellbeing, though it failed to significantly predict moral injury, anxiety, or PTSD.

In addition, despite no specific predictions, positive refocusing predicted reduced depression, anxiety, PTSD, and increased wellbeing, though not moral injury, and planning predicted reduced moral injury and PTSD, and increased wellbeing. Intriguingly, putting into perspective predicted increased depression and reduced wellbeing, suggesting that strategies like ‘*I tell myself there are worse things in life*’ can actually backfire by reminding one of those worse things. Likewise, catastrophizing appeared particularly toxic, as it was associated with increased moral injury, depression, anxiety, PTSD, and reduced well-being. Other-blame uniquely predicted increased moral injury, but no other outcome. Finally, acceptance failed to predict any outcome.

In sum, we largely replicated the patterns described by Garnefski and Kraaij, and found in general that self-blame, rumination, putting into perspective, catastrophizing, and other-blame predicted worse outcomes, whereas positive reappraisal, positive refocusing, and planning predicted better outcomes. These findings may suggest intervention strategies useful for assisting police officers and staff deal with CSAE material, as well as strategies to avoid.

***Behavioural Emotion Regulation Questionnaire (BERQ)***

We also calculated reports of each of the behavioral emotion regulation strategies described by Kraaij and Garnefski (2019), treating each as simultaneous predictors of each key outcome measure controlling for demographic variables (see Table 12). We anticipated replicating their findings that withdrawal and ignoring would predict increased depression and anxiety, whereas seeking distraction and social support would predict reduced depression and anxiety. We expected a similar pattern for moral injury and PTSD, and the opposite pattern for wellbeing.

Results largely corroborated expectations. Withdrawal and ignoring each uniquely predicted increased moral injury, depression, anxiety, PTSD, and reduced well-being. Conversely, seeking distraction uniquely predicted reduced depression, anxiety, and PTSD, and increased well-being, whereas social support uniquely predicted reduced depression and PTSD, and increased well-being, but not anxiety. Neither seeking distraction nor social support predicted moral injury, however. Meanwhile, approach and planning failed to uniquely predict any outcomes, except approach predicted increased wellbeing.

In sum, we largely replicated the patterns described by Garnefski and Kraaij, finding in general that withdrawal and ignoring were associated with worse outcomes, whereas seeking distraction and social support were generally associated with better outcomes. Again, these findings may suggest intervention strategies useful for assisting police officers and staff deal with CSAE material, as well as strategies to avoid.

***Religious Coping***

A principal components analysis largely corroborated the two-factor structure of positive and negative religious coping, with one item shifted from the negative to positive scale. We predicted that, consistent with other work, positive religious coping would be weakly negatively or unassociated with depression, anxiety, and PTSD, but positively associated with wellbeing, whereas negative religious coping would be positively associated with PTSD, anxiety, and depression, but weakly negatively or unassociated with wellbeing (Pargament et al., 2011; Currier et al., 2015b, 2017).

Results partially supported hypotheses (see Table 13). As expected, negative religious coping predicted increased depression, anxiety, and PTSD, as well as, and did not predict wellbeing. Meanwhile, as expected, positive religious coping failed to predict moral injury, depression, anxiety, and PTSD. However, inconsistent with expectations, positive religious coping also failed to predict increased wellbeing. Hence, people who agreed with statements like they *Questioned God’s love for me* generally reported worse outcomes, whereas people who agreed with statements like they *Focused on religion to stop worrying about my problems* did not appear to report better outcomes. That said, these findings controlled for religiosity, which remained a significant predictor. It may be that positive or negative religious coping may mediate the influence of religiosity on some outcomes.

Occupational Health Therapist; external/self-referred counselling; peer-support program; mental health or wellbeing days off; clinical supervision, Oscar Kilo, Wellbeing of Investigators Toolkit, other

**Table 6**

*Resource Access, Usage, and Helpfulness*

|  | Provided (0=*no*, 1=*yes*) | | Used (1-7) | | Helpful (1-7) | |
| --- | --- | --- | --- | --- | --- | --- |
| Resource | *N* | Percent | *M* | *SD* | *M* | *SD* |
| Occupational Health Therapist | 470 | 81.7 | 2.09 | 1.42 | 3.91 | 1.91 |
| External/self-referred counselling | 341 | 59.3 | 1.95 | 1.47 | 4.67 | 2.09 |
| Peer-support program | 271 | 47.1 | 1.31 | 0.95 | 3.49 | 2.13 |
| Mental health or wellbeing days off | 113 | 19.7 | 1.34 | 1.02 | 3.95 | 2.43 |
| Clinical supervision | 29 | 5 | 1.17 | 0.86 | 2.29 | 1.89 |
| Oscar Kilo | 231 | 40.2 | 1.18 | 0.76 | 2.76 | 1.97 |
| Wellbeing of Investigators Toolkit | 152 | 26.4 | 1.23 | 0.80 | 3.09 | 1.96 |
| Other | 73 | 12.7 | 2.09 | 1.42 | 3.91 | 1.91 |

**Table 7**

*Barriers to Support*

| Barrier | *M* | *SD* |
| --- | --- | --- |
| I feel pressured to seem strong in front of colleagues | 3.95 | 2.108 |
| Workplace culture where weakness seems not allowed | 3.15 | 2.045 |
| Wanting to seem capable of handling stress and performing well | 4.63 | 2.039 |
| It seems like everyone else can cope with this job | 3.73 | 2.090 |
| Worried that getting support might undermine my job prospects | 3.53 | 2.323 |
| I'm not convinced that seeking support will help and it might even cause problems | 3.27 | 2.147 |
| I don't trust my organization to keep my support confidential | 3.21 | 2.305 |

**Table 8**

*Desired Resources*

| Resource | *N* | Percent |
| --- | --- | --- |
| Monthly group sessions with team | 198 | 34.4 |
| Monthly wellness check-in | 275 | 47.8 |
| 24/7 access to support | 276 | 48.0 |
| Limiting daily exposure to child sexual abuse materials | 164 | 28.5 |
| Separating viewing online CSAE tasks and interviewing victims and/or perpetrators | 73 | 12.7 |
| A workplace culture that explicitly values and prioritizes the emotional health and wellbeing of the workforce | 348 | 60.5 |
| A wellness room | 208 | 36.2 |
| Informal peer support | 161 | 28.0 |
| Wellness events (workshops, training) | 220 | 28.3 |
| Wellness plans | 125 | 21.7 |
| Social activities | 341 | 59.3 |
| Mindfulness sessions | 235 | 40.9 |
| Clinical supervision | 128 | 22.3 |
| Self-referral to funded counselling (separate from work) | 288 | 50.1 |
| Other | 73 | 12.7 |

**Table 9**

*Exposure to Child Sexual Abuse and Exploitation (CSAE) Material, Contact with Victims and Perpetrators, and Job Success and Support Predict Moral Injury, Depression, Generalized Anxiety, Complex PTSD, and Wellbeing Beyond Demographics*

| **Predictors** |  | **Moral Injury** | | |  | **Depression** | | |  | **Generalized Anxiety** | | |  | **Complex PTSD** | | |  | **Wellbeing** | | |
| --- | --- | --- | --- | --- | --- | --- | --- | --- | --- | --- | --- | --- | --- | --- | --- | --- | --- | --- | --- | --- |
|  |  | **β** | ***t*** | ***p*** |  | **β** | ***t*** | ***p*** |  | **β** | ***t*** | ***p*** |  | **β** | ***t*** | ***p*** |  | **β** | ***t*** | ***p*** |
| **Step 1** |  |  |  |  |  |  |  |  |  |  |  |  |  |  |  |  |  |  |  |  |
| Age |  | -0.03 | -0.44 | .658 |  | -0.08 | -1.28 | .200 |  | -0.10 | -1.51 | .131 |  | -0.06 | -0.99 | .323 |  | 0.04 | 0.70 | .484 |
| Gender |  | -0.01 | -0.28 | .780 |  | -0.04 | -1.00 | .316 |  | 0.04 | 0.97 | .331 |  | -0.05 | -1.07 | .284 |  | 0.05 | 1.10 | .271 |
| Officer vs Staff  (1=*officer*, 2=*staff*) |  | -0.08 | -1.60 | .109 |  | -0.05 | -0.98 | .325 |  | -0.04 | -0.72 | .472 |  | 0.01 | 0.11 | .911 |  | -0.05 | -1.01 | .314 |
| Career Length |  | -0.01 | -0.13 | .896 |  | 0.04 | 0.55 | .583 |  | 0.06 | 0.81 | .420 |  | -0.01 | -0.10 | .923 |  | -0.01 | -0.12 | .905 |
| Role Length |  | 0.08 | 1.57 | .116 |  | 0.02 | 0.34 | .735 |  | 0.00 | 0.09 | .925 |  | 0.06 | 1.32 | .188 |  | -0.02 | -0.40 | .687 |
| Relationship Status  (1 = *partnered*,  0 = *not partnered*) |  | 0.04 | 0.88 | .381 |  | -0.05 | -1.15 | .251 |  | -0.07 | -1.37 | .173 |  | -0.07 | -1.53 | .128 |  | 0.05 | 0.96 | .337 |
| Parent of <18  (1=*yes*, 0=*no*) |  | -0.04 | -0.81 | .417 |  | -0.04 | -0.86 | .388 |  | -0.04 | -0.87 | .386 |  | **-0.10** | **-1.98** | **.048** |  | 0.06 | 1.24 | .215 |
| Caregiver >18  (1=*yes*, 0=*no*) |  | 0.01 | 0.15 | .883 |  | -0.03 | -0.68 | .500 |  | 0.00 | -0.04 | .965 |  | 0.00 | 0.05 | .959 |  | 0.04 | 0.87 | .387 |
| Therapy Experience  (1=*yes*, 0=*no*) |  | **0.09** | **2.10** | **.036** |  | **0.14** | **3.30** | **.001** |  | **0.10** | **2.41** | **.016** |  | **0.19** | **4.50** | **<.001** |  | **-0.15** | **-3.41** | **.001** |
| Religiosity |  | **0.11** | **2.64** | **.008** |  | **0.13** | **3.13** | **.002** |  | 0.08 | 1.97 | .050 |  | **0.09** | **2.13** | **.033** |  | **-0.09** | **-2.01** | **.045** |
| **Step 2** |  |  |  |  |  |  |  |  |  |  |  |  |  |  |  |  |  |  |  |  |
| Exposure to CSAE Material |  | **0.15** | **3.60** | **<.001** |  | 0.03 | 0.70 | .487 |  | 0.00 | 0.02 | .985 |  | 0.07 | 1.81 | .071 |  | -0.07 | -1.86 | .063 |
| Contact with Victims & Perpetrators |  | 0.09 | 1.81 | .071 |  | **0.19** | **4.03** | **<.001** |  | **0.12** | **2.50** | **.013** |  | **0.21** | **4.59** | **<.001** |  | -0.06 | -1.26 | .210 |
| Job Success & Support |  | **-0.34** | **-8.56** | **<.001** |  | **-0.44** | **-11.87** | **<.001** |  | **-0.39** | **-9.87** | **<.001** |  | **-0.49** | **-13.69** | **<.001** |  | **0.54** | **15.01** | **<.001** |

*Note:* bold indicates significance.

**Table 10**

*Moral Injury Subscales: Transgression-Self, Transgression-Other, and Betrayal, Predict Depression, Generalized Anxiety, Complex PTSD, and Wellbeing Beyond Demographics*

| **Predictors** |  | **Depression** | | |  | **Generalized Anxiety** | | |  | **Complex PTSD** | | |  | **Wellbeing** | | |
| --- | --- | --- | --- | --- | --- | --- | --- | --- | --- | --- | --- | --- | --- | --- | --- | --- |
|  |  | **β** | ***t*** | ***p*** |  | **β** | ***t*** | ***p*** |  | **β** | ***t*** | ***p*** |  | **β** | ***t*** | ***p*** |
| **Step 1** |  |  |  |  |  |  |  |  |  |  |  |  |  |  |  |  |
| Age |  | -0.08 | -1.32 | .189 |  | -0.10 | -1.59 | .113 |  | -0.07 | -1.05 | .294 |  | 0.06 | 0.94 | .348 |
| Gender |  | -0.04 | -1.03 | .302 |  | 0.04 | 0.93 | .351 |  | -0.05 | -1.14 | .255 |  | 0.05 | 1.21 | .226 |
| Officer vs Staff  (1=*officer*, 2=*staff*) |  | -0.05 | -1.02 | .310 |  | -0.04 | -0.76 | .450 |  | 0.00 | 0.04 | .966 |  | -0.05 | -0.94 | .348 |
| Career Length |  | 0.04 | 0.58 | .561 |  | 0.06 | 0.88 | .378 |  | 0.00 | -0.03 | .973 |  | -0.02 | -0.34 | .732 |
| Role Length |  | 0.02 | 0.32 | .746 |  | 0.00 | 0.08 | .936 |  | 0.06 | 1.28 | .200 |  | -0.02 | -0.38 | .703 |
| Relationship Status  (1 = *partnered*,  0 = *not partnered*) |  | -0.05 | -1.11 | .268 |  | -0.06 | -1.29 | .198 |  | -0.07 | -1.45 | .147 |  | 0.04 | 0.76 | .447 |
| Parent of <18  (1=*yes*, 0=*no*) |  | -0.04 | -0.81 | .419 |  | -0.04 | -0.78 | .438 |  | -0.09 | -1.87 | .063 |  | 0.05 | 1.01 | .315 |
| Caregiver >18  (1=*yes*, 0=*no*) |  | -0.03 | -0.69 | .493 |  | 0.00 | -0.06 | .952 |  | 0.00 | 0.03 | .980 |  | 0.04 | 0.91 | .363 |
| Therapy Experience  (1=*yes*, 0=*no*) |  | **0.14** | **3.33** | **.001** |  | **0.10** | **2.44** | **.015** |  | **0.19** | **4.56** | **<.001** |  | **-0.15** | **-3.51** | **<.001** |
| Religiosity |  | **0.13** | **3.15** | **.002** |  | **0.08** | **2.00** | **.046** |  | **0.09** | **2.18** | **.030** |  | **-0.09** | **-2.10** | **.036** |
| **Step 2** |  |  |  |  |  |  |  |  |  |  |  |  |  |  |  |  |
| Transgression-Self |  | 0.04 | 0.99 | .324 |  | 0.06 | 1.42 | .156 |  | 0.08 | 1.88 | .060 |  | -0.05 | -1.29 | .198 |
| Transgression-Other |  | 0.00 | -0.08 | .939 |  | -0.02 | -0.49 | .621 |  | 0.07 | 1.66 | .097 |  | -0.07 | -1.52 | .128 |
| Betrayal |  | **0.34** | **7.44** | **<.001** |  | **0.33** | **7.06** | **<.001** |  | **0.35** | **8.00** | **<.001** |  | **-0.28** | **-6.12** | **<.001** |

*Note:* bold indicates significance.

**Table 11**

*Cognitive and Emotional Regulation Subscales: Self-blame, Acceptance, Rumination, Positive Refocusing, Planning, Positive Reappraisal, Putting into Perspective, Catastrophizing, and Other-blame Predict Moral Injury, Depression, Generalized Anxiety, Complex PTSD, and Wellbeing Beyond Demographics*

| **Predictors** |  | **Moral Injury** | | |  | **Depression** | | |  | **Generalized Anxiety** | | |  | **Complex PTSD** | | |  | **Wellbeing** | | |
| --- | --- | --- | --- | --- | --- | --- | --- | --- | --- | --- | --- | --- | --- | --- | --- | --- | --- | --- | --- | --- |
|  |  | **β** | ***t*** | ***p*** |  | **β** | ***t*** | ***p*** |  | **β** | ***t*** | ***p*** |  | **β** | ***t*** | ***p*** |  | **β** | ***t*** | ***p*** |
| **Step 1** |  |  |  |  |  |  |  |  |  |  |  |  |  |  |  |  |  |  |  |  |
| Age |  | -0.02 | -0.33 | .742 |  | -0.07 | -1.07 | .283 |  | -0.08 | -1.31 | .190 |  | -0.05 | -0.79 | .432 |  | 0.02 | 0.36 | .722 |
| Gender |  | -0.01 | -0.28 | .776 |  | -0.05 | -1.06 | .290 |  | 0.04 | 0.87 | .386 |  | -0.05 | -1.18 | .240 |  | 0.05 | 1.13 | .261 |
| Officer vs Staff  (1=*officer*, 2=*staff*) |  | -0.07 | -1.44 | .151 |  | -0.05 | -0.96 | .339 |  | -0.03 | -0.71 | .478 |  | 0.01 | 0.11 | .916 |  | -0.05 | -1.07 | .284 |
| Career Length |  | -0.01 | -0.12 | .908 |  | 0.03 | 0.45 | .655 |  | 0.05 | 0.71 | .477 |  | -0.01 | -0.20 | .842 |  | 0.01 | 0.07 | .944 |
| Role Length |  | 0.08 | 1.71 | .089 |  | 0.02 | 0.38 | .706 |  | 0.01 | 0.15 | .882 |  | 0.06 | 1.34 | .181 |  | -0.02 | -0.46 | .643 |
| Relationship Status  (1 = *partnered*,  0 = *not partnered*) |  | 0.04 | 0.77 | .440 |  | -0.06 | -1.23 | .220 |  | -0.07 | -1.50 | .134 |  | -0.08 | -1.59 | .112 |  | 0.05 | 1.09 | .275 |
| Parent of <18  (1=*yes*, 0=*no*) |  | -0.04 | -0.78 | .434 |  | -0.04 | -0.85 | .398 |  | -0.04 | -0.80 | .421 |  | -0.09 | -1.95 | .051 |  | 0.06 | 1.23 | .219 |
| Caregiver >18  (1=*yes*, 0=*no*) |  | 0.00 | -0.02 | .988 |  | -0.04 | -0.84 | .401 |  | -0.01 | -0.21 | .831 |  | 0.00 | -0.09 | .928 |  | 0.05 | 1.16 | .246 |
| Therapy Experience  (1=*yes*, 0=*no*) |  | **0.08** | **1.98** | **.048** |  | **0.14** | **3.28** | **.001** |  | **0.10** | **2.39** | **.017** |  | **0.19** | **4.52** | **<.001** |  | **-0.14** | **-3.38** | **.001** |
| Religiosity |  | **0.11** | **2.50** | **.013** |  | **0.13** | **3.05** | **.002** |  | 0.08 | 1.90 | .058 |  | **0.09** | **2.03** | **.043** |  | **-0.08** | **-1.94** | **.052** |
| **Step 2** |  |  |  |  |  |  |  |  |  |  |  |  |  |  |  |  |  |  |  |  |
| Self-Blame |  | **0.15** | **3.54** | **<.001** |  | **0.14** | **3.45** | **.001** |  | **0.09** | **2.19** | **.029** |  | **0.16** | **4.11** | **<.001** |  | **-0.18** | **-4.23** | **<.001** |
| Acceptance |  | 0.01 | 0.28 | .783 |  | 0.03 | 0.63 | .526 |  | 0.02 | 0.45 | .653 |  | 0.02 | 0.53 | .594 |  | 0.03 | 0.65 | .517 |
| Rumination |  | **0.27** | **4.93** | **<.001** |  | **0.18** | **3.48** | **.001** |  | **0.20** | **3.71** | **<.001** |  | **0.25** | **5.07** | **<.001** |  | **-0.15** | **-2.76** | **.006** |
| Positive Refocusing |  | -0.08 | -1.92 | .055 |  | **-0.17** | **-4.16** | **<.001** |  | **-0.12** | **-3.04** | **.003** |  | **-0.18** | **-4.82** | **<.001** |  | **0.19** | **4.56** | **<.001** |
| Planning |  | **-0.10** | **-2.32** | **.021** |  | -0.02 | -0.51 | .610 |  | -0.02 | -0.57 | .569 |  | **-0.07** | **-1.96** | **.050** |  | **0.10** | **2.38** | **.018** |
| Positive Reappraisal |  | -0.04 | -0.80 | .424 |  | **-0.12** | **-2.70** | **.007** |  | -0.08 | -1.70 | .089 |  | -0.04 | -0.99 | .325 |  | **0.10** | **2.22** | **.027** |
| Putting into Perspective |  | 0.04 | 0.98 | .329 |  | **0.09** | **2.05** | **.040** |  | 0.07 | 1.63 | .104 |  | 0.07 | 1.62 | .107 |  | **-0.09** | **-2.07** | **.039** |
| Catastrophizing |  | **0.11** | **2.10** | **.036** |  | **0.28** | **5.50** | **<.001** |  | **0.30** | **5.73** | **<.001** |  | **0.29** | **5.99** | **<.001** |  | **-0.24** | **-4.56** | **<.001** |
| Other-Blame |  | **0.17** | **4.07** | **<.001** |  | -0.01 | -0.16 | .870 |  | 0.03 | 0.63 | .528 |  | 0.05 | 1.43 | .152 |  | -0.05 | -1.33 | .183 |

*Note:* bold indicates significance.

**Table 12**

*Behavioral Coping Subscales Seeking Distraction, Withdrawal, Approach, Planning, Social Support, and Ignoring Predict Moral Injury, Depression, Generalized Anxiety, Complex PTSD, and Wellbeing Beyond Demographics*

| **Predictors** |  | **Moral Injury** | | |  | **Depression** | | |  | **Generalized Anxiety** | | |  | **Complex PTSD** | | |  | **Wellbeing** | | |
| --- | --- | --- | --- | --- | --- | --- | --- | --- | --- | --- | --- | --- | --- | --- | --- | --- | --- | --- | --- | --- |
|  |  | **β** | ***t*** | ***p*** |  | **β** | ***t*** | ***p*** |  | **β** | ***t*** | ***p*** |  | **β** | ***t*** | ***p*** |  | **β** | ***t*** | ***p*** |
| **Step 1** |  |  |  |  |  |  |  |  |  |  |  |  |  |  |  |  |  |  |  |  |
| Age |  | -0.02 | -0.35 | .727 |  | -0.07 | -1.10 | .274 |  | -0.09 | -1.33 | .185 |  | -0.05 | -0.81 | .416 |  | 0.02 | 0.38 | .702 |
| Gender |  | -0.01 | -0.26 | .796 |  | -0.04 | -1.03 | .304 |  | 0.04 | 0.89 | .375 |  | -0.05 | -1.14 | .255 |  | 0.05 | 1.09 | .277 |
| Officer vs Staff  (1=*officer*, 2=*staff*) |  | -0.07 | -1.46 | .144 |  | -0.05 | -0.98 | .326 |  | -0.04 | -0.73 | .466 |  | 0.00 | 0.07 | .942 |  | -0.05 | -1.04 | .300 |
| Career Length |  | -0.01 | -0.11 | .916 |  | 0.03 | 0.46 | .647 |  | 0.05 | 0.72 | .472 |  | -0.01 | -0.18 | .854 |  | 0.00 | 0.06 | .955 |
| Role Length |  | 0.08 | 1.66 | .097 |  | 0.02 | 0.33 | .742 |  | 0.01 | 0.11 | .909 |  | 0.06 | 1.28 | .202 |  | -0.02 | -0.40 | .687 |
| Relationship Status  (1 = *partnered*,  0 = *not partnered*) |  | 0.03 | 0.72 | .474 |  | -0.06 | -1.29 | .198 |  | -0.07 | -1.55 | .123 |  | -0.08 | -1.66 | .097 |  | 0.06 | 1.17 | .243 |
| Parent of <18  (1=*yes*, 0=*no*) |  | -0.04 | -0.75 | .452 |  | -0.04 | -0.81 | .418 |  | -0.04 | -0.78 | .435 |  | -0.09 | -1.91 | .056 |  | 0.06 | 1.19 | .235 |
| Caregiver >18  (1=*yes*, 0=*no*) |  | 0.00 | 0.00 | .998 |  | -0.04 | -0.83 | .409 |  | -0.01 | -0.20 | .839 |  | 0.00 | -0.07 | .941 |  | 0.05 | 1.14 | .254 |
| Therapy Experience  (1=*yes*, 0=*no*) |  | 0.08 | 1.95 | .052 |  | **0.14** | **3.25** | **.001** |  | **0.10** | **2.36** | **.018** |  | **0.19** | **4.47** | **<.001** |  | **-0.14** | **-3.33** | **.001** |
| Religiosity |  | **0.11** | **2.59** | **.010** |  | **0.13** | **3.15** | **.002** |  | 0.08 | 1.97 | .050 |  | 0.09 | 2.14 | .033 |  | **-0.09** | **-2.06** | **.040** |
| **Step 2** |  |  |  |  |  |  |  |  |  |  |  |  |  |  |  |  |  |  |  |  |
| Seeking Distraction |  | 0.04 | 0.87 | .387 |  | **-0.12** | **-3.32** | **.001** |  | **-0.09** | **-2.19** | **.029** |  | **-0.08** | **-2.29** | **.023** |  | **0.15** | **4.07** | **<.001** |
| Withdrawal |  | **0.25** | **5.04** | **<.001** |  | **0.50** | **12.20** | **<.001** |  | **0.43** | **9.67** | **<.001** |  | **0.52** | **13.13** | **<.001** |  | **-0.46** | **-10.93** | **<.001** |
| Approach |  | -0.09 | -1.88 | .061 |  | 0.00 | -0.03 | .978 |  | -0.02 | -0.58 | .565 |  | -0.05 | -1.40 | .161 |  | **0.09** | **2.38** | **.018** |
| Planning |  | -0.02 | -0.39 | .694 |  | 0.01 | 0.40 | .691 |  | 0.03 | 0.86 | .389 |  | 0.01 | 0.34 | .737 |  | 0.03 | 0.76 | .447 |
| Social Support |  | 0.06 | 1.36 | .173 |  | **-0.10** | **-2.48** | **.013** |  | -0.03 | -0.80 | .422 |  | **-0.09** | **-2.31** | **.021** |  | **0.13** | **3.34** | **.001** |
| Ignoring |  | **0.12** | **2.31** | **.021** |  | **0.16** | **3.68** | **<.001** |  | **0.14** | **3.02** | **.003** |  | **0.16** | **3.89** | **<.001** |  | **-0.11** | **-2.39** | **.017** |

*Note:* bold indicates significance.

**Table 13**

*Positive and Negative Religious Coping Predicts Moral Injury, Depression, Generalized Anxiety, Complex PTSD, and Wellbeing Beyond Demographics*

| **Predictors** |  | **Moral Injury** | | |  | **Depression** | | |  | **Generalized Anxiety** | | |  | **Complex PTSD** | | |  | **Wellbeing** | | |
| --- | --- | --- | --- | --- | --- | --- | --- | --- | --- | --- | --- | --- | --- | --- | --- | --- | --- | --- | --- | --- |
|  |  | **β** | ***t*** | ***p*** |  | **β** | ***t*** | ***p*** |  | **β** | ***t*** | ***p*** |  | **β** | ***t*** | ***p*** |  | **β** | ***t*** | ***p*** |
| **Step 1** |  |  |  |  |  |  |  |  |  |  |  |  |  |  |  |  |  |  |  |  |
| Age |  | -0.06 | -0.91 | .365 |  | -0.08 | -1.17 | .242 |  | -0.08 | -1.31 | .190 |  | -0.05 | -0.78 | .439 |  | 0.02 | 0.30 | .767 |
| Gender |  | -0.01 | -0.15 | .882 |  | -0.05 | -1.06 | .292 |  | 0.04 | 0.91 | .364 |  | -0.05 | -1.17 | .242 |  | 0.05 | 1.16 | .247 |
| Officer vs Staff  (1=*officer*, 2=*staff*) |  | -0.07 | -1.48 | .139 |  | -0.05 | -1.01 | .313 |  | -0.04 | -0.74 | .458 |  | 0.00 | 0.05 | .957 |  | -0.05 | -0.98 | .327 |
| Career Length |  | 0.02 | 0.24 | .814 |  | 0.04 | 0.50 | .619 |  | 0.05 | 0.69 | .490 |  | -0.01 | -0.21 | .833 |  | 0.01 | 0.19 | .852 |
| Role Length |  | 0.08 | 1.64 | .101 |  | 0.02 | 0.34 | .738 |  | 0.00 | 0.10 | .920 |  | 0.06 | 1.30 | .193 |  | -0.02 | -0.42 | .676 |
| Relationship Status  (1 = *partnered*,  0 = *not partnered*) |  | 0.05 | 1.06 | .288 |  | -0.06 | -1.21 | .227 |  | -0.07 | -1.46 | .146 |  | -0.08 | -1.65 | .101 |  | 0.05 | 1.09 | .278 |
| Parent of <18  (1=*yes*, 0=*no*) |  | -0.04 | -0.79 | .427 |  | -0.04 | -0.84 | .401 |  | -0.04 | -0.86 | .392 |  | -0.09 | -1.94 | .053 |  | 0.06 | 1.28 | .202 |
| Caregiver >18  (1=*yes*, 0=*no*) |  | 0.01 | 0.16 | .876 |  | -0.03 | -0.67 | .505 |  | 0.00 | -0.03 | .976 |  | 0.00 | 0.06 | .951 |  | 0.04 | 0.85 | .394 |
| Therapy Experience  (1=*yes*, 0=*no*) |  | **0.09** | **2.09** | **.037** |  | **0.14** | **3.27** | **.001** |  | **0.10** | **2.37** | **.018** |  | **0.19** | **4.46** | **<.001** |  | **-0.15** | **-3.39** | **.001** |
| Religiosity |  | **0.11** | **2.68** | **.008** |  | **0.13** | **3.13** | **.002** |  | 0.08 | 1.95 | .052 |  | **0.09** | **2.13** | **.034** |  | -0.08 | -1.94 | .053 |
| **Step 2** |  |  |  |  |  |  |  |  |  |  |  |  |  |  |  |  |  |  |  |  |
| Positive Religious Coping |  | 0.05 | 0.87 | .383 |  | -0.03 | -0.47 | .640 |  | -0.06 | -0.92 | .358 |  | 0.01 | 0.09 | .928 |  | 0.02 | 0.35 | .728 |
| Negative Religious Coping |  | **0.16** | **3.32** | **.001** |  | **0.17** | **3.54** | **<.001** |  | **0.20** | **4.05** | **<.001** |  | **0.19** | **3.95** | **<.001** |  | -0.09 | -1.85 | .065 |

*Note:* bold indicates significance.

**References**

Blais, M. A., Lenderking, W. R. Baer, L., de Lorell, A., Peets, K., Leahy, L., et al. (1999). Development and initial validation of a brief mental health outcome measure. *Journal of Personality Assessment, 73*, 359–373.

Bryan, C. J., Bryan, A. O., Anestis, M. D., Anestis, J. C., Green, B. A., Etienne, N., ... & Ray-Sannerud, B. (2016). Measuring moral injury: Psychometric properties of the moral injury events scale in two military samples. *Assessment*, *23*(5), 557-57.

Charman, S., & Bennett, S. (2021). Voluntary resignations from the police service: the impact of organisational and occupational stressors on organisational commitment. *Policing and Society,* 1-2.

Cloitre, M., Shevlin M., Brewin, C.R., Bisson, J.I., Roberts, N.P., Maercker, A., Karatzias, T., Hyland, P. (2018). The International Trauma Questionnaire: Development of a self-report measure of ICD-11 PTSD and Complex PTSD. *Acta Psychiatrica Scandinavica.* DOI: 1.1111/acps.12956

Currier, J. M., Kuhlman, S., & Smith, P. N. (2015). Empirical and ethical considerations for addressing spirituality among veterans and other military populations at risk for suicide. *Spirituality in Clinical Practice*, *2*(1), 68.

Currier, J. M., Smith, P. N., & Kuhlman, S. (2017). Assessing the unique role of religious coping in suicidal behavior among U.S. Iraq and Afghanistan veterans. *Psychology of Religion and Spirituality, 9*(1), 118–123. [https://doi.org/1.1037/rel0000055](https://psycnet.apa.org/doi/10.1037/rel0000055)

Cyr, G., Belanger, C., & Godbout, N. (2022). French translation and validation of the International Trauma Questionnaire in a Canadian community sample. *Child Abuse & Neglect*, *128*, 105627.

Frankfurt, S., & Frazier, P. (2016) A review of research on moral injury in combat veterans, *Military Psychology,* 28(5), 318-330, DOI:1.1037/mil0000132

Fani, N., Currier, J. M., Turner, M. D., Guelfo, A., Kloess, M., Jain, J., ... & Turner, J. A. (2021). Moral injury in civilians: associations with trauma exposure, PTSD, and suicide behavior. *European Journal of Psychotraumatology*, *12*(1), 1965464.

Garnefski, N., & Kraaij, V. (2006). Relationships between cognitive emotion regulation strategies and depressive symptoms: A comparative study of five specific samples. *Personality and Individual differences*, *40*(8), 1659-1669.

Haller, H., H. Cramer, R. Lauche, F. Gass, and G. J. Dobos. (2014). The prevalence and burden of subthreshold generalized anxiety disorder: a systematic review. *BMC Psychiatry* 14:128.

Kraaij, V., & Garnefski, N. (2019). The behavioral emotion regulation questionnaire: development, psychometric properties and relationships with emotional problems and the cognitive emotion regulation questionnaire. *Personality and Individual Differences*, *137*, 56-61.

Kroenke, K., Spitzer, R. L., & Williams, J. B. (2001). The PHQ-9: Validity of a brief depression severity measure. *Journal of General Internal Medicine, 16*, 606-613.

Litvin, J. M., Kaminski, P. L., & Riggs, S. A. (2017). The complex trauma inventory: A self-report measure of posttraumatic stress disorder and complex posttraumatic stress disorder. *Journal of Traumatic Stress, 30*(6), 602-613. doi: 1.1002/jts.22231

Litz, B., Stein, N., Delaney, E., Lebowitz, L., Nash, W. P., Silva, C., & Maguen, S. (2009). Moral injury and moral repair in war veterans: A preliminary model and intervention strategy. *Clinical Psychology Review, 29*, 695-706.

Murphy, D., Shevlin, M., Pearson, E., Greenberg, N., Wessely, S., Busuttil, W., & Karatzias, T. (2020). A validation study of the International Trauma Questionnaire to assess post-traumatic stress disorder in treatment-seeking veterans. *The British Journal of Psychiatry*, *216*(3), 132-137.

Nash, W. P., Marino-Carper, T. L., Mills, M. A., Au, T., Goldsmith, A., & Litz, B. T. (2013). Psychometric evaluation of the Moral Injury Events Scale. *Military Medicine, 178*, 646-652.

Ogles, B. M., Lambert, M. J., & Fields, S. A. (2002). *Essentials of outcome assessment*. John Wiley & Sons Inc.

Pargament, K., Feuille, M., & Burdzy, D. (2011). The Brief RCOPE: Current psychometric status of a short measure of religious coping. *Religions*, *2*(1), 51-76.

Pargament, K. I., Smith, B. W., Koenig, H. G., & Perez, L. (1998). Patterns of positive and negative religious coping with major life stressors. *Journal for the Scientific Study of Religion, 37*(4), 710-724.

Remes, O., Brayne, C., Van Der Linde, R., & Lafortune, L. (2016). A systematic review of reviews on the prevalence of anxiety disorders in adult populations. *Brain and behavior*, *6*(7), e00497.

Remes,O., Wainwright N, Surtees P, et al. (2018). Generalised anxiety disorder and hospital admissions: findings from a large, population cohort study. *British Medical Journal Open*, 8:e018539. doi:1.1136/ bmjopen-2017-018539

Richards, K., Campenni, C., & Muse-Burke, J. (2010). Self-care and well-being in mental health professionals: The mediating effects of self-awareness and mindfulness. *Journal of Mental Health Counseling*, *32*(3), 247-264.

Slee, A., Nazareth, I., Freemantle, N., & Horsfall, L. (2021). Trends in generalised anxiety disorders and symptoms in primary care: UK population-based cohort study. *The British Journal of Psychiatry*, *218*(3), 158-164.

Spitzer, R. L., Kroenke, K., Williams, J. B. W., & Lowe, B. (2006). A brief measure for assessing generalized anxiety disorder. *Archives of Internal Medicine, 166*, 1092-1097.

Smith, D. J., Nicholl, B. I., Cullen, B., Martin, D., Ul-Haq, Z., Evans, J., ... & Pell, J. P. (2013). Prevalence and characteristics of probable major depression and bipolar disorder within UK biobank: cross-sectional study of 172,751 participants. *PloS one*, *8*(11), e75362.

Tabachnick, B. G., & Fidell, L. S. (2001). *Using multivariate statistics.* Boston, MA: Allyn & Bacon.

Tully, P. J., and S. M. Cosh. 2013. Generalized anxiety disorder prevalence and comorbidity with depression in coronary heart disease: a meta-analysis. *Journal of Health Psychology, 18*, 1601–1616.

Young, J. L., Waehler, C. A., Laux, J. M., McDaniel, P. S., & Hilsenroth, M. J. (2003). Four studies extending the utility of the Schwartz Outcome Scale (SOS-10). *Journal of personality assessment*, *80*(2), 130-138.

1. Originally, eight participants selected *Its complicated* and clarified things like ‘mine are over 18’ (recoded as *no children under 18*) or *pregnant* (coded as *yes children under 18*). Two unclear cases were coded as missing variables. [↑](#footnote-ref-1)
2. Originally, four participants selected *Its complicated;* three clarified things like *caregiving is intermittent* (recoded as *yes caregiver*); one unclear case was recoded as a missing variable. [↑](#footnote-ref-2)
3. That said, we asked about symptoms over the past month whereas Smith et al. (2013) asked about symptoms in the past two weeks, so the comparison is not perfect. [↑](#footnote-ref-3)
4. We converted scores from the typical presentation of results as sum from 0-60 to a mean from 1-7. [↑](#footnote-ref-4)
