# Supplementary material for: Protecting the Protectors: Moral Injury, Coping Styles, and Mental Health of UK Police Officers and Staff Investigating Child Sexual Abuse Material
Source: Depress Anxiety. 2024 Nov 23;2024:1854312. doi: 10.1155/da/1854312 (PMC11922302; doi:10.1155/da/1854312)
Supplement: Supporting Information 2 — File S2: Online Sample provides the methods, results, and brief discussion of the UK online civilian comparison sample, including tables presenting correlations and regressions. [file 1854312.f2.docx]

**Online Supplemental Materials: Online Sample UK Civilians via Prolific**

**Protecting the Protectors: Moral Injury, Coping Styles, and Mental Health of UK Police Officers and Staff Investigating Child Sexual Abuse Material**

**Participants**

We recruited 422 UK adult participants via Prolific, who received £2 each. We removed one person who refused reconsent, three who completed less than 50% of the study, and six who completed the study in less than 200 seconds, leaving a sample of 408. We excluded 8 people who reported they were police officers or police staff. Finally, we selected all 385 remaining participants who passed the attention check. According to G*Power, a sample of 385 participants provides 95% power to detect Cohen’s *f*^2^ = .07 at α = .025 in a linear multiple regression with 9 predictors and 10 control variables (i.e., the most complex analysis in this study). In addition, we had 95% power to detect an effect of Cohen’s *d* = .25 at (two-tailed) α = .025 for comparisons across police and control samples.

The online sample had a similar demographic profile to the police sample: *M*_age_ = 39.91, *SD* = 13.89, 118 male (31.2%), 260 female (67.5%), six nonbinary and one unreported. Regarding marital status, 207 (53.8%) were partnered and 178 (46.2%) unpartnered. The majority (*n* = 357, 92.47%) identified as *White, British,* or *Caucasian*, with 8 reporting *Mixed*, 5 reporting *Black or Caribbean*, 11 as *Asian or Indian*, and 2 as *Arabic* or *Middle Eastern*, 1 as *Latina*, and 6 did not report ancestry.

Regarding parental status, 121 (34.1%) participants reported they were a parent or guardian for children under 18, 263 (68.3%) reported they were not, and 1 (05%) reported *it’s complicated* (recoded as living with children). Regarding caregiving for someone over 18, 353 (91.7%) reported they were not a caregiver, 27 (7.0%) reported they were a caregiver, and five (1.3%) reported *it’s complicated* (recoded as caregiving). Regarding therapy, 241 participants (62.6%) reported never receiving therapy, whereas 144 (37.4%) reported receiving therapy at some point either currently or in the past.

On average, few participants reported strong spiritual or religious beliefs (*M* = 2.17, *SD* = 1.55). Regarding faith, 176 (46.0%) identified as atheist or no religion, 105 (27.2%) as Christian, 47 (12.2%) as spiritual, 27 (7.0%) as agnostic, 6 (1.6%) as Muslim, 6 (1.6%) as Pagan, Nature, or Wiccan, 4 (1.0%) as Catholic, 4 (1.0%) as Buddhist, 3 (0.8%) as Church of England, 2 (0.5%) as Jewish, 1 (0.3%) as Hindu, 1 (0.3%) as Satanist, and 3 (0.8%) did not report a religious affiliation.

Overall, the police and prolific samples looked fairly similar in terms of age, gender, ethnicity, caregiving, strength of faith and the faiths people endorsed, and therapy experience. There was a moderately higher proportion of males and partnered people in the police than online sample, slightly more ethic and faith diversity in the online than police sample. The samples differed notably in only one respect: there were substantially higher rates of parenting children in the police versus online samples (56% vs 34%). However, parenting did not significantly predict any outcomes variables (see below).

**Procedure and Materials**

Participants completed all measures online via Qualtrics. Police and online civilians completed the same measures, except we did not ask the online sample about contact, exposure, and support factors, resource availability, use, and helpfulness, or barriers to seeking support, as all of these questions presume employment in CSAE settings. Online sample participants completed the same demographic questions as the police sample except those regarding rank, role, and work region.

***The Moral Injury Events Scale (MIES)***

For the online sample, we modified the prompt to be more generic: *Sometimes in life people deal with morally complex or ambiguous situations. When thinking about your involvement with morally complex or ambiguous situations, do you agree with the following statements?* Participants then completed the same items as the CSAE version. We then conducted the same principal components analysis, which this time revealed only two factors with eigenvalues greater than 1 (5.06, 1.49, with a third factor scoring .72). The two items >1 explained 72.79% of the variance.

In terms of loading, all Transgressions-Self items loaded on one factor, and all Transgressions-Other and Betrayal factors loaded on the second factor. This pattern suggests that civilian participants may draw less of a distinction than police or military personnel between moral injury from colleagues versus superiors and institutions, or this may reflect the more general wording of the prompt. Nonetheless, when allowing for three factors, each item loaded on the same factors as the CSAE version, and alphas were acceptable: Transgressions-Other, α = .82, Transgressions-Self, α = .94, Betrayal, α = .82, full scale, α = .90. Therefore, for maximum comparability with the police sample, we retained the same three-factor breakdown for analysis.

***Short Cognitive and Emotion Regulation Questionnaire (CERQ) and Behavioral Emotion Regulation Questionnaire (BERQ)***

For these questionnaires we again modified the prompt to read *When thinking about your involvement with morally complex or ambiguous situations…* They showed high reliability in this as well as the police samples: self-blame (α = .81), other-blame (α = .78), rumination (α = .71), catastrophizing (α = .83), positive refocusing (α = .83), planning (α = .68), positive reappraisal (α = .82), putting into perspective (α = .71), and acceptance (α = .82). Reliability was also similar for the BERQ in the online sample: *seeking distraction* (α = .81), *withdrawal* (α = .87), *actively approaching* (α = .86), *seeking social support* (α = .85), and *ignoring* (α = .75).

***Brief Religious Coping Scale (RCOPE)***

The online sample showed a similar patter as the police sample in the factor analysis, in the online sample, two factors with eigenvalues greater than 1 (8.83, 1.70), accounting for 75.25% of variance. Again, positive items loaded on *positive coping* (α = .96), and all negative items loading on *negative coping* (online α = .90), except *decided the devil made this happen.*

***Patient Health Questionnaire-9 (PHQ-9) and Generalized Anxiety Disorder-7 (GAD-7)***

We computed the mean across all PHQ-9 items (α = .93) and GAD-7 items (α = .94). We also computed the number of participants meeting the clinical criteria for diagnosis for each measure.

***PTSD: The International Trauma Questionnaire (ITQ)***

The ITQ showed similar reliability in this sample (i.e., Complex PTSD α = .97), PTSD cluster (α = .95,) and Disturbance in Self-organization cluster (α = .95). We also computed the number of participants meeting the clinical criteria for a PTSD and C-PTSD diagnosis.

***Wellbeing: Schwartz Outcome Scale (SOS-10)***

We computed the mean (α = .95).

**Results and Discussion**

**Clinical Prevalence**

First, we computed the clinical prevalence of depression, anxiety, and PTSD according to diagnostic criteria.

***Depression***

We found 183 of 661, or 27.7% of police participants and 142 of 422, or 36.9% of the online sample reported symptoms within the past month that may qualify for major depression according to this measure (see Table S1). Mean scores on the PHQ-9 showed lower average anxiety rates in the police sample, *M* = 0.79, *SD* = 0.61, than online civilians, *M* = 0.94, *SD* = 0.76, *t*(672.02) = -3.29, *p* < .001, *d* = -.22 (note that Levene’s test was significant for this and all mean comparisons, so we always report adjusted degrees of freedom to account for heterogeneity in variance). This result was inconsistent with our hypothesis that the police sample would score higher than the online sample. Yet, considering the generally lower rates in other UK samples, the difference may in fact reflect elevated depression rates among people frequently performing online work tasks for relatively low pay (i.e., Prolific workers) rather than lower rates among police personnel, who still seem to show substantially elevated rates compared to the general population. For comparison, Engel and colleagues (2020) found a depression prevalence of 26% among American Mturkers.

**Table S1**

*UK Police and Online Civilian Participants Scoring in Each Clinical Range on the Patient Health Questionnaire-9*

| Severity | Police sample | | Online Sample | |
| --- | --- | --- | --- | --- |
|  | *N* | Percent | *N* | Percent |
| Minimal | 245 | 37.1 | 128 | 33.2 |
| Mild | 233 | 35.2 | 115 | 29.9 |
| Moderate | 108 | 16.3 | 67 | 17.4 |
| Moderately Severe | 50 | 7.6 | 40 | 10.4 |
| Severe | 25 | 3.8 | 35 | 9.1 |

***Generalized Anxiety***

We found that 160 of 661, or 24.2% of police participants, and 126 of 385, or 32.8% of online participants, reported symptoms within the past month that may qualify for generalized anxiety according to this measure (see Table S2). For mean scores on the GAD7, there was no significant difference in average anxiety rates in police personnel, *M* = 0.92, *SD* = 0.72, versus online civilians, *M* = 1.02, *SD* = 0.84, *t*(715.15) = -1.84, *p* = .06, *d* = -.12. As with depression, contrary to hypotheses, anxiety rates were lower in the police personnel sample, than online civilian sample. Yet, comparisons to broader samples suggests this finding may not reflect low rates among police personnel, but rather high rates among both police and online participants. For comparison, Engel and colleagues (2020) found anxiety prevalence of 22% among American Mturkers.

**Table S2**

*UK Police and Online Civilian Participants Scoring in Each Clinical Range on the Generalized Anxiety Disorder-7*

| Severity | Police Sample | | Online Sample | |
| --- | --- | --- | --- | --- |
|  | *N* | Percent | *N* | Percent |
| Minimal | 279 | 42.2 | 164 | 42.6 |
| Mild | 222 | 33.6 | 95 | 24.7 |
| Moderate | 105 | 15.9 | 73 | 19.0 |
| Severe | 55 | 8.3 | 53 | 13.8 |

***PTSD and Complex PTSD***

In the Police sample, we found that 22 participants (3.3%) met criteria for clinical levels of PTSD, and 36 (5.4%) met criteria for complex-PTSD, for a total of 8.7%. In the online sample, we found that 11 participants (2.9%) met criteria for clinical levels of PTSD, and 45 (11.7%) met criteria for complex-PTSD, for a total of 14.6% (see Table S3). Again, inconsistent with hypotheses, the police sample scored lower in PTSD than the online sample. This pattern held across PTSD (police, *M* = 0.71, *SD* = 0.76, online *M* = 0.97, *SD* = 1.01), *t*(636.47) = -4.52, *p* < .001, *d* = -.29, Disturbance in the Self, (police, *M* = 0.85, *SD* = 0.88, online *M* = 1.25, *SD* = 1.10), *t*(666.81) = -6.25, *p* < .001, *d* = -.43, and overall Complex PTSD, (police, *M* = 0.78, *SD* = 0.76, online *M* = 1.11, *SD* = 1.01), *t*(637.89) = -5.53, *p* < .001, *d* = -.38. Again, this result suggests somewhat poor mental health among the online sample. Unlike the results for anxiety and depression, however, the police sample does not appear to have especially elevated rates of PTSD compared to other sources. For comparison, Engel and colleagues (2020) found a PTSD prevalence of 11% among American Mturkers.

**Table S3**

*UK Police and Online Civilian Participants Scoring in Clinical Range on PTSD and Complex PTSD*

| Diagnosis | Police sample | | Online Sample | |
| --- | --- | --- | --- | --- |
|  | *N* | Percent | *N* | Percent |
| Not Clinical | 603 | 91.2 | 329 | 85.4 |
| PTSD | 22 | 3.3 | 11 | 2.9 |
| Complex PTSD | 36 | 5.4 | 45 | 11.7 |

***Wellbeing***

Participants in the police sample, *M* = 4.70, *SD* = 1.11, scored significantly higher than the online sample, *M* = 4.29, *SD* = 1.26, *t*(720.35) = 5.25, *p* < .001, *d* = .35. Again, this finding was inconsistent with hypotheses, but consistent with the results for depression, anxiety, and PTSD, suggesting particularly poor mental health among the online sample.

**Correlational & Regression Analyses**

Next, we computed bivariate correlations between all measures in the study (see Table S4). Then we conducted the same set of linear multiple regressions as the police sample, controlling for the same demographic factors, except those not measured in this sample: officer versus staff, career length, role length. We anticipated largely replicating the same predictive patterns as the main sample.

***Demographic Predictors***

We entered demographic predictors at step 1 of each analysis. Similar to the police sample, most failed to predict significant variance in any outcomes, with three primary exceptions (see Tables S5-S8). As in the police sample, people who reported ever attending therapy tended to report higher rates of moral injury, depression, anxiety, and PTSD, and reduced wellbeing. Second, religiosity: also similar to the police sample, people who reported greater religiosity also reported higher rates of moral injury. However, unlike the police sample, there was no significant relationship between religiosity and anxiety, depression, PTSD, or wellbeing. Finally, unlike the police sample, age was a significant predictor: younger people reported higher rates of anxiety, depression, and PTSD than older people.

***Moral Injury***

Results largely corroborated the police sample findings: betrayal was a significant predictor of all outcomes, and no other finding was significant (see Table S5). Unlike the police sample, transgression-self did not predict outcomes.

***Cognitive and Emotion Regulation***

Again, results largely corroborated the police sample findings (see Table S6). Self-blame, other-blame, and catastrophizing predicted worse outcomes, whereas planning and positive reappraisal predicted better outcomes. Unlike the police sample, rumination, putting into perspective, and positive refocusing did not significantly predict outcomes.

***Behavioral Emotion Regulation Questionnaire (BERQ)***

Consistent with the police sample, withdrawal predicted worse outcomes whereas approach predicted better outcomes (see Table S7). Unlike the police sample seeking distraction, social support, and ignoring did not significantly impact outcomes.

***Religious Coping***

Consistent with the police sample, negative religious coping predicted worse outcomes (see Table S8). Unlike the police sample, we also found some evidence for positive religious coping predicting better wellbeing.

**Table S4a**

*Correlations Between All Measures in the Study*

| Variable | 1 | 2 | 3 | 4 | 5 | 6 | 7 | 8 | 9 | 10 | 11 | 12 | 13 | 14 | 15 | 16 | 17 | 18 | 19 | 20 |
| --- | --- | --- | --- | --- | --- | --- | --- | --- | --- | --- | --- | --- | --- | --- | --- | --- | --- | --- | --- | --- |
| 1. Depression | - |  |  |  |  |  |  |  |  |  |  |  |  |  |  |  |  |  |  |  |
| 2. Anxiety | **.83^**^** | - |  |  |  |  |  |  |  |  |  |  |  |  |  |  |  |  |  |  |
| 3. C-PTSD | **.88^**^** | **.82^**^** |  |  |  |  |  |  |  |  |  |  |  |  |  |  |  |  |  |  |
| 4. PTSD | **.81^**^** | **.80^**^** | **.95^**^** | - |  |  |  |  |  |  |  |  |  |  |  |  |  |  |  |  |
| 5. Disturbance of Self | **.87^**^** | **.78^**^** | **.96^**^** | **.84^**^** | - |  |  |  |  |  |  |  |  |  |  |  |  |  |  |  |
| 6. Wellbeing | **-.73^**^** | **-.69^**^** | **-.73^**^** | **-.63^**^** | **-.77^**^** | - |  |  |  |  |  |  |  |  |  |  |  |  |  |  |
| 7. Transgress Self | **.37^**^** | **.31^**^** | **.39^**^** | **.40^**^** | **.34^**^** | **-.29^**^** | - |  |  |  |  |  |  |  |  |  |  |  |  |  |
| 8. Transgress Other | **.33^**^** | **.28^**^** | **.36^**^** | **.32^**^** | **.37^**^** | **-.28^**^** | **.52^**^** | - |  |  |  |  |  |  |  |  |  |  |  |  |
| 9. Betrayal | **.40^**^** | **.36^**^** | **.44^**^** | **.46^**^** | **.40^**^** | **-.34^**^** | **.62^**^** | **.48^**^** | - |  |  |  |  |  |  |  |  |  |  |  |
| 10. Self-Blame | **.28^**^** | **.30^**^** | **.37^**^** | **.35^**^** | **.37^**^** | **-.29^**^** | **.24^**^** | **.51^**^** | **.26^**^** | - |  |  |  |  |  |  |  |  |  |  |
| 11. Acceptance | -.02 | -.03 | -.02 | .00 | -.04 | **.21^**^** | **.16^**^** | **.16^**^** | .10 | **.15^**^** | - |  |  |  |  |  |  |  |  |  |
| 12. Rumination | **.40^**^** | **.47^**^** | **.47^**^** | **.46^**^** | **.45^**^** | **-.38^**^** | **.34^**^** | **.32^**^** | **.36^**^** | **.44^**^** | .04 | - |  |  |  |  |  |  |  |  |
| 13. Positive Refocusing | **-.19^**^** | **-.18^**^** | **-.19^**^** | **-.13^**^** | **-.22^**^** | **.33^**^** | .03 | -.04 | .03 | -.05 | **.31^**^** | **-.19^*^** | - |  |  |  |  |  |  |  |
| 14. Planning | -.03 | .07 | .06 | **.14^*^** | -.01 | **.18^**^** | **.16^**^** | .07 | .10 | **.22^**^** | **.32^**^** | **.26^**^** | **.21^**^** | - |  |  |  |  |  |  |
| 15. Positive Reappraisal | **.24^**^** | **-.27^**^** | **-.27^**^** | **-.19^**^** | **-.33^**^** | **-.47^**^** | -.03 | -.06 | -.06 | -.03 | **.34^**^** | **-.17^**^** | **.46^**^** | **.42^**^** | - |  |  |  |  |  |
| 16. Put into Perspective | **-.13^*^** | **-.16^**^** | **-.14^**^** | -.09 | **-.20^**^** | **.31^**^** | .07 | -.04 | .03 | -.03 | **.45^**^** | -.10 | **.49^**^** | **.30^**^** | **.63^**^** | - |  |  |  |  |
| 17. Catastrophizing | -.03 | **.52^*^** | **.56^**^** | **.53^**^** | **.55^**^** | **-.49^**^** | **.43^**^** | **.42^**^** | **.44^**^** | **.46^**^** | .00 | **.77^**^** | **-.17^**^** | **.17^**^** | **-.25^**^** | **-.16^**^** | - |  |  |  |
| 18. Other-Blame | **-.18^**^** | **.13^*^** | **.23^*^** | **.26^**^** | **.17^**^** | -.11 | **.30^**^** | .07 | **.37^**^** | -.05 | **.13^**^** | **.22^**^** | **.12^*^** | .11 | .00 | .11 | **.29^**^** | - |  |  |
| 19. Seeking Distraction | -.06 | -.07 | -.04 | .01 | **-**.10 | **.22^**^** | .08 | .10 | .02 | .05 | **.26^**^** | -.02 | **.52^**^** | **.20^**^** | **.30^**^** | **.36^**^** | -.01 | **.15^**^** | - |  |
| 20. Withdrawal | **.59^**^** | **.57^**^** | **.64^**^** | **.56^**^** | **.66^**^** | **-.57^**^** | **.31^**^** | **.30^**^** | **.36^**^** | **.38^**^** | -.01 | **.45^**^** | **-.19^**^** | .04 | **-.29^**^** | **-.19^**^** | **.52^**^** | **.17^**^** | -.04 | - |
| 21. Approach | **-.31^**^** | **-.31^**^** | **-.26^**^** | **-.18^**^** | **-.33^**^** | **.49^**^** | .00 | -.05 | -.06 | -.03 | **.28^**^** | -.11 | **.27^**^** | **.52^**^** | **.48^**^** | **.32^**^** | **-.18^**^** | .03 | **.27^**^** | **-.33^**^** |
| 22. Social Support | **-.20^**^** | -.08 | **-.14^**^** | -.05 | **.22^**^** | **.31^**^** | .02 | -.08 | .00 | -.07 | .07 | .10 | **.23^**^** | **.28^**^** | **.21^**^** | .10 | -.01 | .03 | **.13^**^** | **-.20^**^** |
| 23. Ignoring | **.30^**^** | **.21^**^** | **.29^**^** | **.28^**^** | **.28^**^** | **-.19^**^** | **.04^*^** | **.24^**^** | **.14^**^** | **.24^**^** | .08 | .09 | .10 | -.09 | -.04 | .08 | **.16^**^** | **.15^**^** | **.32^**^** | **.36^**^** |
| 24. Pos Religious Cope | .09 | .11 | .01 | **.12^*^** | .07 | -.06 | **.17^**^** | **.21^*^** | **.14^**^** | **.12^*^** | **.19^*^** | .01 | **.12^*^** | **.14^*^** | .10 | .08 | .11 | .09 | .00 | .00 |
| 25. Neg Religious Cope | **-.27^**^** | **.30^**^** | **.31^**^** | **.32^**^** | **.28^**^** | **-.25^**^** | **.23^**^** | **.16^*^** | **.24^**^** | **.16^**^** | .10 | **.23^*^** | .06 | .10 | -.02 | .02 | **.25^**^** | **.18^**^** | -.01 | .21**^**^** |
| 26. Age | **-.26^**^** | **-.25^**^** | **.24^**^** | **-.22^**^** | **-.24^**^** | **.14^**^** | **-.18^**^** | **.20^**^** | -.06 | **-.20^**^** | -.07 | **-.20^*^** | -.01 | -.10 | .01 | -.01 | **-.20^**^** | .01 | **-.15^**^** | **-.23^**^** |
| 27. Gender (1=*m*, 2=*f*) | .07 | **.15^**^** | .08 | .09 | .05 | -.01 | .06 | .00 | .11 | -.04 | -.02 | .08 | .05 | -.10 | **.13^*^** | -.04 | **.15^**^** | .07 | -.01 | .07 |
| 28. Partnered (1=*y*, 0=*n*) | **.13^**^** | -.06 | -.11 | -.05 | **-.15^**^** | **.12^*^** | -.11 | -.09 | -.01 | **-.12^*^** | -.04 | -.05 | .03 | -.05 | .01 | .02 | -.06 | -.02 | -.07 | -.11 |
| 29. Parent (1=*y*, 0=*n*) | -.05 | .03 | -.02 | -.01 | -.01 | -.02 | -.01 | .04 | .05 | .01 | .01 | .05 | .02 | .08 | .05 | .06 | .07 | -.04 | .00 | .00 |
| 30. Caregiver (1=*y*, 0=*n*) | .06 | .09 | .06 | .06 | .06 | .10 | .03 | .01 | -.03 | .02 | .01 | .02 | -.03 | -.01 | -.04 | -.04 | .03 | -.05 | .03 | .09 |
| 31. Therapy (1=*y*, 0=*n*) | **.33^**^** | **.34^**^** | **.39^**^** | **.37^**^** | **.39^**^** | **-.33^**^** | **.23^**^** | **.21^**^** | **.23^**^** | **.21^**^** | .02 | **.26^**^** | -.07 | .10 | **-.16^**^** | **-.16^**^** | **.35^**^** | .07 | .10 | **.36^**^** |
| 32. Religiosity | .05 | .07 | .04 | .05 | .02 | -.03 | .11 | **.14^*^** | .08 | .07 | .09 | .05 | .04 | .04 | .04 | .04 | .08 | -.02 | -.03 | .03 |

*Note:* bold indicates significance. * *p* < .025, ** *p* < .01

**Table S4b**

*Correlations Between All Measures in the Study (Continued)*

| Variable | 21 | 22 | 23 | 24 | 25 | 26 | 27 | 28 | 29 | 30 | 31 |
| --- | --- | --- | --- | --- | --- | --- | --- | --- | --- | --- | --- |
| 21. Approach | - |  |  |  |  |  |  |  |  |  |  |
| 22. Social Support | **.46^**^** | - |  |  |  |  |  |  |  |  |  |
| 23. Ignoring | **-.25^**^** | **.14^**^** | - |  |  |  |  |  |  |  |  |
| 24. Pos Religious Cope | .07 | **-.22^**^** | .02 | - |  |  |  |  |  |  |  |
| 25. Neg Religious Cope | -.09 | -.03 | **.14^**^** | **.66^**^** | - |  |  |  |  |  |  |
| 26. Age | .05 | -.07 | **-.22^**^** | .04 | .01 | - |  |  |  |  |  |
| 27. Gender (1=*m*, 2=*f*) | -.07 | **.15^*^** | -.03 | .02 | .02 | -.06 | - |  |  |  |  |
| 28. Partnered (1=*y*, 0=*n*) | -.02 | .10 | -.07 | .06 | .04 | **.32^**^** | .00 | - |  |  |  |
| 29. Parent (1=*y*, 0=*n*) | .08 | .05 | -.04 | .09 | .05 | -.03 | .07 | **.32^**^** | - |  |  |
| 30. Caregiver (1=*y*, 0=*n*) | .05 | -.05 | -.03 | .04 | .06 | .11 | -.04 | .01 | .02 | - |  |
| 31. Therapy (1=*y*, 0=*n*) | -.07 | .06 | .01 | .03 | **.12^*^** | **.19^**^** | **.15^**^** | **-.13^**^** | -.03 | .06 | - |
| 32. Religiosity | .00 | .04 | -.01 | **.68^**^** | **.37^**^** | .03 | .08 | .03 | .10 | .00 | .04 |

*Note:* bold indicates significance. * *p* < .025, ** *p* < .01

**Table S5**

*Moral Injury Subscales: Transgression-Self, Transgression-Other, and Betrayal, Predict Depression, Generalized Anxiety, Complex PTSD, and Wellbeing Beyond Demographics*

| **Predictors** |  | **Depression** | | |  | **Generalized Anxiety** | | |  | **Complex PTSD** | | |  | **Wellbeing** | | |
| --- | --- | --- | --- | --- | --- | --- | --- | --- | --- | --- | --- | --- | --- | --- | --- | --- |
|  |  | **β** | ***t*** | ***p*** |  | **β** | ***t*** | ***p*** |  | **β** | ***t*** | ***p*** |  | **β** | ***t*** | ***p*** |
| **Step 1** |  |  |  |  |  |  |  |  |  |  |  |  |  |  |  |  |
| Age |  | **-0.20** | **-3.74** | **<.001** |  | **-0.20** | **-3.81** | **<.001** |  | **-0.16** | **-3.16** | **.002** |  | 0.06 | 1.06 | .288 |
| Gender |  | 0.01 | 0.31 | .759 |  | 0.09 | 1.85 | .065 |  | 0.01 | 0.13 | .895 |  | 0.04 | 0.85 | .396 |
| Relationship Status  (1 = *partnered*,  0 = *not partnered*) |  | -0.04 | -0.68 | .500 |  | 0.03 | 0.50 | .621 |  | -0.02 | -0.27 | .788 |  | 0.09 | 1.65 | .100 |
| Parent of <18  (1=*yes*, 0=*no*) |  | -0.04 | -0.83 | .409 |  | 0.01 | 0.14 | .889 |  | -0.02 | -0.34 | .735 |  | -0.05 | -0.10 | .320 |
| Caregiver >18  (1=*yes*, 0=*no*) |  | 0.07 | 1.37 | .171 |  | 0.09 | 1.95 | .052 |  | 0.06 | 1.31 | .190 |  | -0.09 | -1.79 | .074 |
| Therapy Experience  (1=*yes*, 0=*no*) |  | **0.27** | **5.51** | **<.001** |  | **0.28** | **5.77** | **<.001** |  | **0.35** | **7.18** | **<.001** |  | **-0.30** | **-5.87** | **<.001** |
| Religiosity |  | 0.04 | 0.86 | .388 |  | 0.06 | 1.26 | .207 |  | 0.04 | 0.75 | .455 |  | -0.03 | -0.68 | .500 |
| **Step 2** |  |  |  |  |  |  |  |  |  |  |  |  |  |  |  |  |
| Transgression-Self |  | 0.09 | 1.62 | .107 |  | 0.07 | 1.20 | .231 |  | 0.12 | 2.17 | .031 |  | -0.09 | -1.61 | .109 |
| Transgression-Other |  | 0.09 | 1.54 | .125 |  | 0.05 | 0.73 | .466 |  | 0.07 | 1.10 | .272 |  | -0.03 | -0.50 | .620 |
| Betrayal |  | **0.24** | **3.98** | **<.001** |  | **0.23** | **3.72** | **<.001** |  | **0.28** | **4.74** | **<.001** |  | **-0.22** | **-3.47** | **.001** |

*Note:* bold indicates significance.

**Table S6**

*Cognitive and Emotional Regulation Subscales: Self-blame, Acceptance, Rumination, Positive Refocusing, Planning, Positive Reappraisal, Putting into Perspective, Catastrophizing, and Other-blame Predict Moral Injury, Depression, Generalized Anxiety, Complex PTSD, and Wellbeing Beyond Demographics*

| **Predictors** |  | **Moral Injury** | | |  | **Depression** | | |  | **Generalized Anxiety** | | |  | **Complex PTSD** | | |  | **Wellbeing** | | |
| --- | --- | --- | --- | --- | --- | --- | --- | --- | --- | --- | --- | --- | --- | --- | --- | --- | --- | --- | --- | --- |
|  |  | **β** | ***t*** | ***p*** |  | **β** | ***t*** | ***p*** |  | **β** | ***t*** | ***p*** |  | **β** | ***t*** | ***p*** |  | **β** | ***t*** | ***p*** |
| **Step 1** |  |  |  |  |  |  |  |  |  |  |  |  |  |  |  |  |  |  |  |  |
| Age |  | -0.12 | -2.19 | .029 |  | **-0.20** | **-3.74** | **<.001** |  | **-0.20** | **-3.81** | **<.001** |  | **-0.16** | **-3.16** | **.002** |  | 0.06 | 1.06 | .288 |
| Gender |  | 0.01 | 0.08 | .935 |  | 0.02 | 0.301 | .759 |  | 0.09 | 1.85 | .065 |  | 0.01 | 0.13 | .895 |  | 0.04 | 0.85 | .396 |
| Relationship Status  (1 = *partnered*,  0 = *not partnered*) |  | -0.04 | -0.67 | .504 |  | -0.04 | -0.68 | .500 |  | 0.03 | 0.50 | .621 |  | -0.02 | -0.27 | .788 |  | 0.09 | 1.65 | .100 |
| Parent of <18  (1=*yes*, 0=*no*) |  | 0.05 | 0.90 | .367 |  | -0.04 | -0.83 | .409 |  | 0.01 | 0.14 | .889 |  | -0.02 | -0.39 | .735 |  | -0.05 | -1.00 | .320 |
| Caregiver >18  (1=*yes*, 0=*no*) |  | 0.01 | 0.08 | .938 |  | 0.07 | 1.37 | .171 |  | 0.09 | 1.95 | .052 |  | 0.06 | 1.31 | .190 |  | -0.09 | -1.79 | .074 |
| Therapy Experience  (1=*yes*, 0=*no*) |  | **0.23** | **4.49** | **<.001** |  | **0.27** | **5.51** | **<.001** |  | **0.28** | **5.77** | **<.001** |  | **0.35** | **7.18** | **<.001** |  | **-0.30** | **-5.87** | **<.001** |
| Religiosity |  | **0.12** | **2.40** | **.017** |  | 0.04 | 0.86 | .388 |  | 0.06 | 1.26 | .207 |  | 0.04 | 0.75 | .455 |  | -0.03 | -0.68 | .500 |
| **Step 2** |  |  |  |  |  |  |  |  |  |  |  |  |  |  |  |  |  |  |  |  |
| Self-Blame |  | **0.28** | **5.43** | **<.001** |  | 0.08 | 1.61 | .109 |  | 0.08 | 1.52 | .130 |  | **0.18** | **3.59** | **<.001** |  | **-0.14** | **-3.03** | **.003** |
| Acceptance |  | 0.10 | 2.07 | .040 |  | -0.01 | -0.07 | .945 |  | -0.03 | -0.55 | .584 |  | -0.03 | -0.61 | .544 |  | **0.12** | **2.52** | **.012** |
| Rumination |  | -0.01 | -0.17 | .864 |  | 0.07 | 0.96 | .339 |  | **0.16** | **2.30** | **.022** |  | 0.06 | 0.88 | .379 |  | -0.05 | -0.83 | .409 |
| Positive Refocusing |  | 0.02 | 0.34 | .738 |  | -0.11 | -2.17 | .031 |  | -0.08 | -1.47 | .143 |  | -0.08 | -1.75 | .081 |  | 0.10 | 2.14 | .033 |
| Planning |  | -0.08 | -1.47 | .143 |  | **-0.13** | **-2.46** | **.014** |  | 0.03 | 0.58 | .565 |  | -0.04 | -0.75 | .454 |  | **0.17** | **3.62** | **<.001** |
| Positive Reappraisal |  | 0.01 | 0.15 | .883 |  | -0.08 | -1.35 | .177 |  | **-0.16** | **-2.51** | **.012** |  | **-0.14** | **-2.40** | **.017** |  | **0.26** | **4.54** | **<.001** |
| Putting into Perspective |  | 0.01 | 0.15 | .878 |  | 0.09 | 1.53 | .127 |  | 0.05 | 0.84 | .404 |  | 0.07 | 1.20 | .233 |  | -0.06 | -1.09 | .278 |
| Catastrophizing |  | **0.31** | **4.31** | **<.001** |  | **0.29** | **3.82** | **<.001** |  | **0.20** | **2.76** | **.006** |  | **0.28** | **3.92** | **<.001** |  | **-0.25** | **-3.61** | **<.001** |
| Other-Blame |  | **0.18** | **3.87** | **<.001** |  | 0.10 | 2.01 | .045 |  | 0.04 | 0.78 | .438 |  | **0.14** | **3.10** | **.002** |  | -0.08 | -1.79 | .074 |

*Note:* bold indicates significance.

**Table S7**

*Behavioral Coping Subscales Seeking Distraction, Withdrawal, Approach, Social Support, and Ignoring Predict Moral Injury, Depression, Generalized Anxiety, Complex PTSD, and Wellbeing Beyond Demographics*

| **Predictors** |  | **Moral Injury** | | |  | **Depression** | | |  | **Generalized Anxiety** | | |  | **Complex PTSD** | | |  | **Wellbeing** | | |
| --- | --- | --- | --- | --- | --- | --- | --- | --- | --- | --- | --- | --- | --- | --- | --- | --- | --- | --- | --- | --- |
|  |  | **β** | ***t*** | ***p*** |  | **β** | ***t*** | ***p*** |  | **β** | ***t*** | ***p*** |  | **β** | ***t*** | ***p*** |  | **β** | ***t*** | ***p*** |
| **Step 1** |  |  |  |  |  |  |  |  |  |  |  |  |  |  |  |  |  |  |  |  |
| Age |  | -0.12 | -2.19 | .029 |  | **-0.20** | **-3.74** | **<.001** |  | **-0.20** | **-3.81** | **<.001** |  | **-0.16** | **-3.16** | **.002** |  | 0.06 | 1.06 | .288 |
| Gender |  | 0.00 | 0.08 | .935 |  | 0.01 | 0.31 | .759 |  | 0.09 | 1.85 | .065 |  | 0.01 | 0.13 | .895 |  | 0.04 | 0.85 | .396 |
| Relationship Status  (1 = *partnered*,  0 = *not partnered*) |  | -0.04 | -0.67 | .504 |  | -0.04 | -0.68 | .500 |  | 0.03 | 0.50 | .621 |  | -0.01 | -0.27 | .788 |  | 0.09 | 1.65 | .100 |
| Parent of <18  (1=*yes*, 0=*no*) |  | 0.05 | 0.90 | .367 |  | -0.04 | -0.83 | .409 |  | 0.01 | 0.14 | .889 |  | -0.02 | -0.34 | .735 |  | -0.05 | -1.00 | .320 |
| Caregiver >18  (1=*yes*, 0=*no*) |  | 0.00 | 0.08 | .938 |  | 0.07 | 1.37 | .171 |  | 0.09 | 1.95 | .052 |  | 0.06 | 1.31 | .190 |  | -0.09 | -1.79 | .074 |
| Therapy Experience  (1=*yes*, 0=*no*) |  | **0.23** | **4.49** | **<.001** |  | **0.27** | **5.51** | **<.001** |  | **0.28** | **5.77** | **<.001** |  | **0.35** | **7.18** | **<.001** |  | **-0.30** | **-5.87** | **<.001** |
| Religiosity |  | **0.12** | **2.40** | **.017** |  | 0.04 | 0.86 | .388 |  | 0.06 | 1.26 | .207 |  | 0.04 | 0.75 | .455 |  | -0.03 | -0.68 | .500 |
| **Step 2** |  |  |  |  |  |  |  |  |  |  |  |  |  |  |  |  |  |  |  |  |
| Seeking Distraction |  | 0.06 | 1.15 | .250 |  | -0.05 | -0.97 | .334 |  | -0.02 | -0.46 | .649 |  | -0.03 | -0.68 | .497 |  | 0.09 | 2.04 | .042 |
| Withdrawal |  | **0.31** | **5.41** | **<.001** |  | **0.43** | **8.76** | **<.001** |  | **0.44** | **8.93** | **<.001** |  | **0.51** | **10.75** | **<.001** |  | **-0.40** | **-8.72** | **<.001** |
| Approach |  | 0.07 | 1.24 | .215 |  | -0.10 | -1.81 | .072 |  | **-0.17** | **-3.30** | **.001** |  | -0.06 | -1.20 | .231 |  | **0.32** | **6.81** | **<.001** |
| Social Support |  | -0.01 | -0.18 | .855 |  | -0.07 | -1.30 | .198 |  | 0.06 | 1.13 | .259 |  | -0.01 | -0.12 | .903 |  | 0.08 | 1.77 | .078 |
| Ignoring |  | 0.08 | 1.29 | .199 |  | 0.08 | 1.52 | .126 |  | 0.01 | 0.18 | .854 |  | 0.08 | 1.52 | .129 |  | 0.05 | 1.00 | .335 |

*Note:* bold indicates significance.

**Table S8**

*Positive and Negative Religious Coping Predicts Moral Injury, Depression, Generalized Anxiety, Complex PTSD, and Wellbeing Beyond Demographics*

| **Predictors** |  | **Moral Injury** | | |  | **Depression** | | |  | **Generalized Anxiety** | | |  | **Complex PTSD** | | |  | **Wellbeing** | | |
| --- | --- | --- | --- | --- | --- | --- | --- | --- | --- | --- | --- | --- | --- | --- | --- | --- | --- | --- | --- | --- |
|  |  | **β** | ***t*** | ***p*** |  | **β** | ***t*** | ***p*** |  | **β** | ***t*** | ***p*** |  | **β** | ***t*** | ***p*** |  | **β** | ***t*** | ***p*** |
| **Step 1** |  |  |  |  |  |  |  |  |  |  |  |  |  |  |  |  |  |  |  |  |
| Age |  | -0.12 | -2.19 | .029 |  | **-0.20** | **-3.74** | **<.001** |  | -0.20 | -3.81 | **<.001** |  | **-0.16** | **-3.16** | **.002** |  | 0.06 | 1.06 | .288 |
| Gender |  | 0.00 | 0.08 | .935 |  | 0.01 | 0.31 | .759 |  | 0.09 | 1.85 | .065 |  | 0.01 | 0.13 | .895 |  | 0.04 | 0.85 | .396 |
| Relationship Status  (1 = *partnered*,  0 = *not partnered*) |  | -0.04 | -0.67 | .504 |  | -0.04 | -0.68 | .500 |  | 0.03 | 0.50 | .621 |  | -0.01 | -0.27 | .788 |  | 0.09 | 1.65 | .100 |
| Parent of <18  (1=*yes*, 0=*no*) |  | 0.05 | 0.90 | .367 |  | -0.04 | -0.83 | .409 |  | 0.01 | 0.14 | .889 |  | -0.02 | -0.34 | .735 |  | -0.05 | -1.00 | .320 |
| Caregiver >18  (1=*yes*, 0=*no*) |  | 0.00 | 0.08 | .938 |  | 0.07 | 1.37 | .171 |  | 0.09 | 1.95 | .052 |  | 0.06 | 1.31 | .190 |  | -0.09 | -1.79 | .074 |
| Therapy Experience  (1=*yes*, 0=*no*) |  | **0.23** | **4.49** | **<.001** |  | **0.27** | **5.51** | **<.001** |  | 0.28 | 5.77 | **<.001** |  | **0.35** | **7.18** | **<.001** |  | **-0.30** | **-5.87** | **<.001** |
| Religiosity |  | **0.12** | **2.40** | **.017** |  | 0.04 | 0.86 | .388 |  | 0.06 | 1.26 | .207 |  | 0.04 | 0.75 | .455 |  | -0.03 | -0.68 | .500 |
| **Step 2** |  |  |  |  |  |  |  |  |  |  |  |  |  |  |  |  |  |  |  |  |
| Positive Religious Coping |  | 0.13 | 1.47 | .142 |  | -0.12 | -1.40 | .161 |  | -0.17 | -2.15 | .032 |  | -0.16 | -1.94 | .053 |  | **0.20** | **2.36** | **.019** |
| Negative Religious Coping |  | 0.14 | 2.09 | .038 |  | **0.32** | **4.99** | **<.001** |  | 0.36 | 5.72 | **<.001** |  | **0.37** | **5.95** | **<.001** |  | **-0.32** | **-4.94** | **<.001** |

*Note:* bold indicates significance.

**Discussion**

These results indicate that UK online participants recruited via Prolific suffer from a elevated levels of anxiety, depression, PTSD, and lower wellbeing—significantly worse outcomes even than UK police officers and staff dealing with child sexual abuse and exploitation (CSAE), except for anxiety rates which were similarly elevated across both groups. Although inconsistent with predictions, as we originally expected the police sample to demonstrate worse mental health than the online sample, these findings in fact align with a growing body of work showing that online workers suffer from elevated mental health issues than the general population (e.g., Engel et al., 2020; McCredie, & Morey, 2019; Ophir et al., 2020).

Consider that online work is contractual, temporary, low pay, and completed alone, online workers may lack interpersonal and organizational resources that predicted better outcomes in the police sample. In addition, the online sample was less likely to be partnered or have children than the police sample, suggesting lower support. Although we did not measure income or socioeconomic status, considering the UK police are salaried, these factors may be important. Importantly, in most other demographics, the online and police samples scored similarly.

In terms of prediction, there was largely consistency across the police and online samples. This pattern increases confidence in the robustness of effects. In both samples, demographics largely failed to predict outcomes except for therapy experience and to some degree religiosity. However, in the online sample younger people also reported worse outcomes. In both samples moral injury betrayal was a strong predictor of outcomes. In both samples, Self-blame, other-blame, catastrophizing, and withdrawal predicted worse outcomes, whereas planning and positive reappraisal predicted better outcomes. However, unlike the police sample, there was no significant effects for rumination, putting into perspective, positive refocusing, seeking distraction, social support, or ignoring. Finally, negative religious coping predicted worse outcomes in both samples, though in the online sample positive religious coping also predicted better wellbeing.

Tother, these patterns may suggest that police dealing with CSAE material may have more success when compartmentalizing their work problems and distracting themselves through nonwork activities. Conversely, the online sample may be dealing with issues that impact their lives more broadly and hence are less conductive to this compartmentalization approach. However, the online sample may find more solace in religion than the police sample.

**Limitations**

This study is of course a quasi-experiment because we could not randomly assign participants to police versus civilian conditions, limiting the ability to draw causal inferences. Moreover, the comparability between UK police and civilian online samples remains somewhat unclear. One sample was recruited via requests from workplace superiors and specifically discussed workplace related issues, whereas the other self-selected into the study via Prolific and described somewhat more general issues. We also assessed the online sample a couple months later than the police sample due to technical challenges, introducing thew possibility that global events may have impacted responses in the interim. These samples may also differ on factors we did not measure, such as locations within the UK, or socioeconomic status. On the other hand, the demographic differences between these samples were few; they had similar distributions of age, gender, partnership, faith, caregiving, therapy experience, and other relevant variables. The primary exceptions were higher rates of partnering and parenting in the police than online sample. These factors suggest the samples are rather comparable. Moreover, the predictive patterns in the samples were generally similar comparable, and results comparing them held controlling for demographic factors. Therefore, these samples may be reasonably compared with caution.

**References**

Engle, K., Talbot, M., & Samuelson, K. W. (2020). Is Amazon’s Mechanical Turk (MTurk) a comparable recruitment source for trauma studies? *Psychological Trauma: Theory, Research, Practice, and Policy, 12*(4), 381.

McCredie, M. N., & Morey, L. C. (2019). Who are the Turkers? A characterization of MTurk workers using the personality assessment inventory. *Assessment, 26*(5), 759-766.

Ophir, Y., Sisso, I., Asterhan, C. S., Tikochinski, R., & Reichart, R. (2020). The Turker blues: Hidden factors behind increased depression rates among Amazon’s Mechanical Turkers. *Clinical Psychological Science, 8*(1), 65-83.

Sharman, S., Roberts, A., Bowden-Jones, H., & Strang, J. (2021). Gambling in COVID-19 lockdown in the UK: Depression, stress, and anxiety. *Frontiers in Psychiatry*, *12*, 621497.
