# Supplementary material for: Protecting the Protectors: Moral Injury, Coping Styles, and Mental Health of UK Police Officers and Staff Investigating Child Sexual Abuse Material
Source: Depress Anxiety. 2024 Nov 23;2024:1854312. doi: 10.1155/da/1854312 (PMC11922302; doi:10.1155/da/1854312)
Supplement: Supporting Information 3 — File S3: Study Materials provides the exact wording of all measures, instructions, and materials in the study, as well as a copy of the recruitment email sent to participants. [file 1854312.f3.docx]

**Online Supplemental Material: Study Materials & Recruitment**

**Protecting the Protectors: Moral Injury, Coping Styles, and Mental Health of UK Police Officers and Staff Dealing with Child Sexual Assault and Exploitation**

**Demographics**

How **old**are you in years? ______

What **gender** do you most identify with? ________

What is your marital status? *Single, married, separated, divorced, living as married, widowed*

Which **ethnicity**do you most identity with? ___________

Are you part of the **police officer or police staff?** Feel free to clarify. _______

What is your **rank** (if police) or **job title** (if police staff)? ________

What **region** do you work in?

Southeast

Southwest

London

East Midlands

West Midlands

Eastern

Northeast

Northwest

Wales

Scotland

Northern Ireland

Other (e.g., Yorkshire, National)

Unreported

What best describes your **role**? Select any that apply.

Child Protection Unit

Investigations

POLIT (Police Online Investigation Team)

Intelligence

MOSOVO (Management of Sexual or Violent Offenders)

Operational Support

Administrative Support

Criminal Justice

Neighborhood Policing

National Policing

Response Policing

Training

Other (please specify)

How long have you **worked in your current role**?

Response scale: 1 = *only a few days* – 9 = *over 20 years*

How long have you **worked as a police officer or police staff**?

Response scale: 1 = *only a few days* – 9 = *over 20 years*

**Are you a parent**, step parent, foster parent, or otherwise have guardian responsibilities for any children under the age of 18?

Yes/No/It’s complicated <space to clarify>

**Are you a caregiver** to anyone over 18 (e.g., elderly parents)?

Yes/No/It’s complicated <space to clarify>

How **spiritual or religious**are you?

1 = *not at all spiritual/religious* – 7 = *Extremely spiritual/religious*

**What faith**do you most identify with, such as Christianity, Islam, Agnostic, or Spiritual but not religious? ______

Have you previously, or are you currently receiving psychological support or **therapy**? _______

(coded 1 = *any experience*, 0 = *no experience*)

**Risk and Protective Factors**

When thinking about your involvement with investigations into child sexual abuse and exploitation (CSAE), how often have you encountered the following situations?

1 (*almost never*) to 7 (*almost always*).

**Risk factors**

Viewing, grading, or handling photos of child sexual abuse and exploitation (CSAE)

Viewing, grading, or handling videos of child sexual abuse and exploitation (CSAE)

Contact (in person, phone, internet) with victims

Contact (in person, phone, internet) with families of victims

Contact (in person, phone, internet) with perpetrators or suspects

**Protective factors – Successes**

Succeeding in protecting a child from child sexual abuse and exploitation (CSAE)

Assisting with arrests or convictions for child sexual abuse and exploitation (CSAE)

Supporting victims and/or their families understand and recover from child sexual abuse and exploitation (CSAE)

Feeling my work is valuable and meaningful

**Protective factors – Support**

Feeling encouraged to seek mental health support from my supervisor or team

Feeling supported by my supervisor or team

Feeling supported by my partner, friends, or family

Taking time to rest and pursue activities outside of work

Feeling like my voice is heard and I have input into decisions that affect me

**Open-ended response**

Is there anything else that you routinely do in your role that you want us to know about?

**The Moral Injury Events Scale** (adapted from Nash et al., 2013)

Often when dealing with sensitive material people see or do things that leave them questioning morality.

For example, sometimes actions can have both good and bad aspects.

People’s experiences of morality can influence their well-being, so we are asking about your feelings about morality to better understand how we can help people who are struggling.

When thinking about **your involvement with investigations into child sexual abuse and exploitation (CSAE),** how much do you agree with the following statements?

Response scale: 1 (*strongly disagree*) to 7 (*strongly agree*).

**Transgressions-Other**

I saw things that were morally wrong.

I am troubled by having witnessed others’ immoral acts

**Transgressions-Self**

I acted in ways that violated my own moral code or values

I am troubled by having acted in ways that violated my own morals or values

I violated my own morals by failing to do something that I felt I should have done

I am troubled because I violated my morals by failing to do something I felt I should have done

**Betrayal**

I feel betrayed by leaders who I once trusted

I feel betrayed by colleagues who I once trusted

I feel betrayed by others outside the police who I once trusted

**Short Cognitive and Emotion Regulation Questionnaire** (Garnefski & Kraaij, 2006)

People sometimes feel stressed or upset about experiences they have.

When thinking about **your involvement with investigations into child sexual abuse and exploitation** (CSAE), how often do you have each thought or experience?

Responses: 1 (*almost never*) to 7 (*almost always*).

**Self-blame**

I feel that I am the one who is responsible for what has happened

I think that basically the cause must lie within myself

**Acceptance**

I think that I have to accept that this has happened

I think that I have to accept the situation

**Focus on thought/rumination**

I often think about how I feel about what I have experienced

I am preoccupied with what I think and feel about what I have experienced

**Positive refocusing**

I think about how I can best cope with the situation

I think about how to change the situation

**Refocus on planning**

I think about how to change the situation

I think about a plan of what I can do best

**Positive reappraisal**

I think that the situation also has its positive sides

I look for the positive sides to the matter

**Putting into perspective**

I think that it hasn’t been too bad compared to other things

I tell myself that there are worse things in life

**Catastrophizing**

I keep thinking about how terrible it is what I have experienced

I continually think how horrible the situation has been

**Other Blame**

I feel that others are responsible for what has happened

I feel that basically the cause lies with others

**Short Behavioural Emotion Regulation Questionnaire** (adapted from Kraaij & Garnefski, 2019)

People sometimes take actions when they feel stressed or upset about experiences they have.

When thinking about **your involvement with investigations into child sexual abuse and exploitation**(CSAE), how often do perform each behaviour?

Responses: 1 (*almost never*) to 7 (*almost always*)

**Seeking Distraction**

I set my worries aside by doing something else

I do other things to distract myself

**Withdrawal**

I withdraw

I isolate myself

**Actively Approaching**

I get to work on it

I take action to deal with it

**Seeking Social Support**

I share my feelings with someone

I look for someone who can support me

**Ignoring**

I repress it and pretend it never happened

I behave as if nothing is going on

**The Brief Religious Coping Scale RCOPE** (Pargament et al., 2011)

Most people have **some concept of God, religion, or spirituality,** even if they do not personally believe in such things. We are interested in your thoughts **however you understand such things**. Some people report not being religious or spiritual, which is fine, but we still want to hear from you.

Dealing with sexually explicit material involving children may lead some people to question or reinforce their beliefs about God, religion, or spirituality. We want to understand if, and how, such beliefs can influence mental health and wellbeing when dealing with child sexual abuse and exploitation.

When thinking about **your involvement with investigations into child sexual abuse and exploitation** (CSAE), how often do you experience the following?

1 = *Almost never -* 7 = *Almost always* (note: we changed the scale to match the CERQ & BERQ)

**Positive Religious Coping Subscale Items**

1. Looked for a stronger connection with God

2. Sought God’s love and care

3. Sought help from God in letting go of my anger

4. Tried to put my plans into action together with God

5. Tried to see how God might be trying to strengthen me in this situation

6. Asked forgiveness for my sins

7. Focused on religion to stop worrying about my problems

**Negative Religious Coping Subscale Items**

8. Wondered whether God had abandoned me

9. Felt punished by God for my lack of devotion

10. Wondered what I did for God to punish me

11. Questioned God’s love for me

12. Wondered whether my church had abandoned me

13. Decided the devil made this happen

14. Questioned the power of God

**Patient Health Questionnaire – Depression**(Kroenke, Spitzer, & Williams, (2001)

Over the **last month, how often have you been bothered** by any of the following problems?

Response scale: 0 = *not at all*, 1 = *several days*, 2 = *more than half the days*, 3 = *nearly every day*

Little interest or pleasure in doing things

Feeling down, depressed, or hopeless

Trouble falling or staying asleep, or sleeping too much

Feeling tired or having little energy

Poor appetite or overeating

Feeling bad about yourself—or that you are a failure or have let yourself or your family down

Trouble concentrating on things, such as reading the news or watching television

Moving or speaking so slowly that other people could have noticed, or the opposite—being so fidgety or restless that you have been moving around a lot more than usual

Thoughts that you would be better off dead or hurting yourself in some way

If you are experiencing any of these problems, **how difficult has it been** for you to do your work, take care of things at home, and get along with other people?

Response scale: 1 = *Not difficult at all* – 4 = *very difficult*

**Generalized Anxiety Disorder** (Spitzer, Kroenke, Williams, & Lowe, 2006)

Over the **last month, how often have you been bothered** by any of the following problems?

Response scale: 0 = *not at all*, 1 = *several days*, 2 = *more than half the days*, 3 = *nearly every day*

Feeling nervous, anxious, or on edge

Not being able to stop or control worrying

Worrying too much about different things

Trouble relaxing

Being so restless that it is hard to sit still

Becoming easily annoyed or irritable

Feeling afraid as if something awful might happen

**Attention Check**

If you are paying attention, please select 'nearly every day'

**The International Trauma Questionnaire (ITQ)** (adapted from Cloitre et al., 2018).

**PTSD Factor**

Below are a number of problems that people sometimes report in response to traumatic or stressful life events, including investigating childhood sexual abuse and exploitation.

When thinking about your involvement with investigations into child sexual abuse and exploitation (CSAE), **how much have you been bothered by each problem over the past month?**

Response scale: 0 = *not at all* - 4 = *extremely*

Having upsetting dreams that replay part of the experience or are clearly related to the experience?

Having powerful images or memories that sometimes come into your mind in which you feel the experience is happening again in the here and now?

Avoiding internal reminders of the experience (for example, thoughts, feelings, or physical sensations).

Avoiding external reminders of the experience (for example, people, places, conversations, objects, activities, or situations)?

Being “super-alert”, watchful, or on guard?

Feeling jumpy or easily startled?

**Over the past month**, how much have these symptoms:

Affected your relationships or social life?

Affected your work or ability to work?

Affected any other important part of your life such as parenting, or school or college work, or other important activities?

**Disturbance in Self Factor**

Below are a number of problems that people sometimes report in response to traumatic or stressful life events, including investigating child sexual exploitation.

The questions refer to ways you **typically feel**, ways you **typically think**about yourself and ways you **typically relate** to others. Answer the following thinking about how true each statement is of you.

Response scale: 0 = *not at all* - 4 = *extremely*

When I am upset, it takes me a long time to calm down.

I feel numb or emotionally shut down.

I feel like a failure.

I feel worthless

I feel distant or cut off from people

I find it hard to stay emotionally close to people.

**Over the past month,** how much have these problems in emotions, in beliefs about yourself and in relationships:

Created concern or distress about your relationships or social life?

Affected your work or ability to work?

Affected any other important parts of your life such as parenting, or school or college work, or other important activities?

**Schwartz Outcome Scale** (Blais et al., 1999).

Please choose the response that best fits how you have been feeling over the past month.

Response Options: 1 (*almost* *never*) to 7 (*almost always*)

Given my current physical condition, I am satisfied with what I can do.

I have confidence in my ability to sustain important relationships.

I feel hopeful about my future.

I am often interested and excited about things in my life.

I am able to have fun.

I am generally satisfied with my psychological health.

I am able to forgive myself for my failures.

My life is progressing according to my expectations.

I am able to handle conflicts with others.

I have peace of mind.

**Current Resource Provision**

What mental health and wellbeing or personal support does your force currently provide?

Select any that apply. Feel free to clarify if you like.

Occupational Health Therapist; external/self- referred counselling; peer-support program; mental health or wellbeing days off; clinical supervision, Oscar Kilo, Wellbeing of Investigators Toolkit, other

**Current Resource Usage**

How often have you used each mental health or wellbeing resource?

Response Options: 1 *(almost never*) to 7 (*almost always*)

Occupational Health Therapist; external/self- referred counselling; peer-support program; mental health or wellbeing days off; clinical supervision, Oscar Kilo, Wellbeing of Investigators Toolkit, other

**Current Resource Helpfulness**

How helpful or useful have found each resource?

Response Options: 1 (*very unhelpful*) to 7 (*very helpful*)

Occupational Health Therapist; external/self- referred counselling; peer-support program; mental health or wellbeing days off; clinical supervision, Oscar Kilo, Wellbeing of Investigators Toolkit, other

**Current Barriers to Resource Use**

Thinking about possible barriers that might stop you from asking for mental health or wellbeing support at work, how much is each of the following a barrier for you?

Response Options: 1 (*Not at all*) to 7 (*A large amount*)

I feel pressured to seem strong in front of colleagues

Workplace culture where weakness seems not allowed

Wanting to seem capable of handling stress and performing well

It seems like everyone else can cope with this job

Worried that getting support might undermine my job prospects

I'm not convinced that seeking support will help and it might even cause problems

I don't trust my organization to keep my support confidential

Is there anything else you would like to add about possible barriers to seeking support (optional)? ___________________

**Desired Support**

Which of the following mental health or wellbeing support services do you think would be helpful when you face challenges at work? (Select any that apply)

Monthly group sessions with team

Monthly wellness check-in

24/7 access to support

Limiting daily exposure to child sexual abuse materials

Separating viewing online CSAM tasks and interviewing victims and/or perpetrators

A workplace culture that explicitly values and prioritises the emotional health and wellbeing of the workforce

A wellness room

Informal peer support

Wellness events (workshops, training)

Wellness plans

Social activities

Mindfulness sessions

Clinical supervision

Self-referral to funded counselling (separate from work)

Are there any other mental health or wellbeing support services you think would be helpful? (optional) ________________

**Study Recruitment Email**
