# Supplementary material for: Protecting the Protectors: Moral Injury, Coping Styles, and Mental Health of UK Police Officers and Staff Investigating Child Sexual Abuse Material
Source: Depress Anxiety. 2024 Nov 23;2024:1854312. doi: 10.1155/da/1854312 (PMC11922302; doi:10.1155/da/1854312)
Supplement: Supporting Information 4 — File S4: Resources and Barriers provides the methods and results of additional measures included in the study but not in the main text, assessing perceptions of resource availability, use, helpfulness, and barriers. [file 1854312.f4.docx]

**Online Supplemental Materials: Resource Availability, Use, Helpfulness, and Barriers**

**Protecting the Protectors: Moral Injury, Coping Styles, and Mental Health of UK Police Officers and Staff Investigating Child Sexual Abuse Material**

**Method**

***Resource Availability, Use, and Helpfulness***

Participants reported whether each of seven resources were available to them (0 = *no*, 1 = *yes*) and how often they use each resource on scales from 1 (*Never or Almost never*) to 7 (*Always or Almost always*): *Occupational Health Therapist,* *External/Self-referred counselling*, *Peer support program*, *Mental health or wellbeing days off*, *Clinical Supervision*, *Oscar Kilo* [Police Counselling Service], *Wellbeing of Investigators Toolkit*. We computed the mean (α = .66). Participants also reported how helpful they viewed each resource on scales from 1 (*Very unhelpful*) to 7 (*Very helpful*, α = .87). We excluded participant responses when they selected 8 (*not applicable*). We computed the mean.

***Barriers to Seeking Support***

We asked participants: *Thinking about possible barriers that might stop you from asking for mental health or wellbeing support at work, how much is each of the following a barrier for you?* They responded to seven items on scales from 1 (*Not at all*) to 7 (*A large amount*), such as *I feel pressured to seem strong in front of colleagues* and *Worried that getting support might undermine my job prospects*. We conducted a principal components analysis with oblimin rotation allowing for 500 iterations before convergence and 500 for rotation, retaining all factors with an eigenvalue greater than 1 (Kaiser, 1961) that are readily interpretable (Lee & Ashton, 1995). Although we anticipated a 2-factor solution, all items loaded on a single factor with an eigenvalue greater than 1 (4.51) accounting for 64.45% of variance in the scale. Therefore, we took the mean across all items (α = .91). We also asked an open-ended item: *Is there anything else you would like to add about possible barriers to seeking support (optional)?*

***Desired Resources***

Finally, we asked participants to check off resources they thought would be helpful and report additional desired resources via an open-ended item. We report a detailed breakdown and qualitative analysis of resource availability, use, helpfulness, barriers, and desired resources, including a qualitative analysis of open-ended responses elsewhere (<masked> et al., 2023). Here we examined how quantitative measures predicted overall responses to these measures.

**Results**

***Resource Availability, Use, Helpfulness, Barriers, and Desired Resources***

We computed the number and percent of participants reporting access to each resource, along with reports of how often people used each resource and how helpful each was (see Table S1). Participants reported the most access to Occupational Health Therapists, followed by External/self-referred counselling, Peer-support programs, Oskar Kilo, the Wellbeing of Investigators Toolkit, Mental health or wellbeing days off, and finally the fewest reported Clinical supervision. Few participants reported using resources, with most services averaging a 1 or 2 out of 7, with the highest usage ratings for Occupational Health Therapists. Participants reported External/self-referred counselling as the most useful, followed by Occupational Health Therapists, Mental health or wellbeing days off, Peer-support programs, the Wellbeing of Investigators Toolkit, Oskar Kilo, and finally clinical supervision.

Participants reported how must various barriers posed a challenge to their seeking of support (see Table S2). They rated Wanting to seem capable of handling stress and performing well as the highest barrier, followed by I feel pressured to seem strong in front of colleagues, It seems like everyone else can cope with this job, Worried that getting support might undermine my job prospects, I'm not convinced that seeking support will help and it might even cause problems, I don't trust my organization to keep my support confidential, and finally Workplace culture where weakness seems not allowed.

Participants also reported whether they desired various resources (see Table S3). The highest ratings were A workplace culture that explicitly values and prioritizes the emotional health and wellbeing of the workforce, followed by social activities, Self-referral to funded counselling (separate from work), Monthly wellness check-in, and 24/7 access to support, with nearly half of participants endorsing each of these resources. Fewer participants endorsed resources like Monthly group sessions with team, Wellness plans, clinical supervision, or Separating viewing online CSAE tasks and interviewing victims and/or perpetrators.

**Table S1**

*Resource Access, Usage, and Helpfulness*

|  | Provided (0=*no*, 1=*yes*) | | Used (1-7) | | Helpful (1-7) | |
| --- | --- | --- | --- | --- | --- | --- |
| Resource | *N* | Percent | *M* | *SD* | *M* | *SD* |
| Occupational Health Therapist | 535 | 80.9 | 2.11 | 1.43 | 3.90 | 1.91 |
| External/self-referred counselling | 395 | 59.8 | 1.94 | 1.47 | 4.53 | 2.13 |
| Peer-support program | 304 | 46.0 | 1.32 | 0.96 | 3.46 | 2.20 |
| Mental health or wellbeing days off | 118 | 17.9 | 1.31 | 0.98 | 3.72 | 2.45 |
| Clinical supervision | 33 | 5.0 | 1.16 | 0.82 | 2.22 | 1.89 |
| Oscar Kilo | 239 | 36.2 | 1.17 | 0.72 | 2.64 | 1.98 |
| Wellbeing of Investigators Toolkit | 166 | 25.1 | 1.22 | 0.78 | 2.95 | 1.98 |
| Other | 78 | 11.8 | - | - | - | - |

**Table S2**

*Barriers to Support*

| Barrier | *M* | *SD* |
| --- | --- | --- |
| I feel pressured to seem strong in front of colleagues | 3.93 | 2.09 |
| Workplace culture where weakness seems not allowed | 3.16 | 2.04 |
| Wanting to seem capable of handling stress and performing well | 4.63 | 2.03 |
| It seems like everyone else can cope with this job | 3.76 | 2.08 |
| Worried that getting support might undermine my job prospects | 3.55 | 2.31 |
| I'm not convinced that seeking support will help and it might even cause problems | 3.25 | 2.15 |
| I don't trust my organization to keep my support confidential | 3.22 | 2.31 |

**Table S3**

*Desired Resources*

| Resource | *N* | Percent |
| --- | --- | --- |
| Monthly group sessions with team | 222 | 33.6 |
| Monthly wellness check-in | 320 | 48.4 |
| 24/7 access to support | 314 | 47.5 |
| Limiting daily exposure to child sexual abuse materials | 194 | 29.3 |
| Separating viewing online CSAE tasks and interviewing victims and/or perpetrators | 88 | 13.3 |
| A workplace culture that explicitly values and prioritizes the emotional health and wellbeing of the workforce | 400 | 60.5 |
| A wellness room | 248 | 37.5 |
| Informal peer support | 186 | 28.1 |
| Wellness events (workshops, training) | 262 | 39.6 |
| Wellness plans | 151 | 22.8 |
| Social activities | 388 | 58.7 |
| Mindfulness sessions | 271 | 41.0 |
| Clinical supervision | 145 | 21.9 |
| Self-referral to funded counselling (separate from work) | 326 | 49.3 |

***Predicting Support Access, Usage, Helpfulness, and Barriers***

Next, we conducted exploratory analyses to examine factors that predicted a) reported access to support services, b) reported use of support services, c) ratings of the helpfulness of support service, and d) reports of barriers to using support services (each operationalized as the mean across all services examined). As these analyses were exploratory, we did not preregister hypotheses. Each analysis employed the same regression strategy as the main analysis, using the same set of control variables at step 1 and each set of predictor variables at Step 2. For a detailed breakdown of access, usage, helpfulness, and barriers to specific sources of support, see <masked> and colleagues (2023) and online supplementary materials.

**Resource Access.** No demographic factors reached significance predicting access. Reported access to resources was not also significantly related to job risks or protective factors. Neither transgression-self nor betrayal were significant predictors, but people reporting higher transgression-other reported lower access, β = -.22, *p* = .013. Regarding the CERQ, BERQ, and religious coping, only rumination predicted reduced reported access, β = -.58, *p* = .014; no other cognitive, emotional, behavioral, or religious coping style was significant.

**Resource Use.** No demographic factors reached significance except age: younger people reported using more support, β = -.58, *p* = .007. Reported resource use was not significantly related to job risks or protective factors, nor any moral injury factors. Regarding the CERQ and BERQ, no cognitive, emotional, or behavioral coping style reached significance; nor did religious coping.

**Resource Helpfulness.** No demographic factors reached significance predicting resource helpfulness ratings. Helpfulness was also not significantly related to job risks or protective factors, nor to any moral injury factors. Regarding the CERQ and BERQ, people who reported resources less helpful scored high in rumination, β = -.83, *p* < .001; and catastrophizing, β = -.67, *p* < .001, and lower in positive reappraisal, β = .46, *p* = .002. Regarding the CERQ and BERQ, people high in ignoring reported resources less helpful, β = -.44, *p* = .022. No other cognitive, emotional, or behavioral coping style was significant; nor was religious coping.

**Resource Barriers.** No demographic factors reached significance predicting barriers. Reported barriers to resources were not significantly related to risks factors, but people who reported greater job success and support reported fewer barriers, β = .62, p < .001. Neither transgression-self nor transgression-other were significant predictors, but people reporting higher betrayal reported higher barriers, β = -.40, *p* = .004. Regarding the CERQ and BERQ and religious coping, only rumination predicted increased barriers, β = .51, *p* = .017; no other cognitive, emotional, behavioral, or religious coping style was significant.
