# Supplementary material for: Protecting the Protectors: Moral Injury, Coping Styles, and Mental Health of UK Police Officers and Staff Investigating Child Sexual Abuse Material
Source: Depress Anxiety. 2024 Nov 23;2024:1854312. doi: 10.1155/da/1854312 (PMC11922302; doi:10.1155/da/1854312)
Supplement: Supporting Information 5 — Appendix A. Data Transparency clarifies which analyses are unique to this paper and which overlap with Redmond et al. [23], who examined the same dataset. [file 1854312.f5.docx]

**Appendix A: Data Transparency**

The majority of data collected for this paper are presented only in the current manuscript, but a few variables (primarily demographic data) overlap with another paper. This companion manuscript provides a qualitative analysis of responses to questions in the current dataset regarding perceived availability, utility, and barriers to resources, along with summary statistics (Redmond et al., 2023). Hence, the two manuscripts share a sample but analyze primarily different questions with different methods. We provide a table clarifying which measures from the current dataset appear in which paper.

| **Measure** | **Current Manuscript** | **Redmond et al., 2023** |
| --- | --- | --- |
| Risk and Protective Factors | Quantitative results only | Qualitative responses only |
| The Moral Injury Events Scale | Yes | No |
| Short Cognitive and Emotion Regulation Questionnaire | Yes | No |
| Short Behavioral Emotion Regulation Questionnaire | Yes | No |
| The Brief Religious Coping Scale | Yes | No |
| Patient Health Questionnaire 9 | Yes | No |
| Generalized Anxiety Disorder | Yes | No |
| The International Trauma Questionnaire | Yes | No |
| Resource Provision | Count and percent and regressions predicting responses (supplement) | Count and percent and qualitative responses |
| Resource Use | Means and SDs and regressions predicting responses (supplement) | Means and SDs and qualitative responses |
| Resource Helpfulness | Means and SDs and regressions predicting responses (supplement) | Means and SDs and qualitative responses |
| Barriers to Resource Use | Means and SDs and regressions predicting responses (supplement) | Means and SDs and qualitative responses |
| Desired Support | Count and percent (supplement) | Count and percent and qualitative responses |
| Age | Yes | Yes |
| Gender | Yes | Yes |
| Relationship status | Yes | No |
| Ethnicity | Yes | Yes |
| Officer or staff | Yes | Yes |
| Rank or job title | Yes | No |
| Region | Yes | Yes |
| Role description | Yes | Yes |
| Role length | Yes | Yes |
| Career length | Yes | Yes |
| Parent | Yes | No |
| Caregiver | Yes | No |
| Religiosity | Yes | No |
| Faith | Yes | No |
| Therapy | Yes | No |
|  |  |  |
|  |  |  |
